# Supplementary material for: Telomeres control human telomerase (TERT) expression through non-telomeric TRF2
Source: eLife. 2025 Sep 30;14:RP104045. doi: 10.7554/eLife.104045 (PMC12483519; doi:10.7554/eLife.104045)

Supplementary Figure 1C Uncropped images

TRF2 protein induction with Dox treatment in HT1080, HCT116 and MDAMB 231 TRF2 inducible lentiviral stable cells, confirmed by Western blot analysis (Mol. Wt. ladder used in HT1080 and MDAMB 231 is Puregene 4 color Prestained Protein Ladder,10-180 kDa and that of HCT116 is G Biosciences PAGEmark Tricolor PLUS.

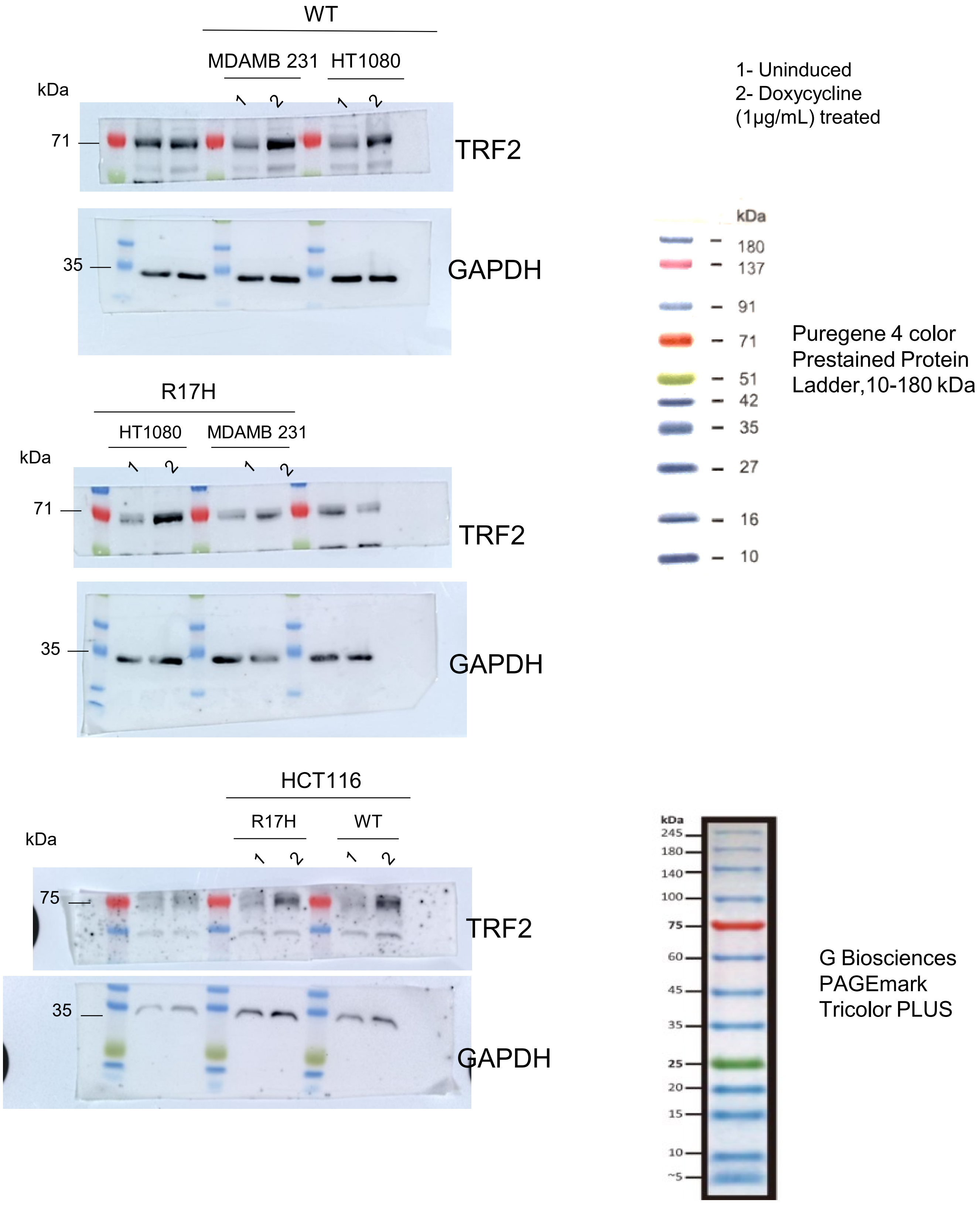

Supplement: Figure 9—figure supplement 1—source data 2. [file elife-104045-fig9-figsupp1-data2.pdf]
