# Supplementary figures and images for: Telomeres control human telomerase (TERT) expression through non-telomeric TRF2
Source: eLife. 2025 Sep 30;14:RP104045. doi: 10.7554/eLife.104045 (PMC12483519; doi:10.7554/eLife.104045)

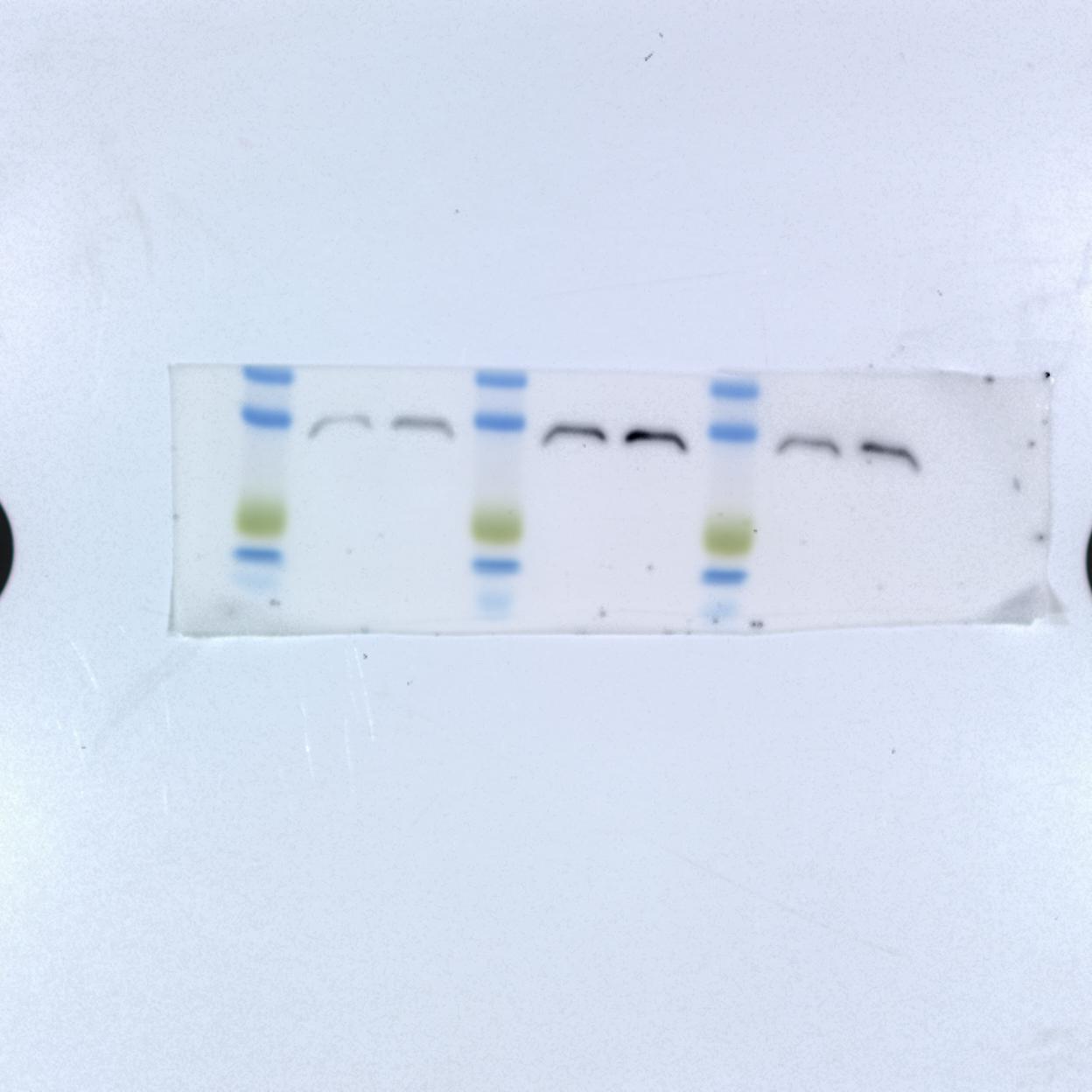

Supplement: Figure 9—figure supplement 1—source data 3. [file elife-104045-fig9-figsupp1-data3.zip › Figure 9-Figure supplement 1 Source data 3 (raw image of C)/hctind gap 20230427_033117_Ch+Marker.jpg]

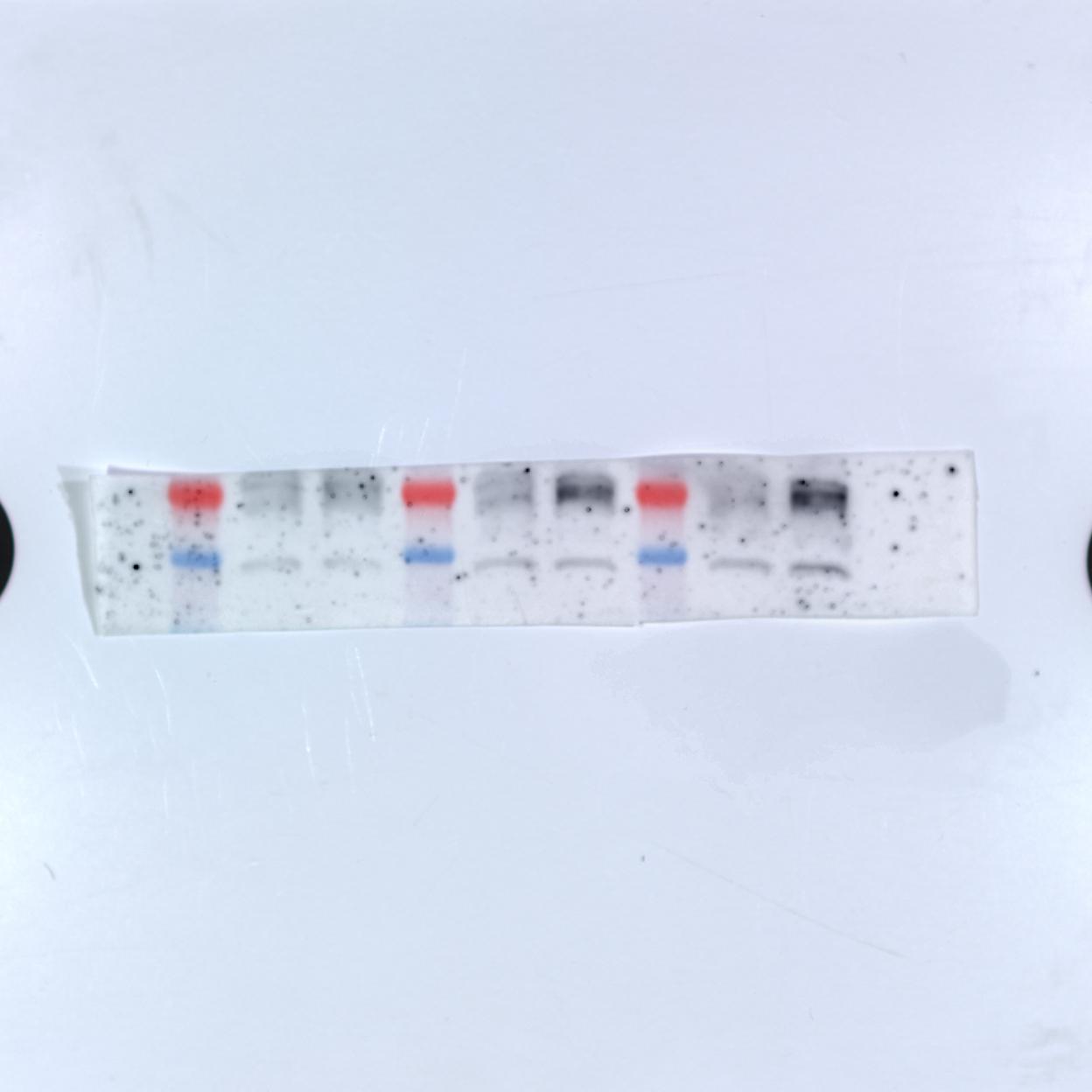

Supplement: Figure 9—figure supplement 1—source data 3. [file elife-104045-fig9-figsupp1-data3.zip › Figure 9-Figure supplement 1 Source data 3 (raw image of C)/hctindtrf2 20230427_034828_Ch+Marker.jpg]

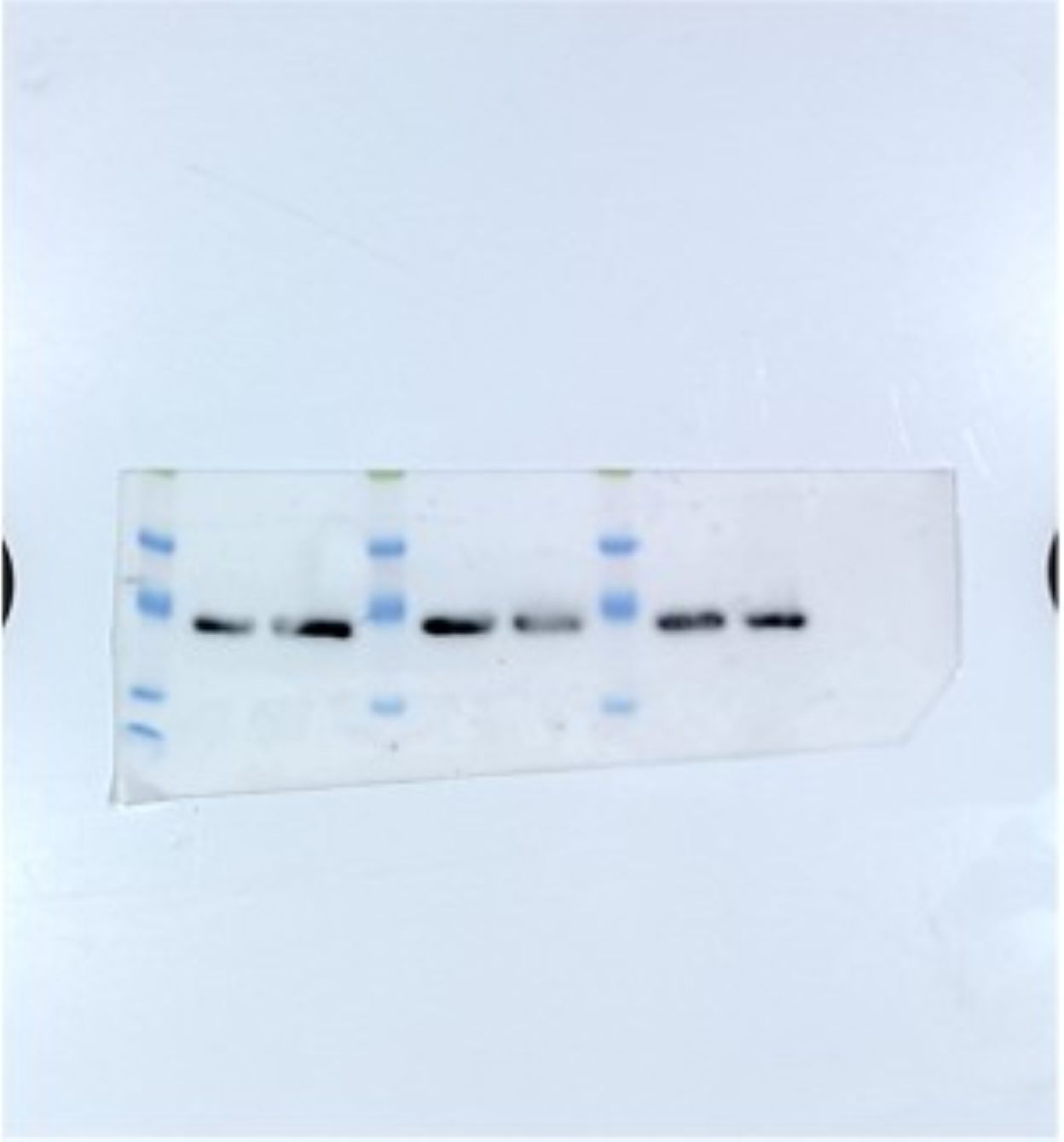

Supplement: Figure 9—figure supplement 1—source data 3. [file elife-104045-fig9-figsupp1-data3.zip › Figure 9-Figure supplement 1 Source data 3 (raw image of C)/HT1080 _MDAMB R17H INDUCIBLE GAPDH BLOT+marker.jpg]

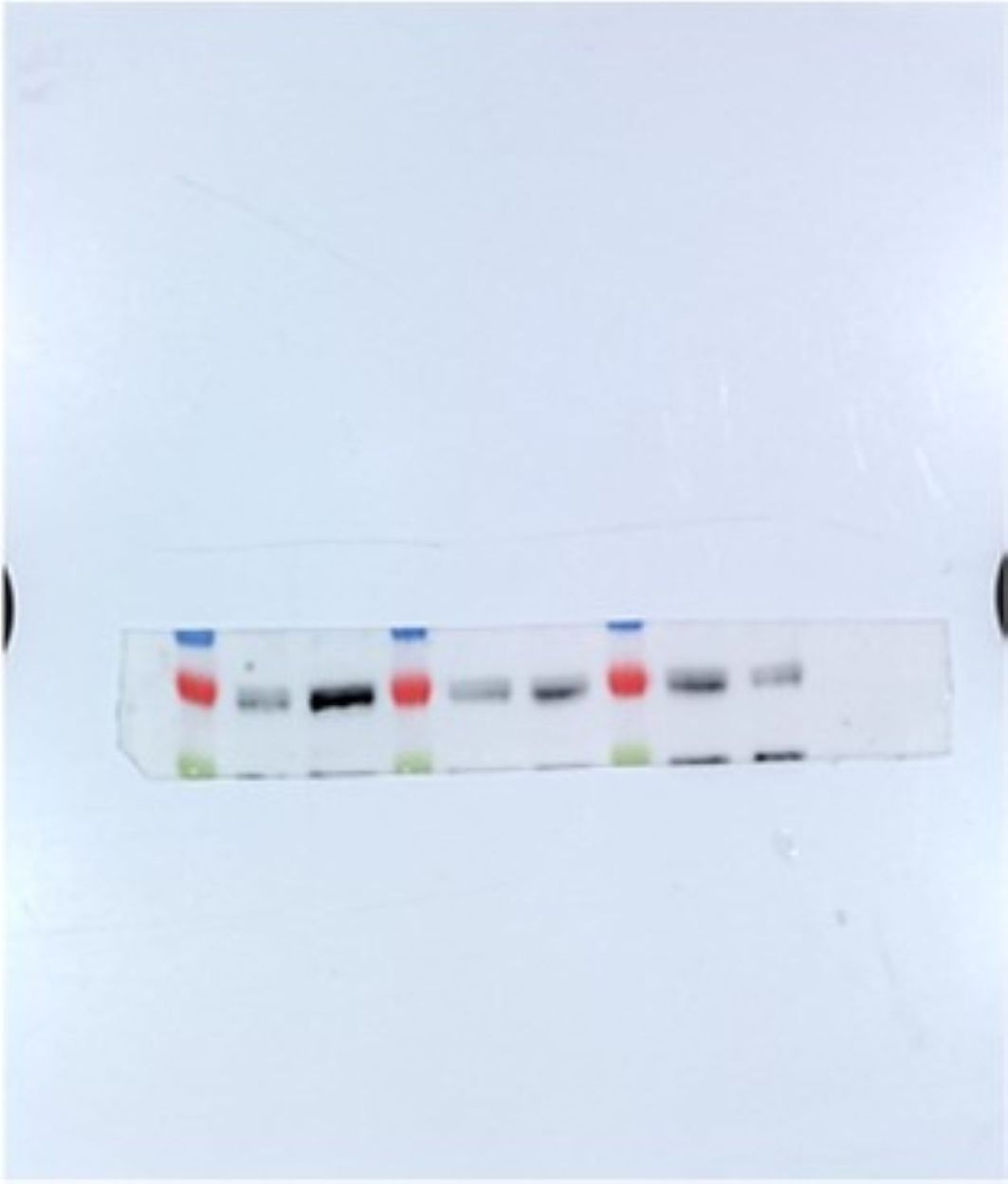

Supplement: Figure 9—figure supplement 1—source data 3. [file elife-104045-fig9-figsupp1-data3.zip › Figure 9-Figure supplement 1 Source data 3 (raw image of C)/HT1080 _MDAMB R17H INDUCIBLE TRF2 BLOT+marker.jpg]

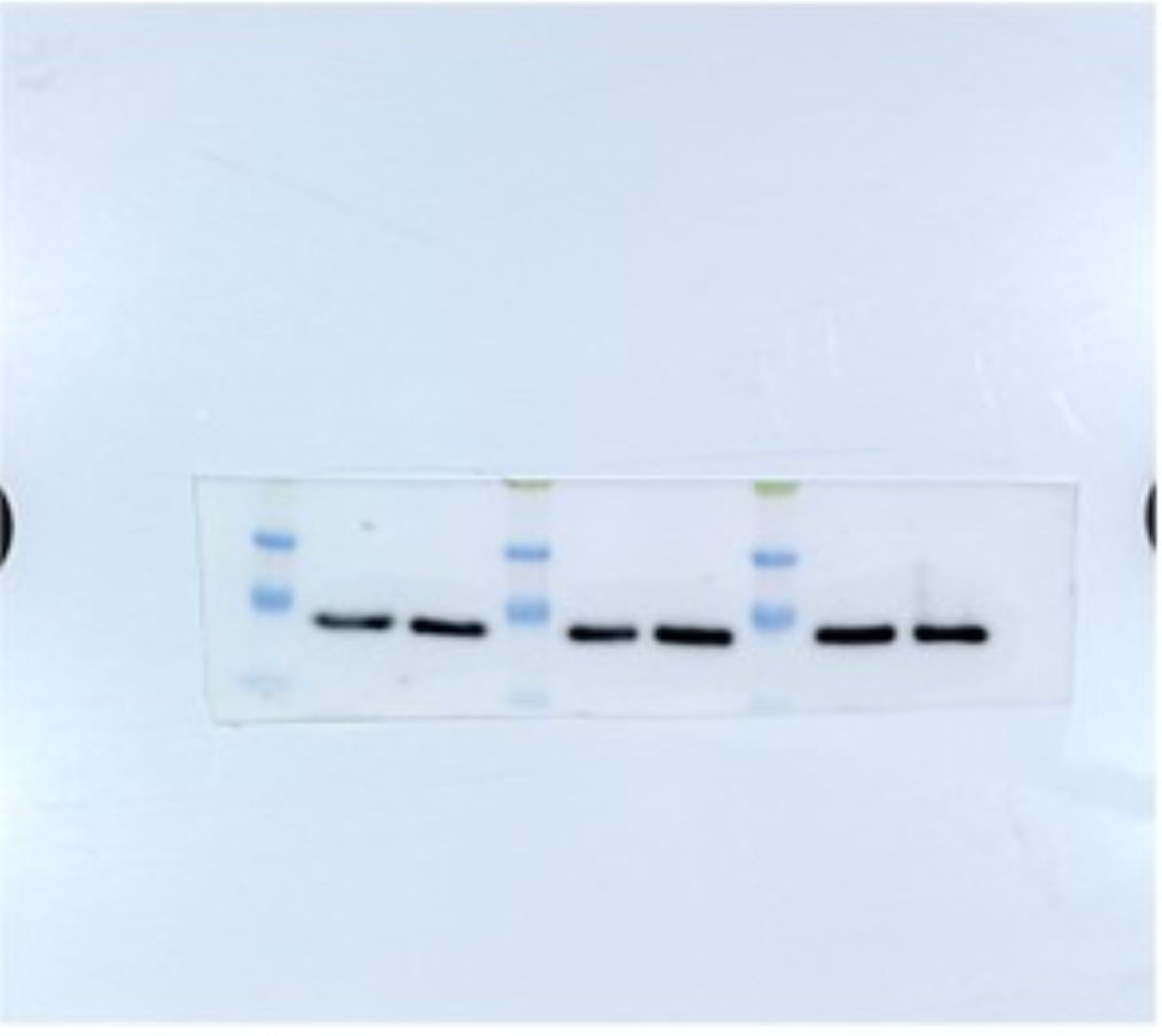

Supplement: Figure 9—figure supplement 1—source data 3. [file elife-104045-fig9-figsupp1-data3.zip › Figure 9-Figure supplement 1 Source data 3 (raw image of C)/HT1080 _MDAMB wt INDUCIBLE GAPDH BLOT+marker.png.jpg]

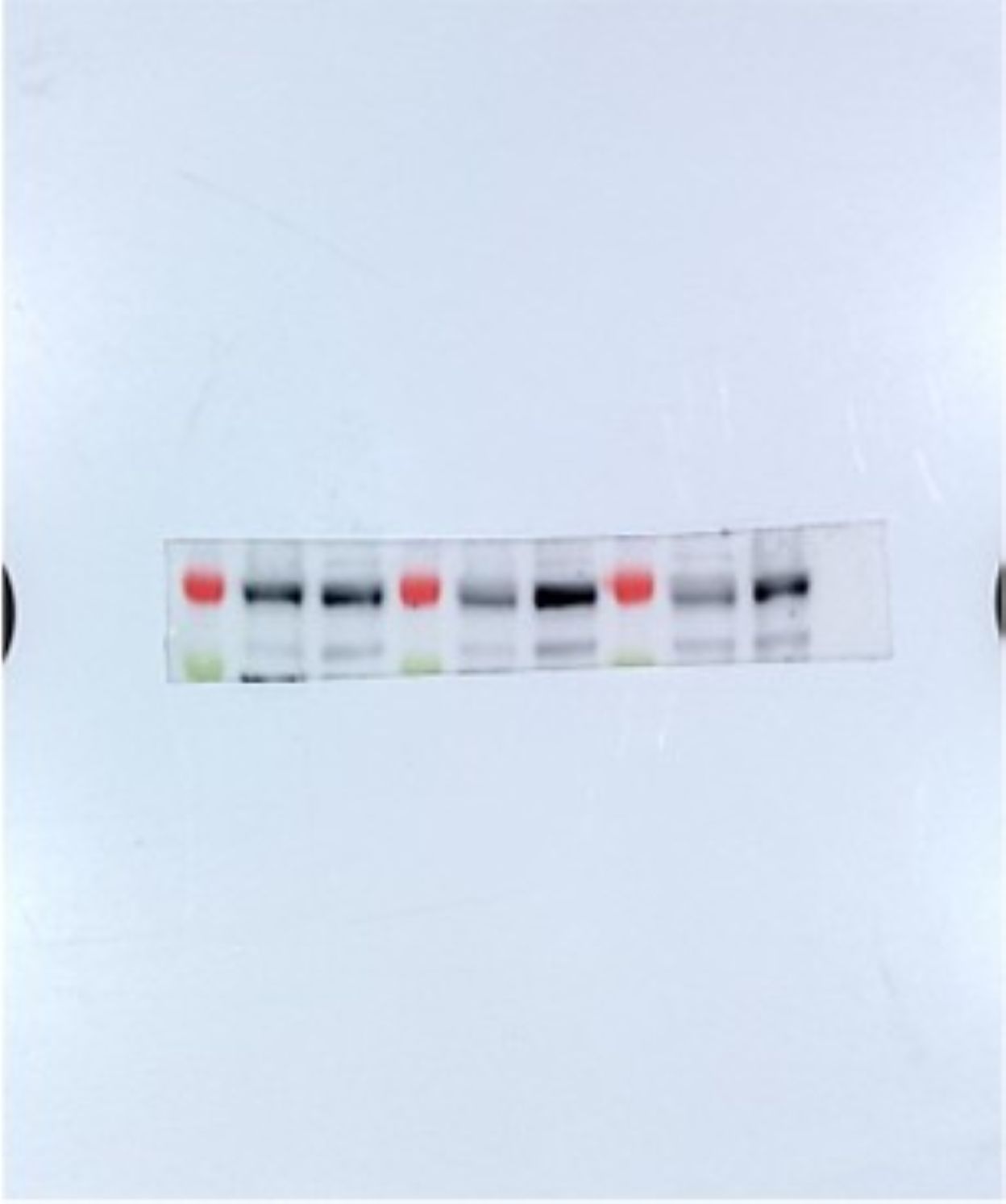

Supplement: Figure 9—figure supplement 1—source data 3. [file elife-104045-fig9-figsupp1-data3.zip › Figure 9-Figure supplement 1 Source data 3 (raw image of C)/HT1080 _MDAMB wt INDUCIBLE TRF2 BLOT+marker.png.jpg]

Supplementary Figure 1E Uncropped images with labelling

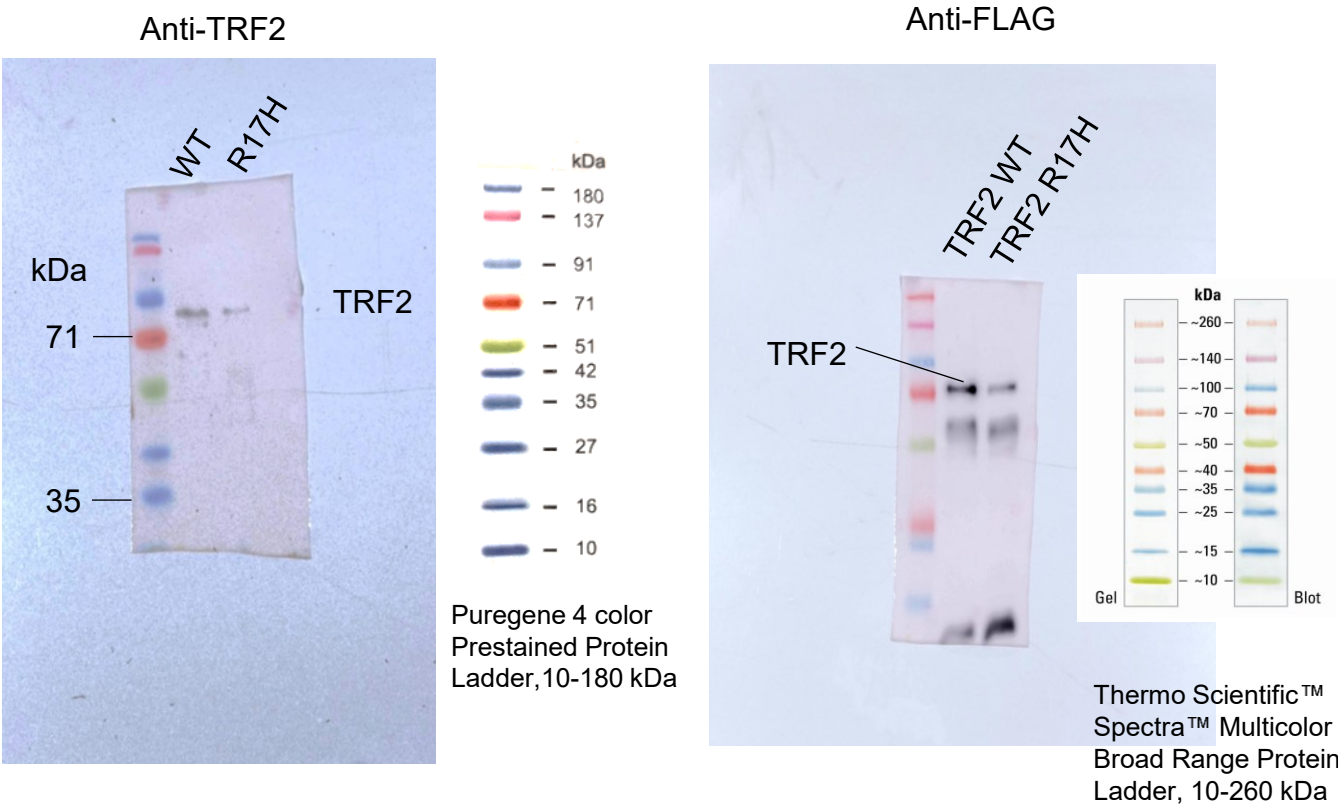

F

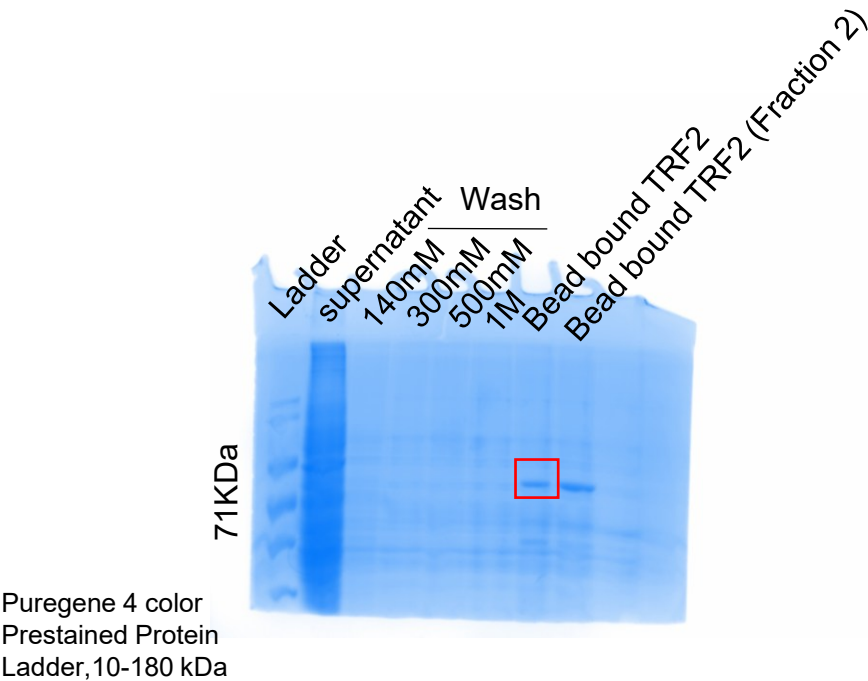

Supplement: Figure 9—figure supplement 1—source data 4. [file elife-104045-fig9-figsupp1-data4.pdf]

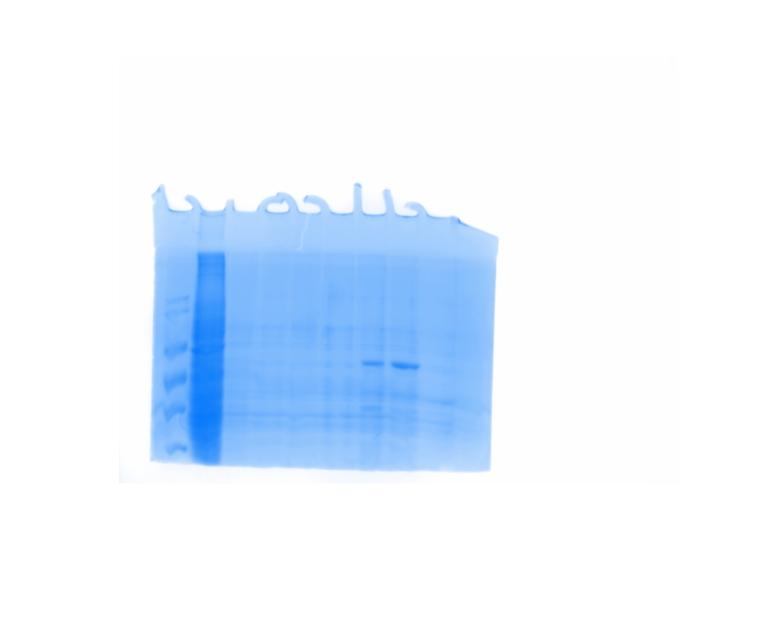

Supplement: Figure 9—figure supplement 1—source data 5. [file elife-104045-fig9-figsupp1-data5.zip › Figure 9-Figure supplement 1 Source data 5 (raw image of E)/CBB TRF2 purification gel.tif]

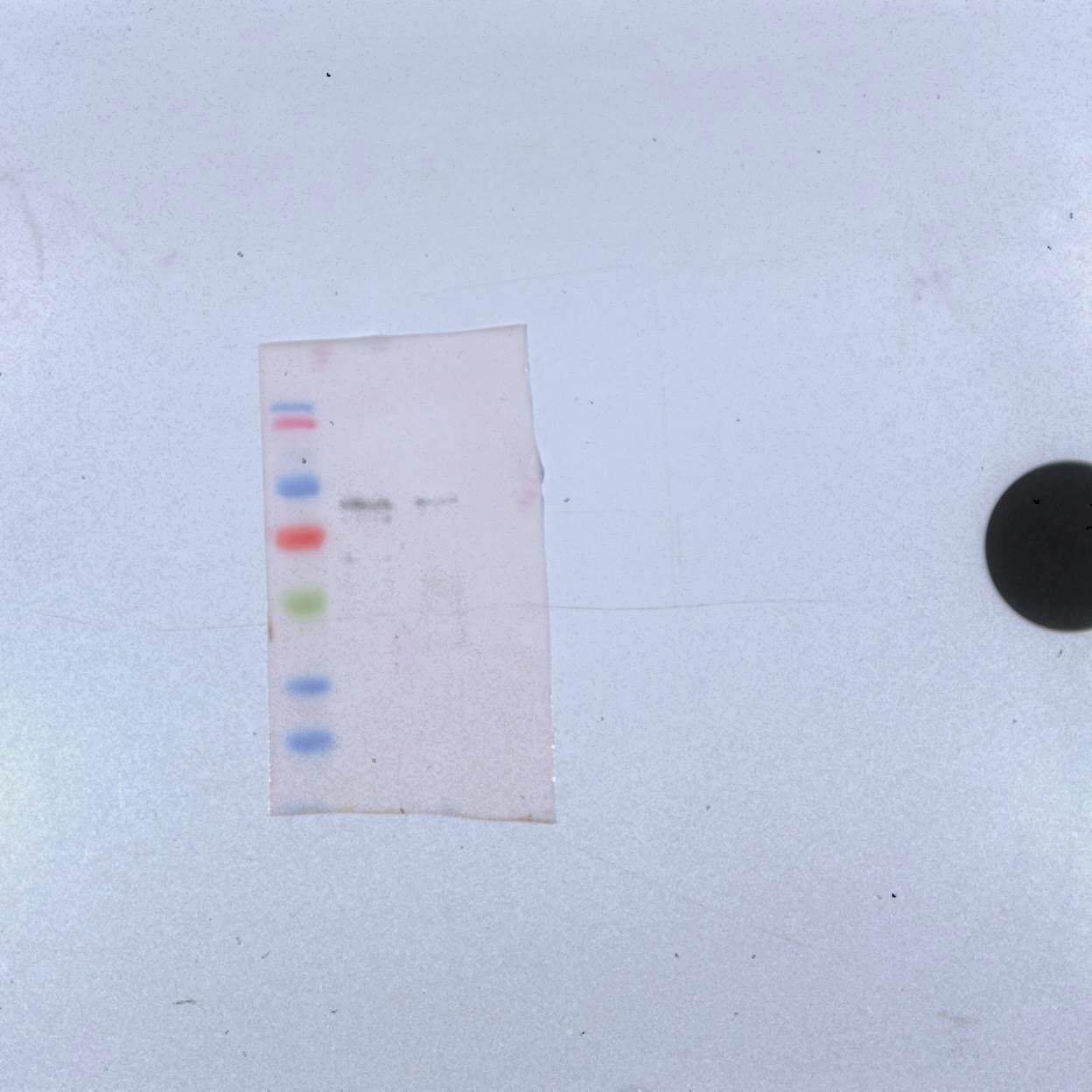

Supplement: Figure 9—figure supplement 1—source data 5. [file elife-104045-fig9-figsupp1-data5.zip › Figure 9-Figure supplement 1 Source data 5 (raw image of E)/r17h puri 20220920_125355_Ch+Marker.jpg]

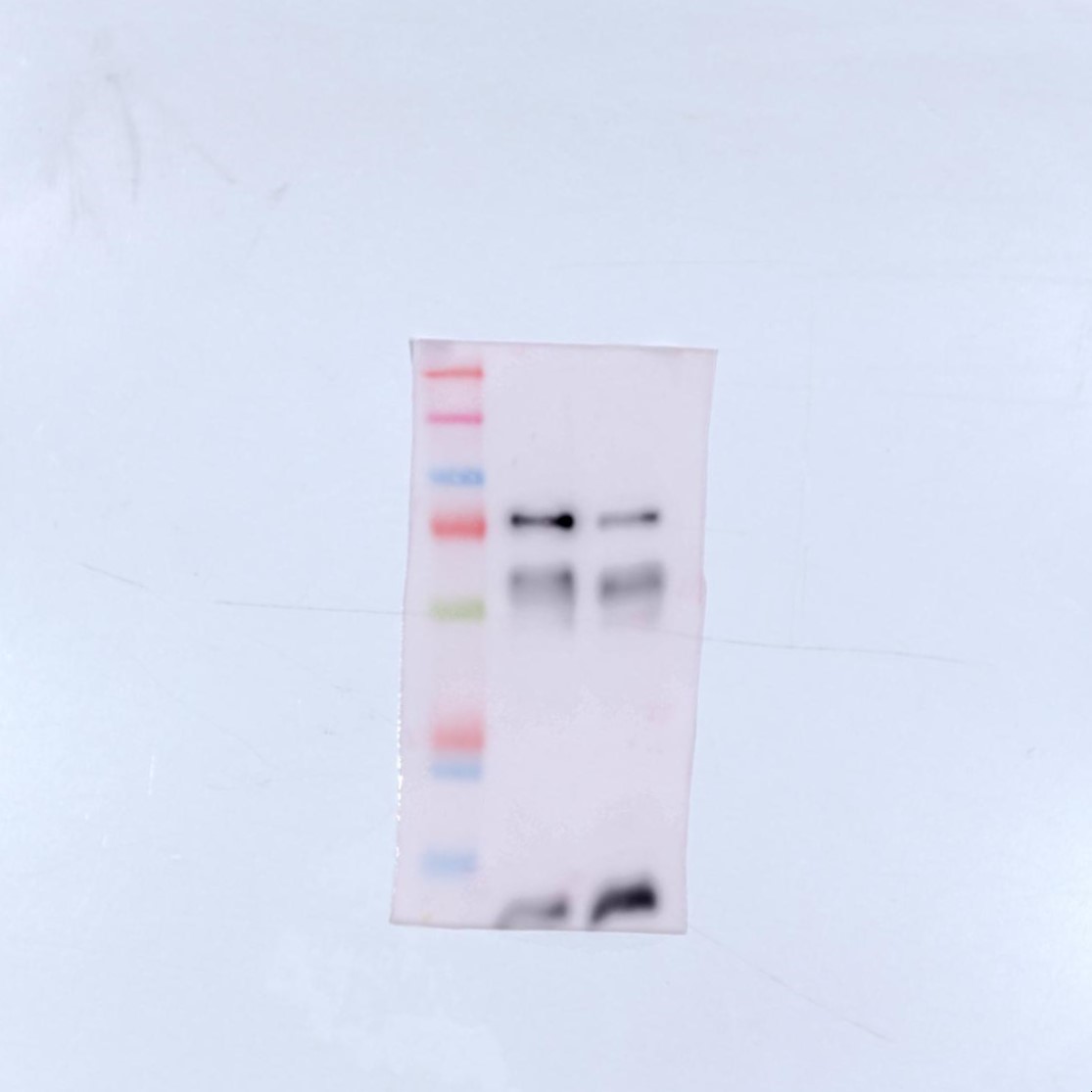

Supplement: Figure 9—figure supplement 1—source data 5. [file elife-104045-fig9-figsupp1-data5.zip › Figure 9-Figure supplement 1 Source data 5 (raw image of E)/trf2 puri 20220913_210720_Ch+Marker.jpg]

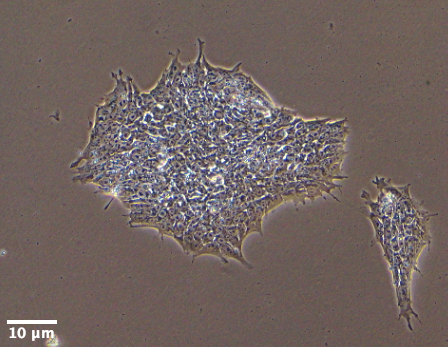

Supplement: Figure 10—source data 2. [file elife-104045-fig10-data2.zip › Figure 10B Source data iPSC IF/iPSC bright field image.tif]

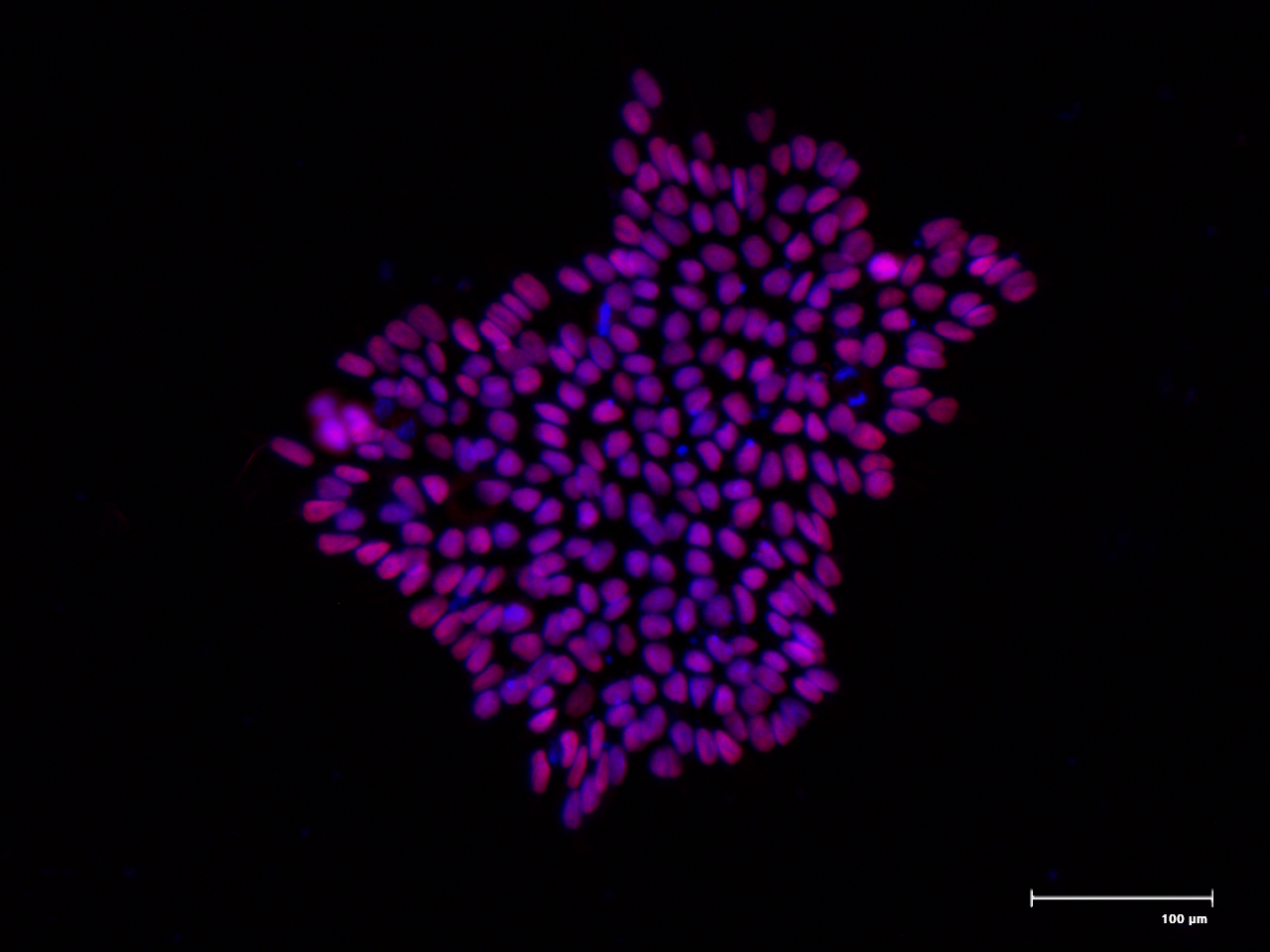

Supplement: Figure 10—source data 2. [file elife-104045-fig10-data2.zip › Figure 10B Source data iPSC IF/iPSC IF/Composite.tif]

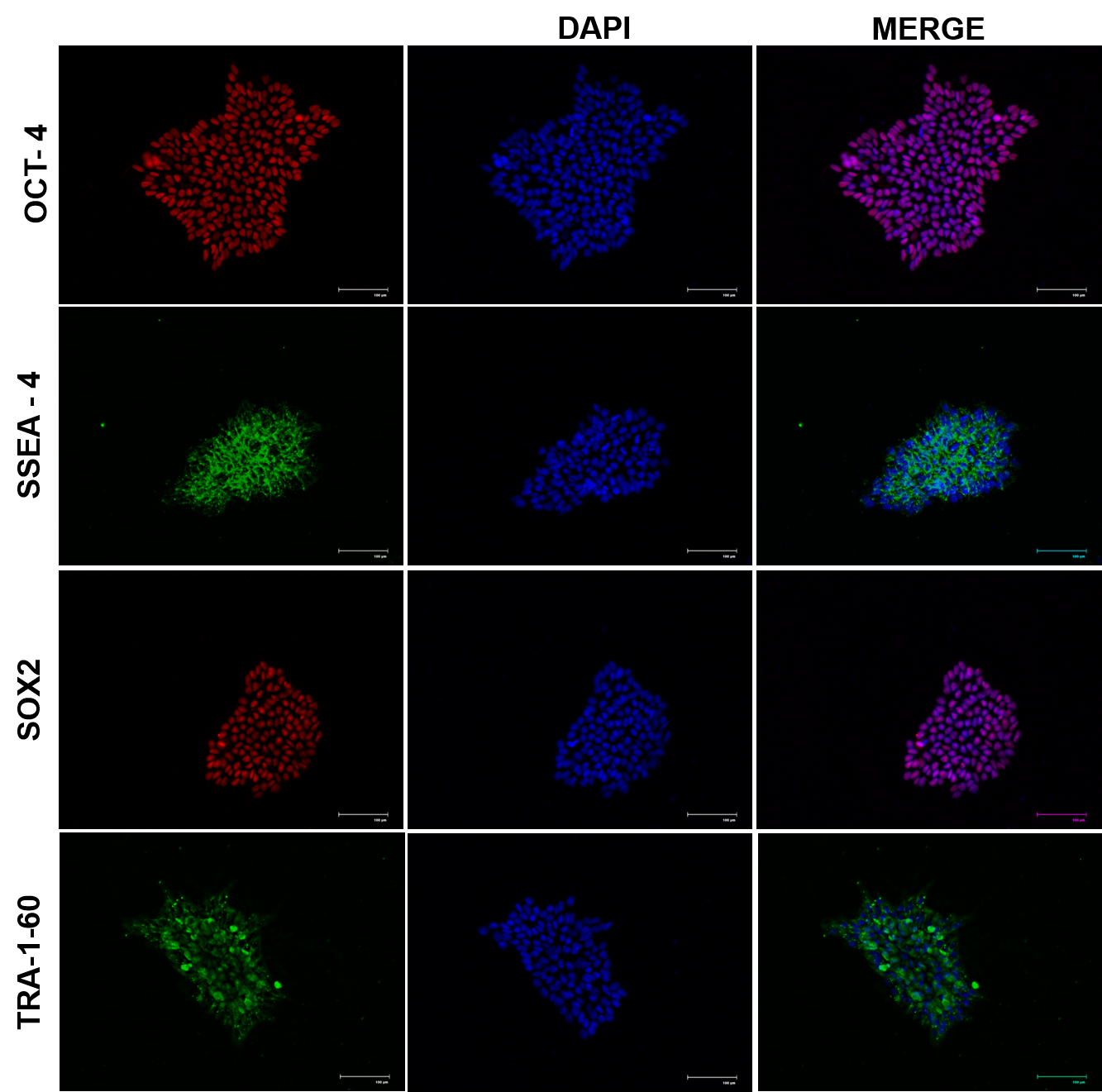

Supplement: Figure 10—source data 2. [file elife-104045-fig10-data2.zip › Figure 10B Source data iPSC IF/iPSC IF/IF.tif]

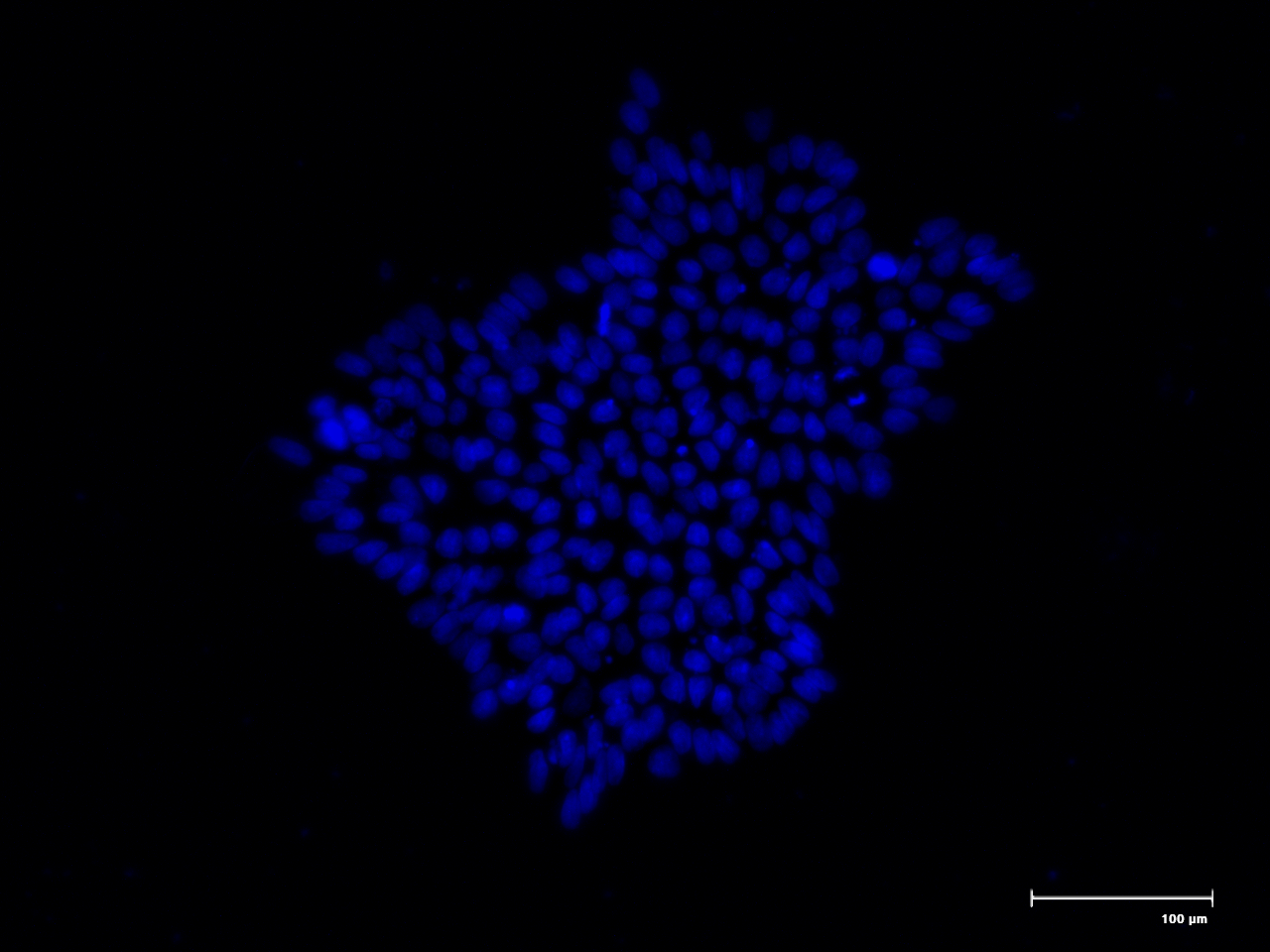

Supplement: Figure 10—source data 2. [file elife-104045-fig10-data2.zip › Figure 10B Source data iPSC IF/iPSC IF/oct2b.tif]

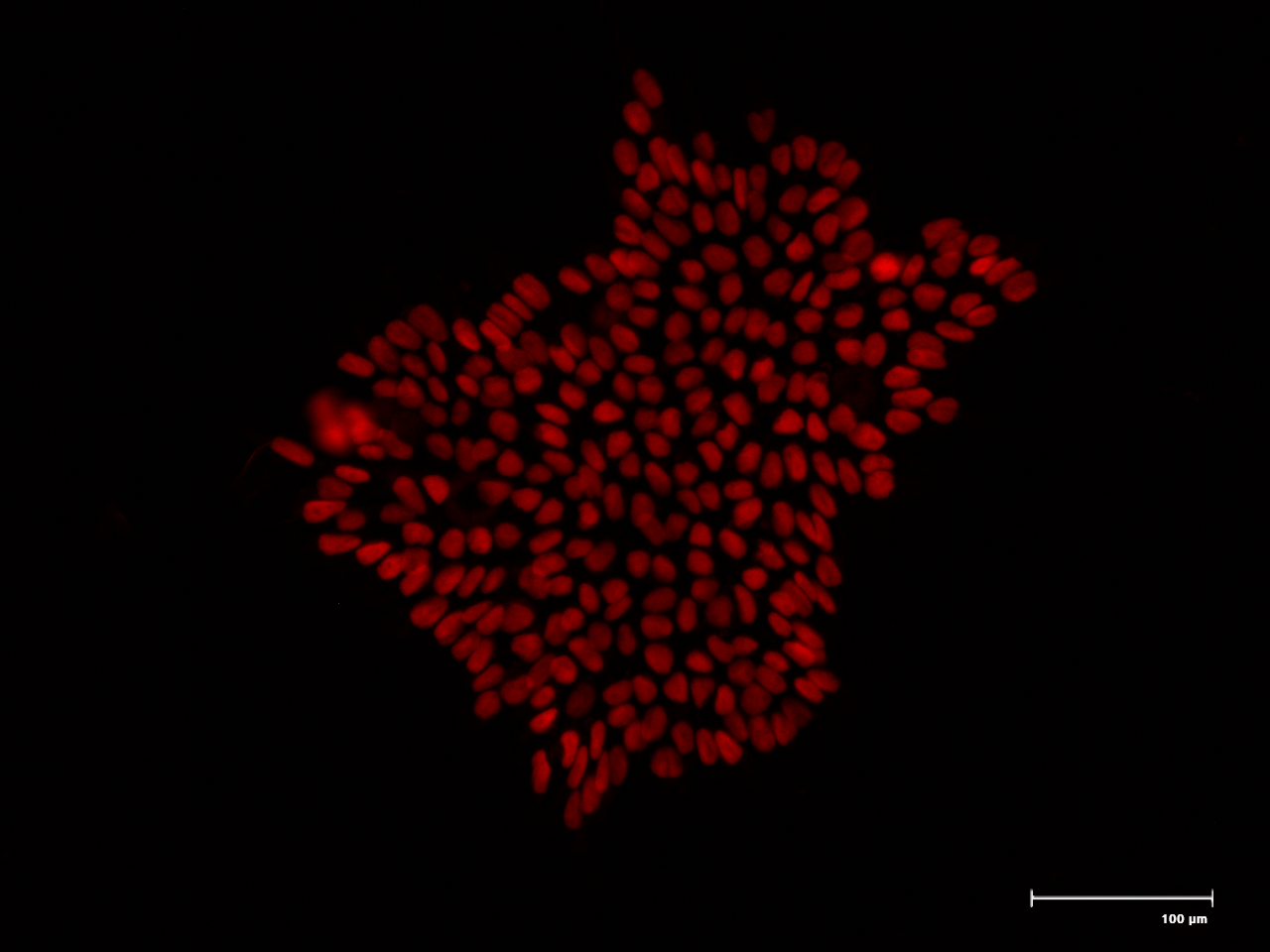

Supplement: Figure 10—source data 2. [file elife-104045-fig10-data2.zip › Figure 10B Source data iPSC IF/iPSC IF/oct2r.tif]

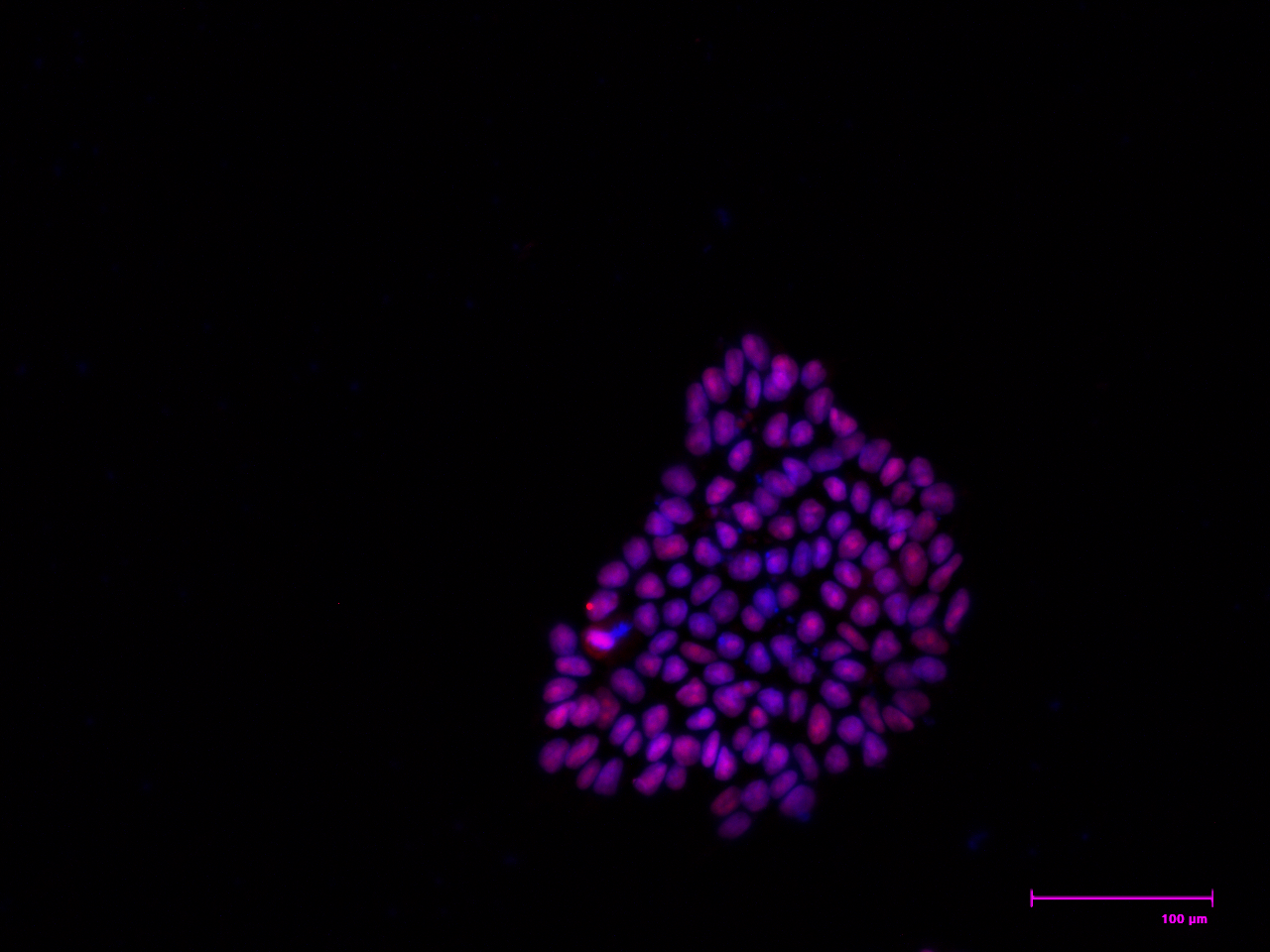

Supplement: Figure 10—source data 2. [file elife-104045-fig10-data2.zip › Figure 10B Source data iPSC IF/iPSC IF/sox2.tif]

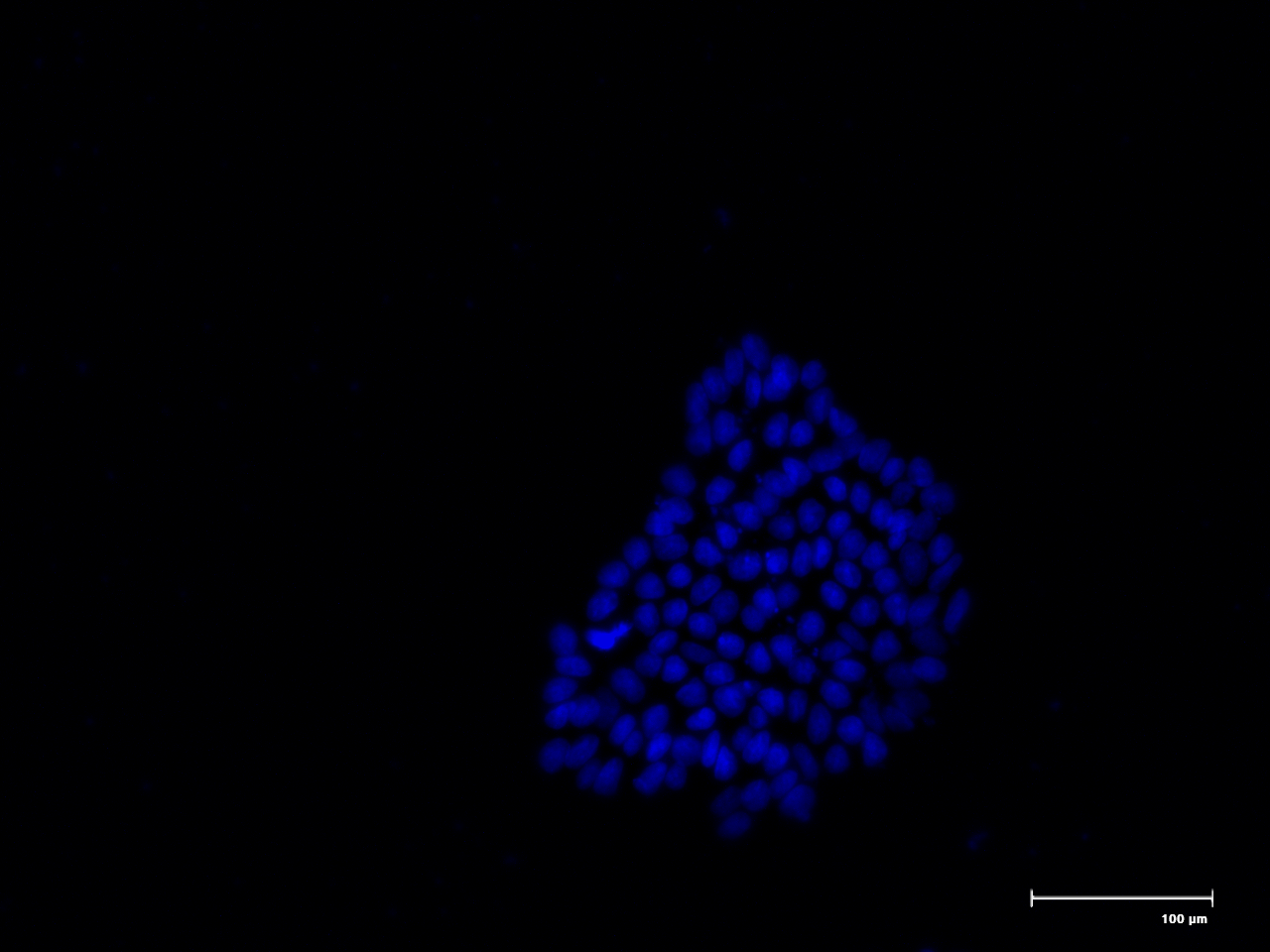

Supplement: Figure 10—source data 2. [file elife-104045-fig10-data2.zip › Figure 10B Source data iPSC IF/iPSC IF/soxb1.tif]

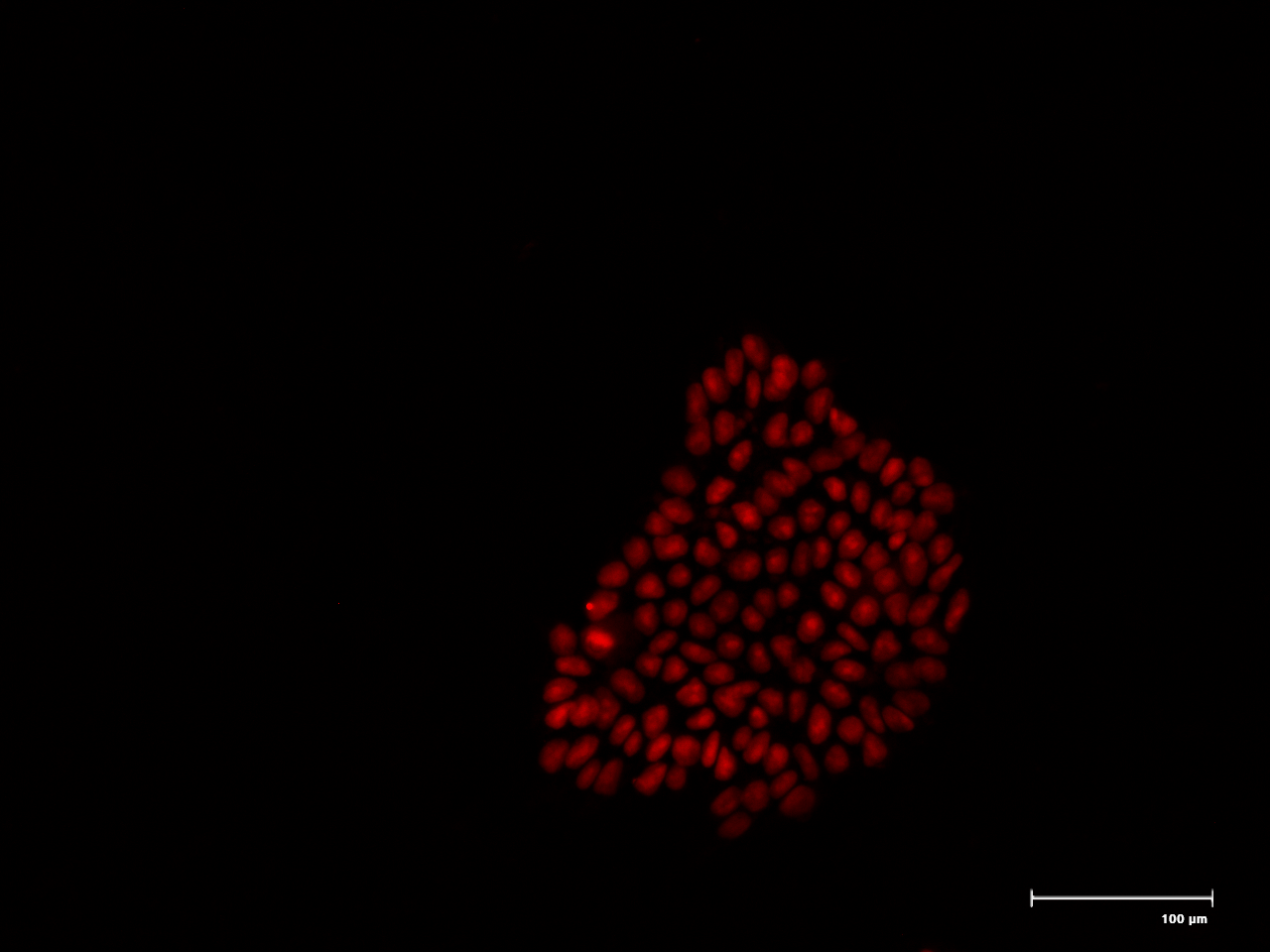

Supplement: Figure 10—source data 2. [file elife-104045-fig10-data2.zip › Figure 10B Source data iPSC IF/iPSC IF/soxr1.tif]

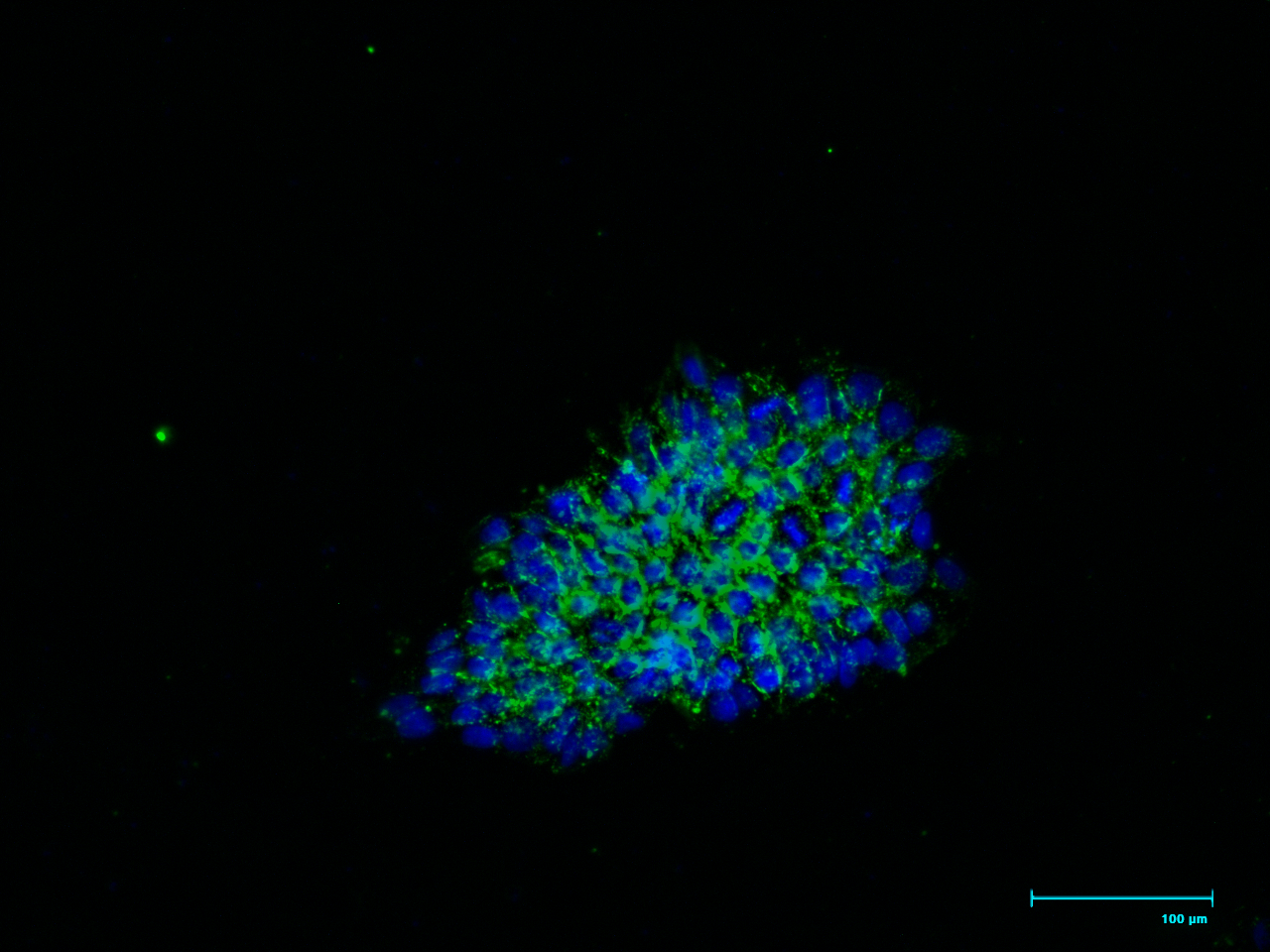

Supplement: Figure 10—source data 2. [file elife-104045-fig10-data2.zip › Figure 10B Source data iPSC IF/iPSC IF/sseacomposite.tif]

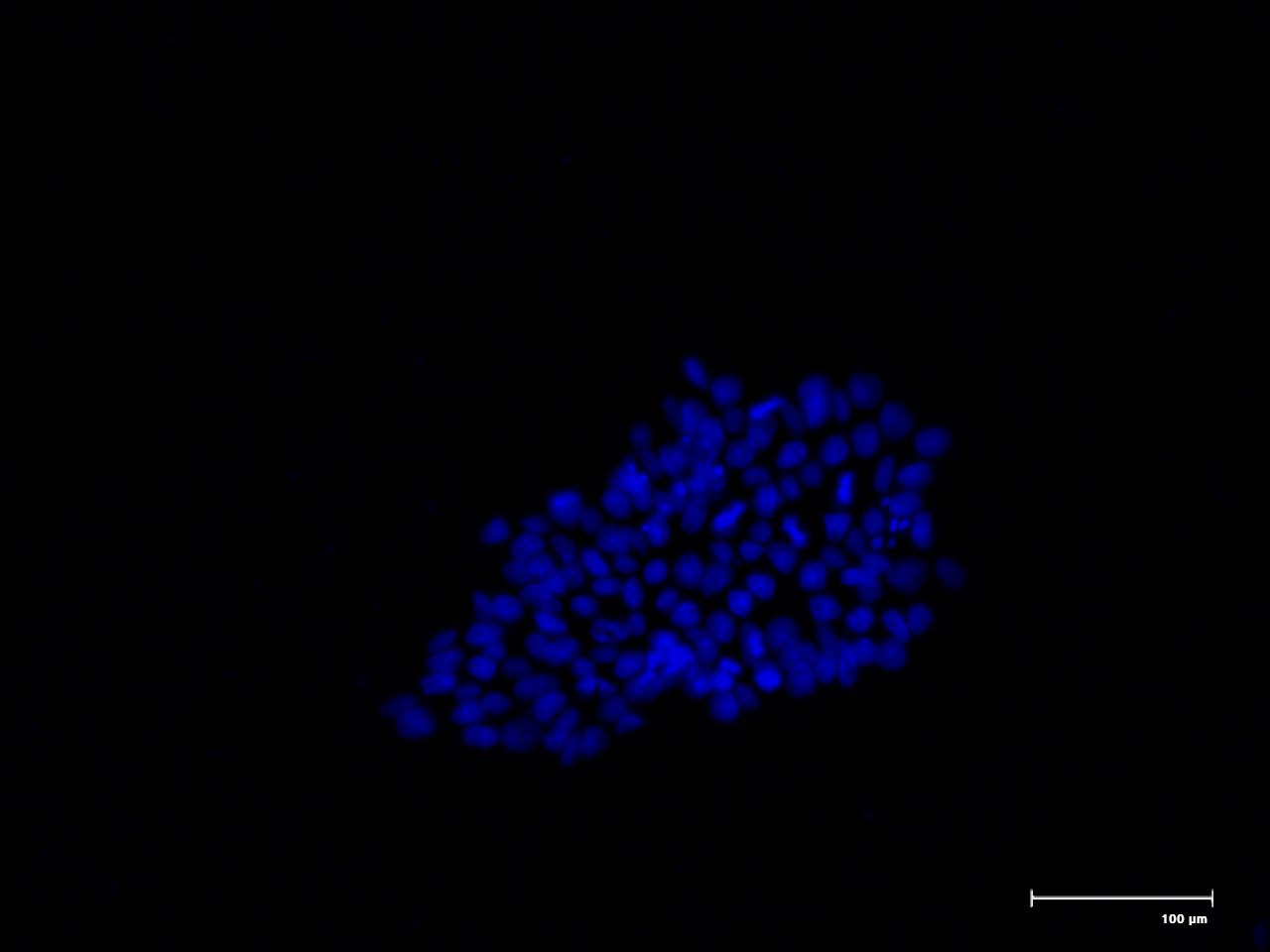

Supplement: Figure 10—source data 2. [file elife-104045-fig10-data2.zip › Figure 10B Source data iPSC IF/iPSC IF/sseag.tif]

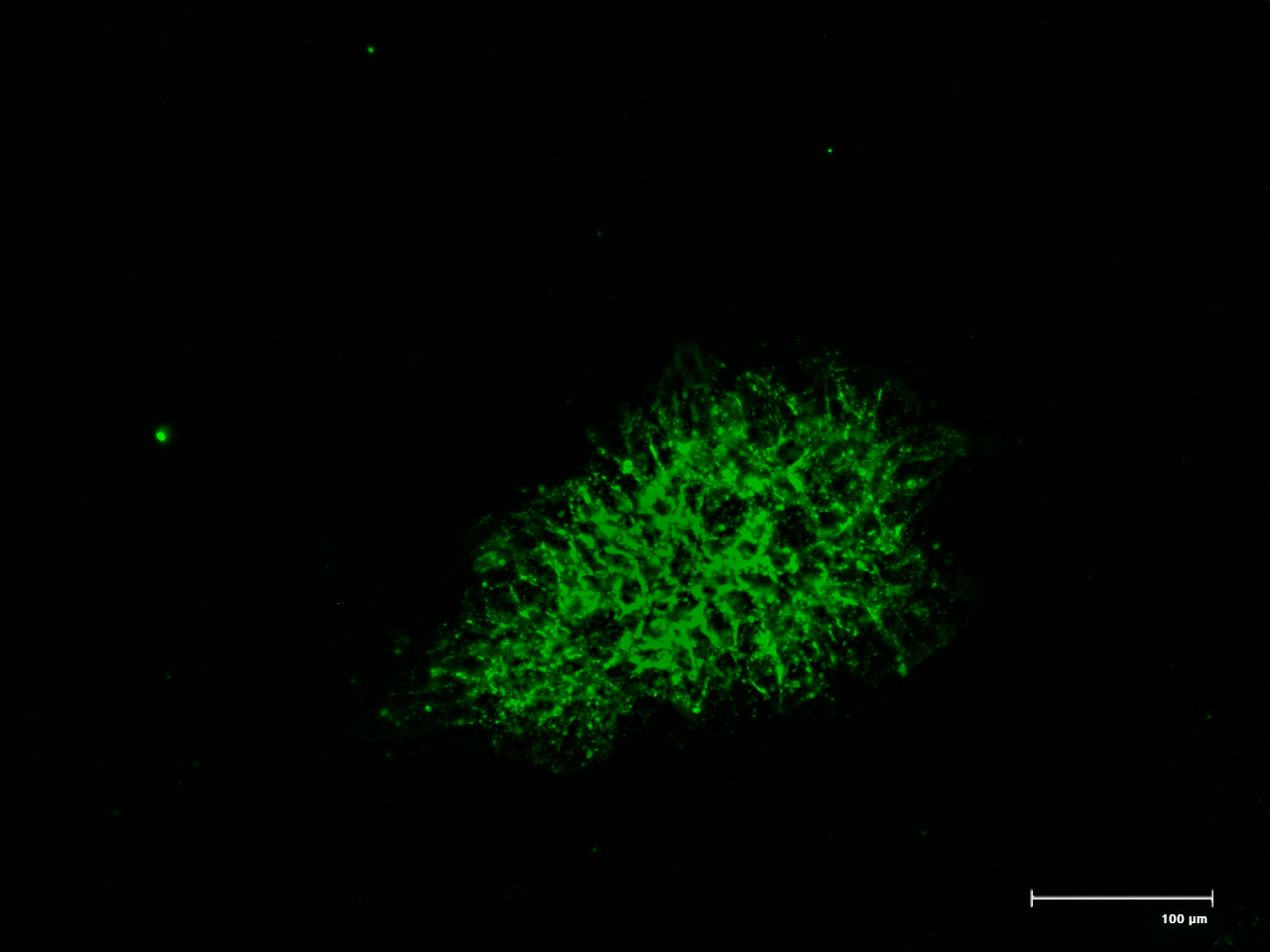

Supplement: Figure 10—source data 2. [file elife-104045-fig10-data2.zip › Figure 10B Source data iPSC IF/iPSC IF/sseagg.tif]

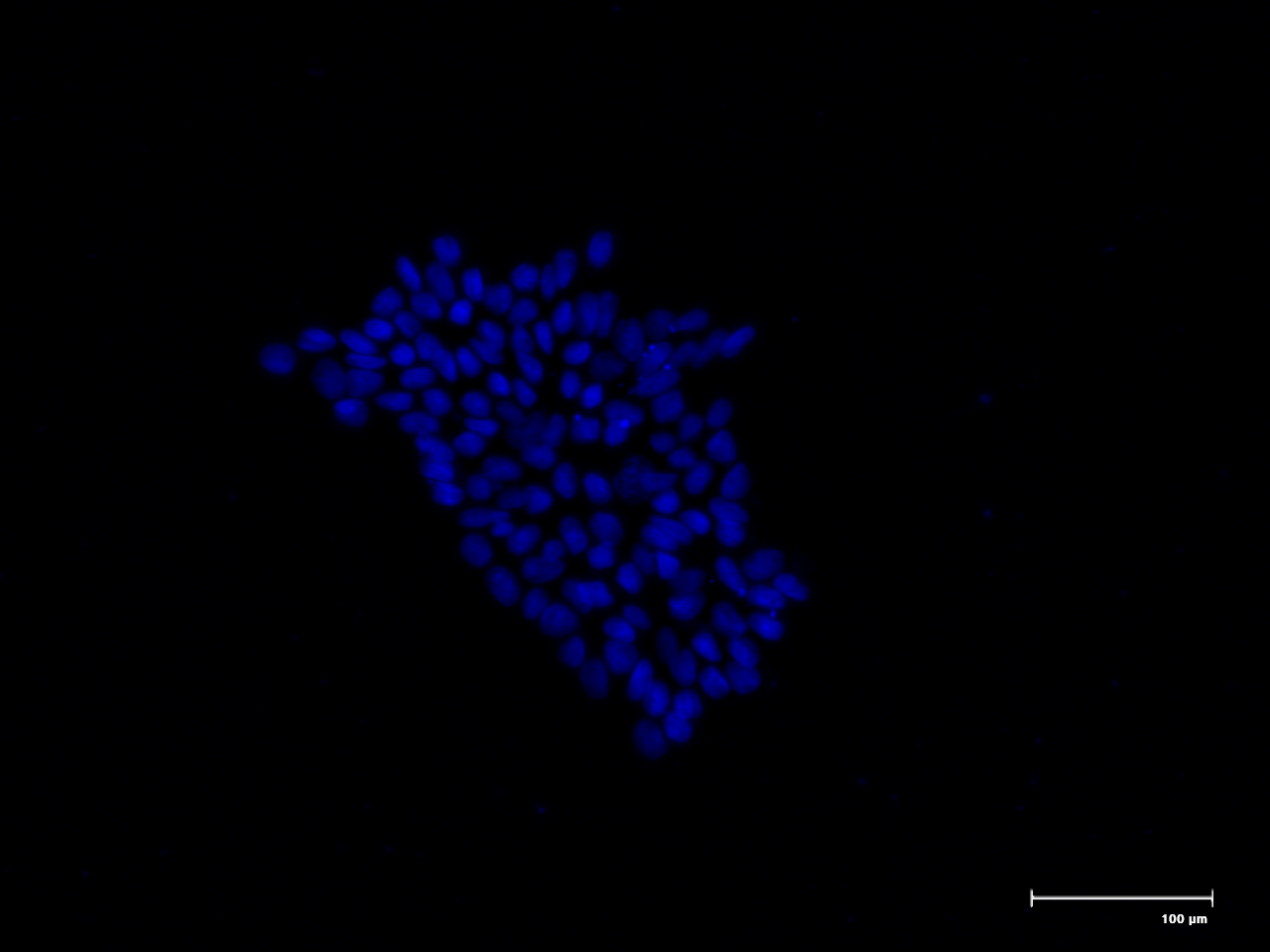

Supplement: Figure 10—source data 2. [file elife-104045-fig10-data2.zip › Figure 10B Source data iPSC IF/iPSC IF/TRAb.tif]

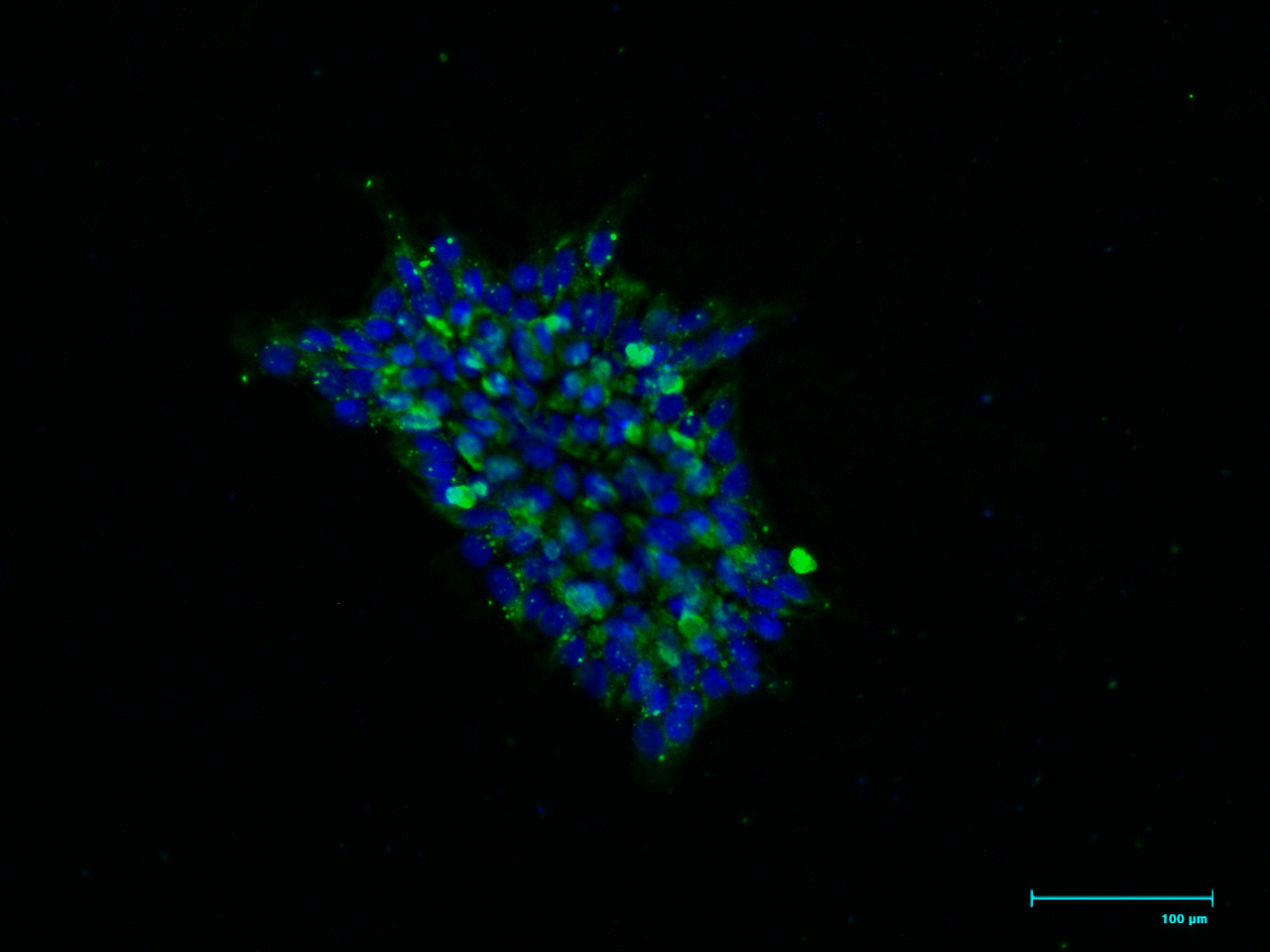

Supplement: Figure 10—source data 2. [file elife-104045-fig10-data2.zip › Figure 10B Source data iPSC IF/iPSC IF/TRAg.tif]

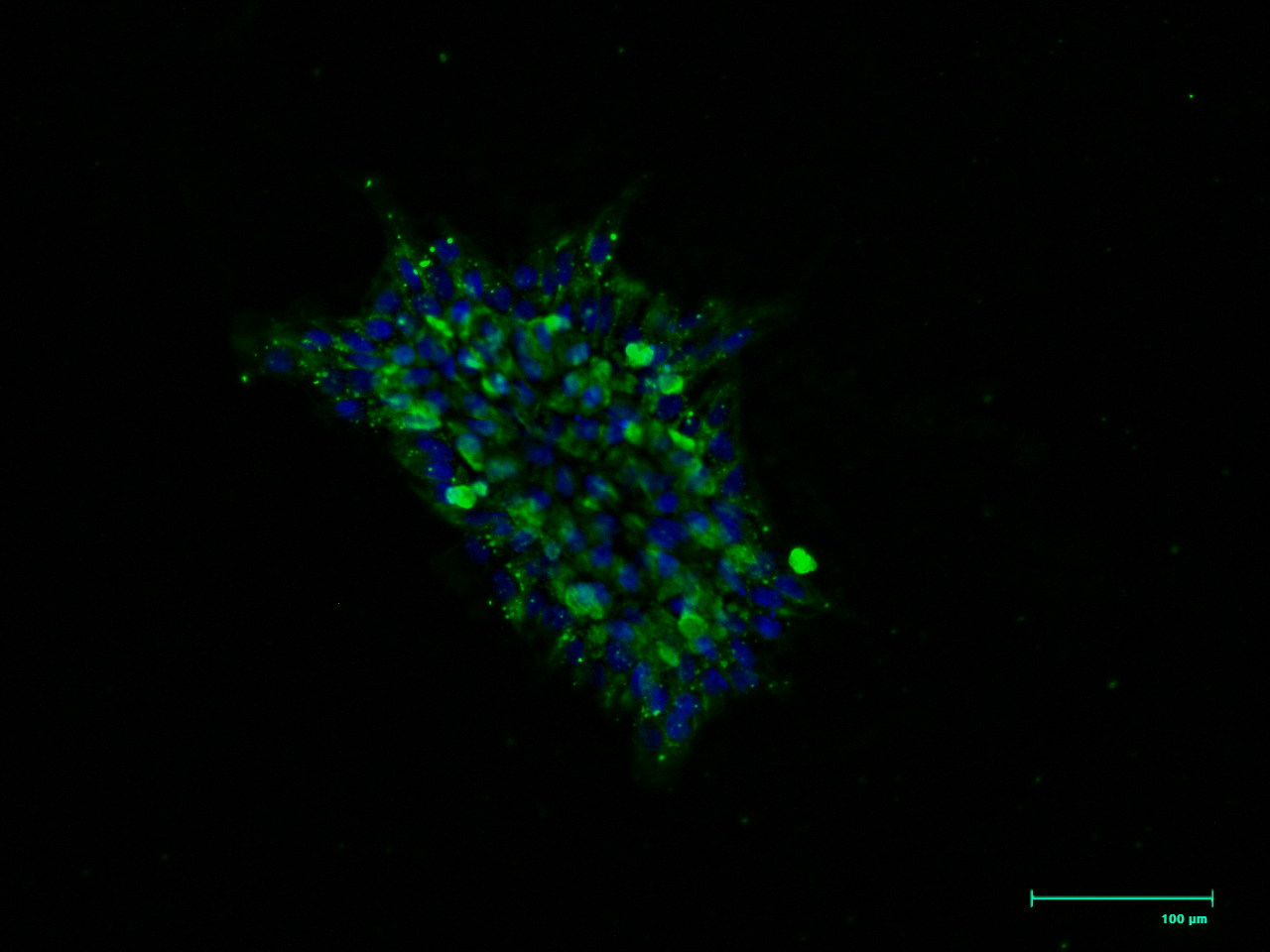

Supplement: Figure 10—source data 2. [file elife-104045-fig10-data2.zip › Figure 10B Source data iPSC IF/iPSC IF/tragc.tif]

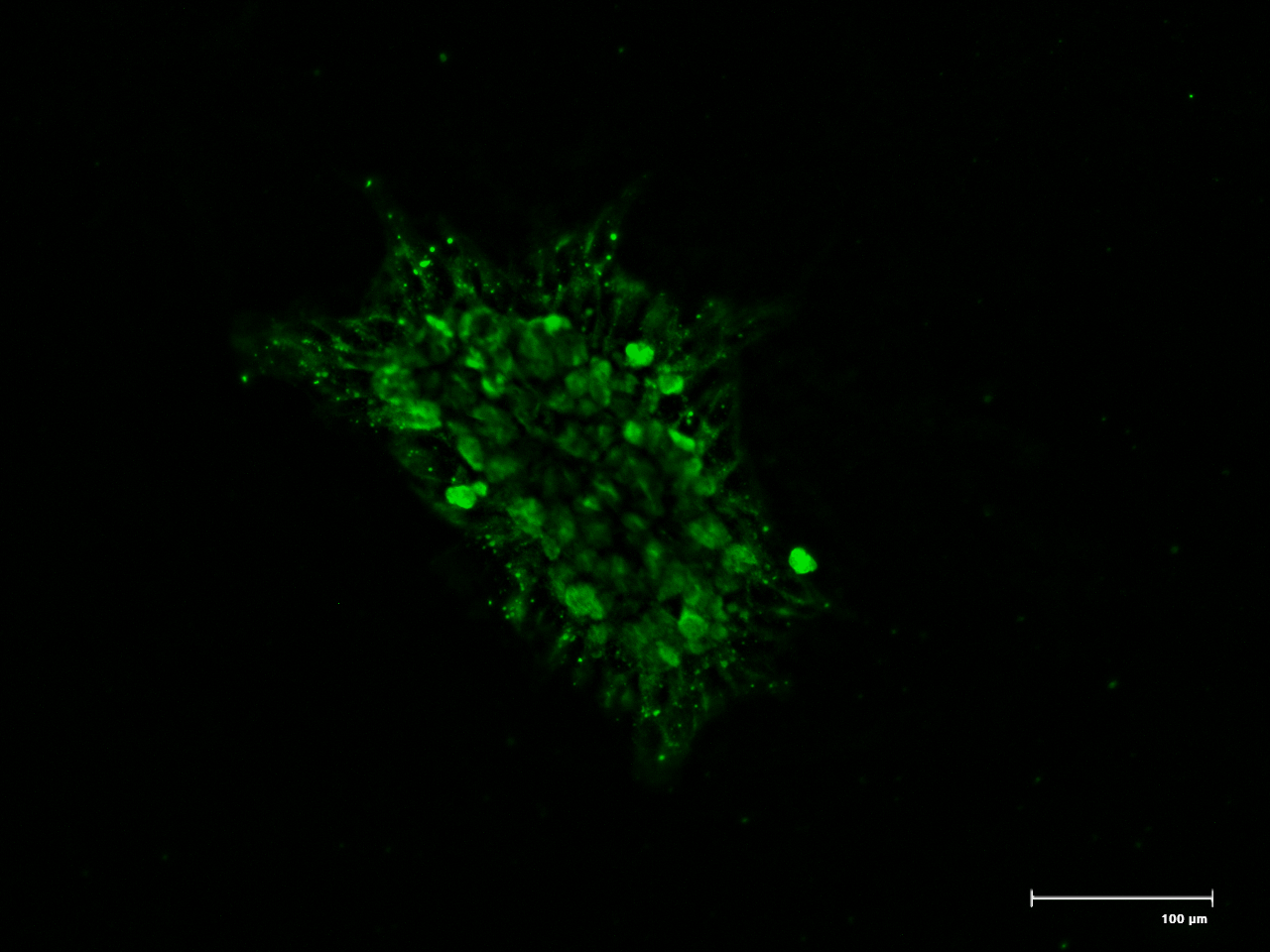

Supplement: Figure 10—source data 2. [file elife-104045-fig10-data2.zip › Figure 10B Source data iPSC IF/iPSC IF/tragg.tif]

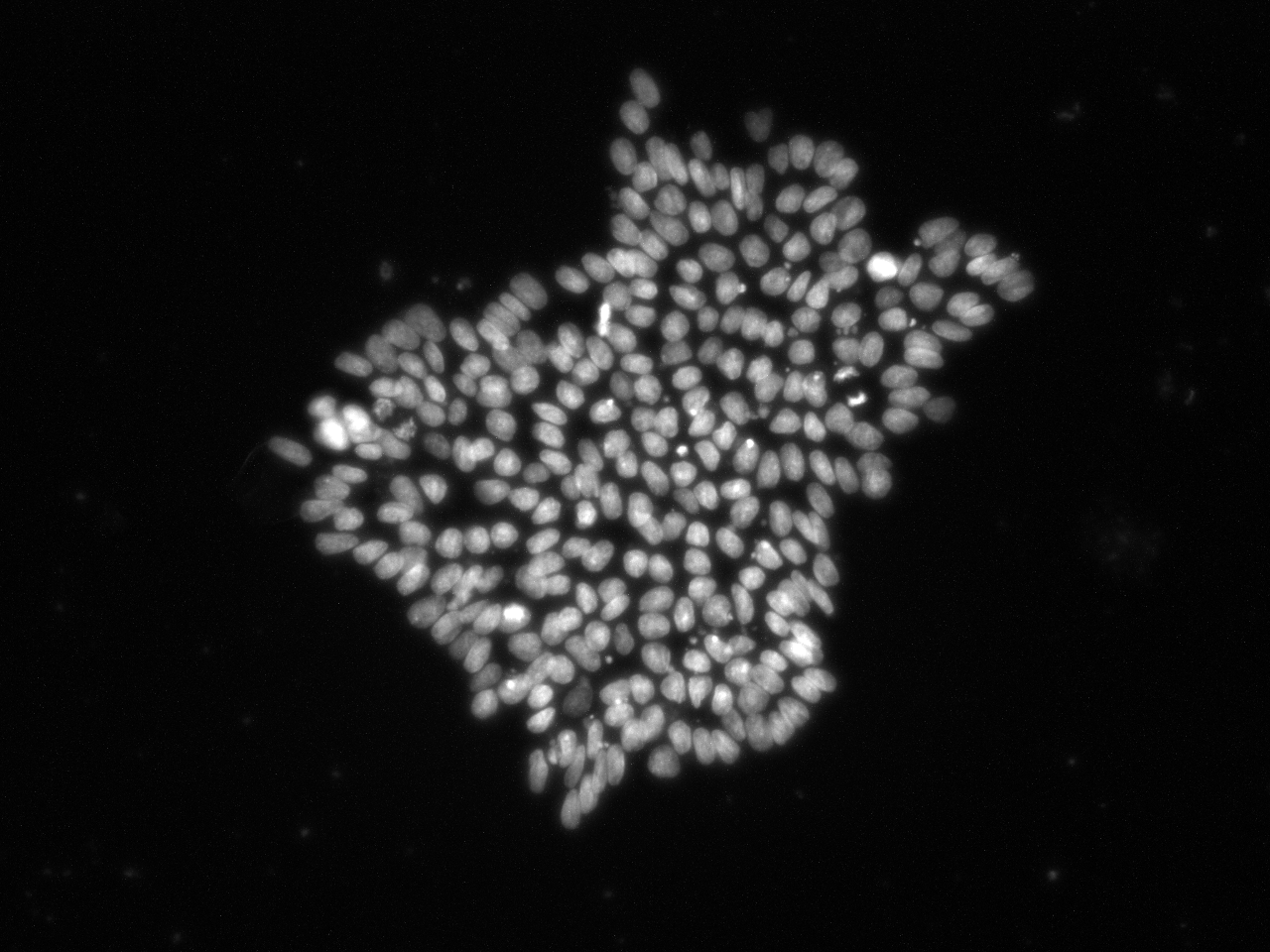

Supplement: Figure 10—source data 2. [file elife-104045-fig10-data2.zip › Figure 10B Source data iPSC IF/iPSC IF/unprocessed/oct2b - Blue Light.tif]

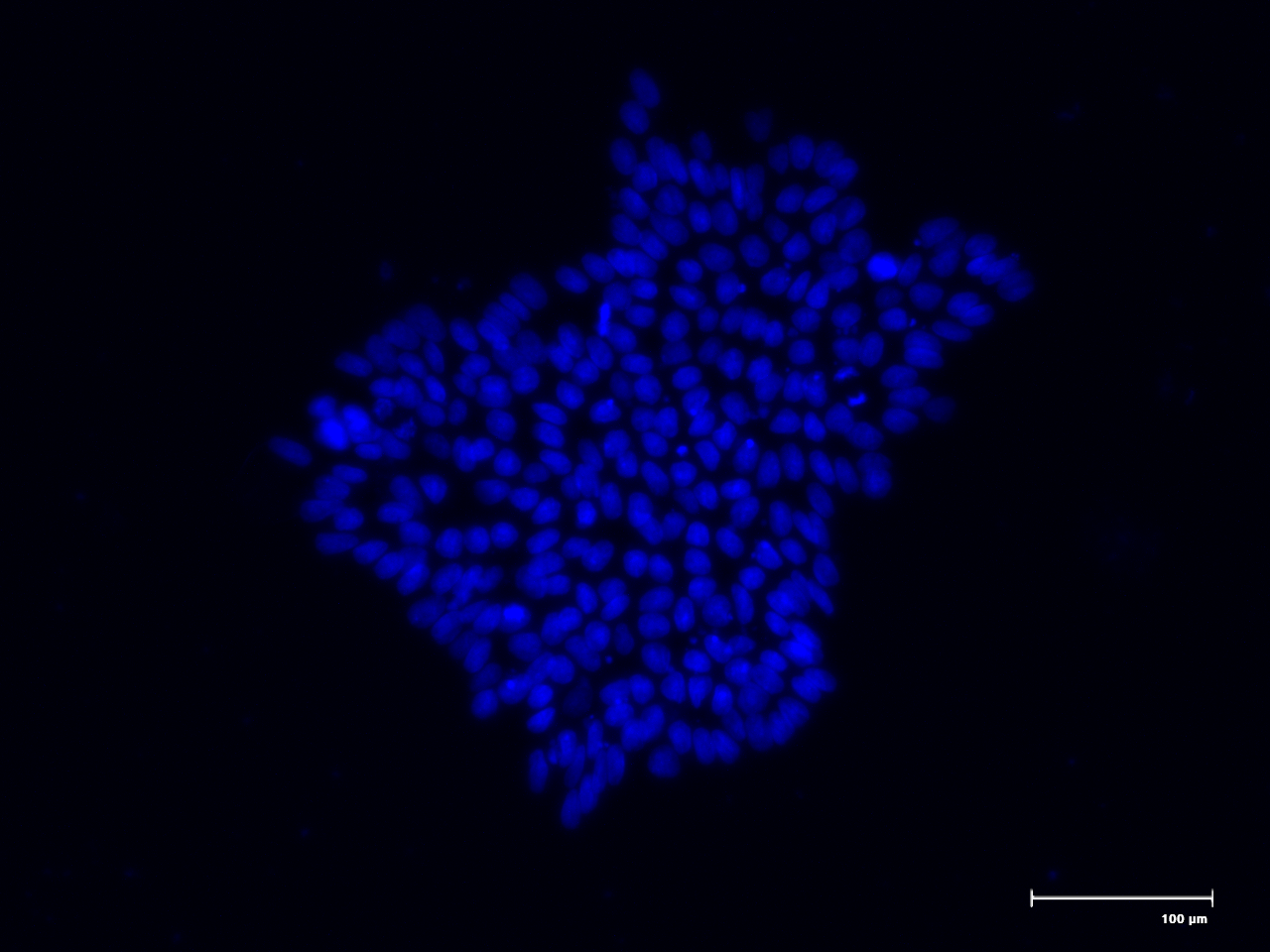

Supplement: Figure 10—source data 2. [file elife-104045-fig10-data2.zip › Figure 10B Source data iPSC IF/iPSC IF/unprocessed/oct2b.tif]

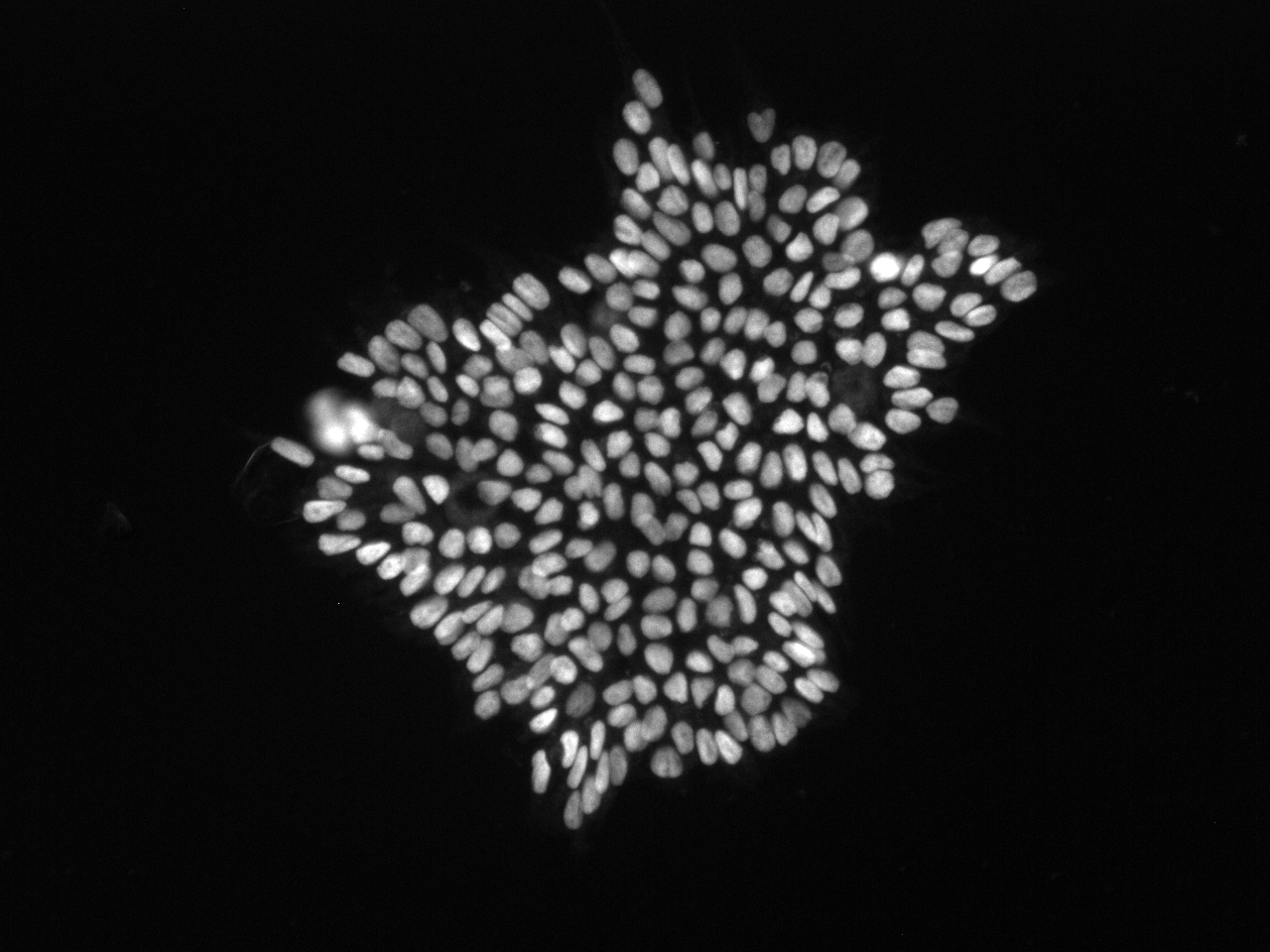

Supplement: Figure 10—source data 2. [file elife-104045-fig10-data2.zip › Figure 10B Source data iPSC IF/iPSC IF/unprocessed/oct2r - Red Light.tif]

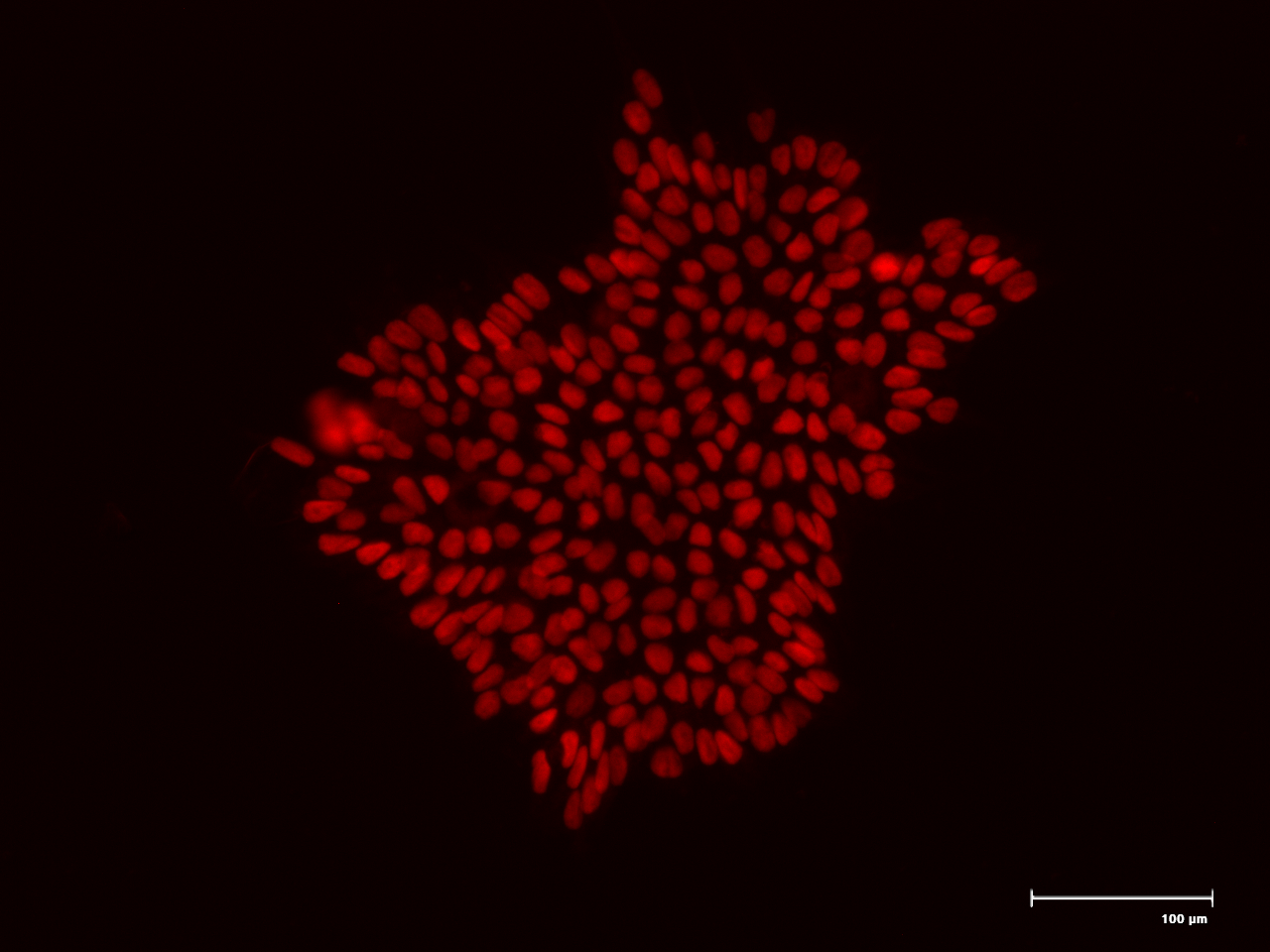

Supplement: Figure 10—source data 2. [file elife-104045-fig10-data2.zip › Figure 10B Source data iPSC IF/iPSC IF/unprocessed/oct2r.tif]

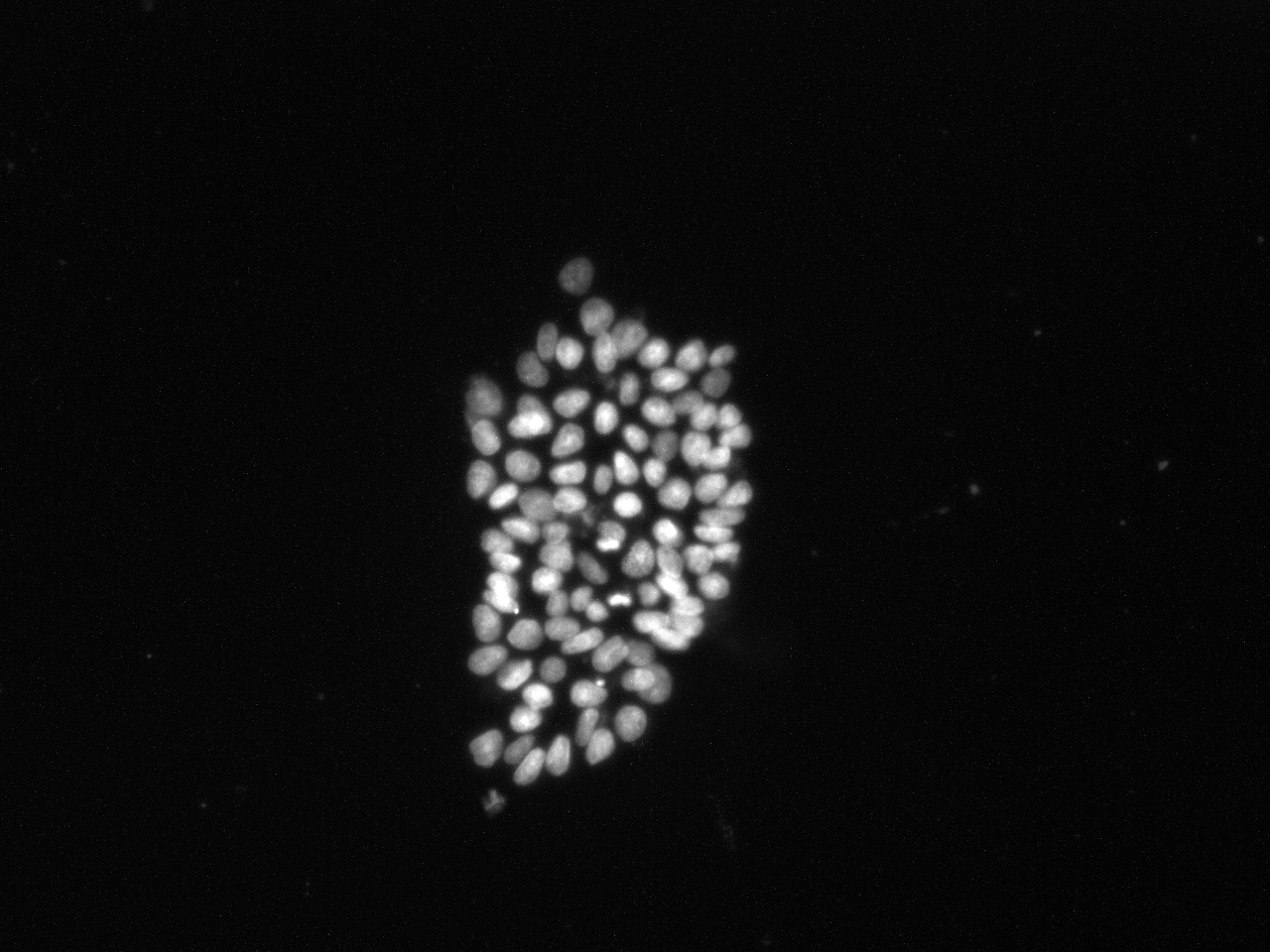

Supplement: Figure 10—source data 2. [file elife-104045-fig10-data2.zip › Figure 10B Source data iPSC IF/iPSC IF/unprocessed/oct4b - Blue Light.tif]

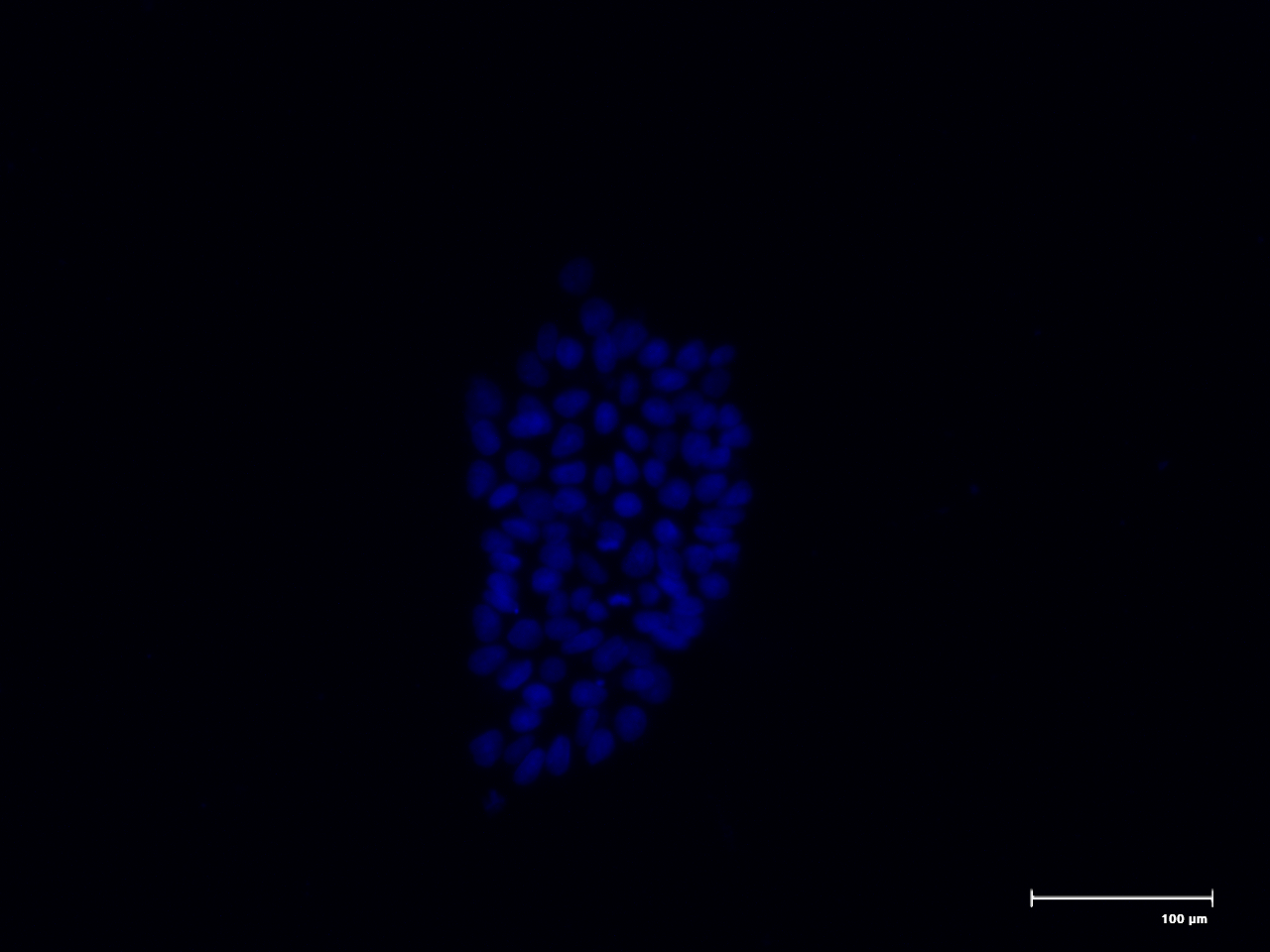

Supplement: Figure 10—source data 2. [file elife-104045-fig10-data2.zip › Figure 10B Source data iPSC IF/iPSC IF/unprocessed/oct4b.tif]

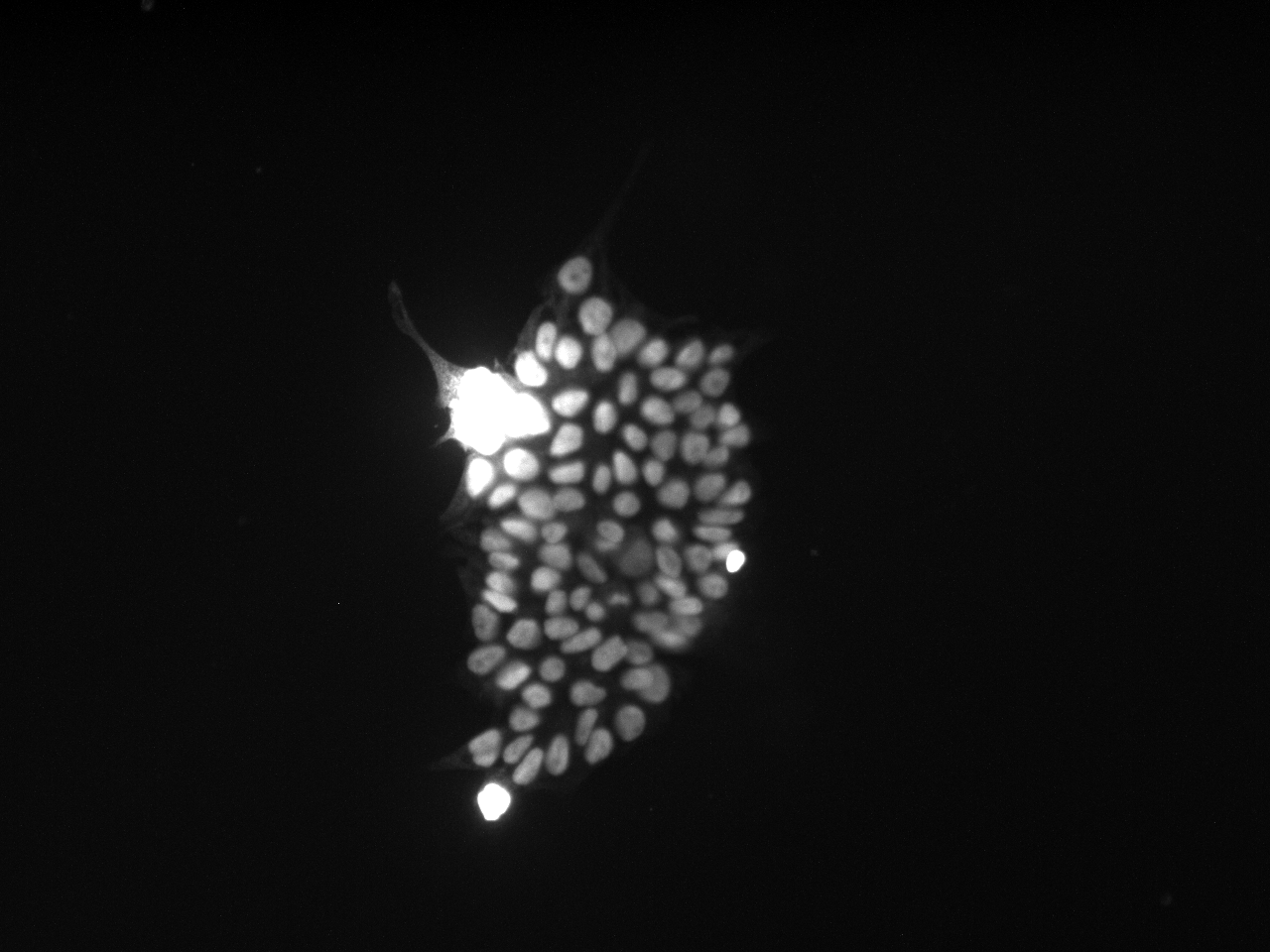

Supplement: Figure 10—source data 2. [file elife-104045-fig10-data2.zip › Figure 10B Source data iPSC IF/iPSC IF/unprocessed/oct4r - Red Light.tif]

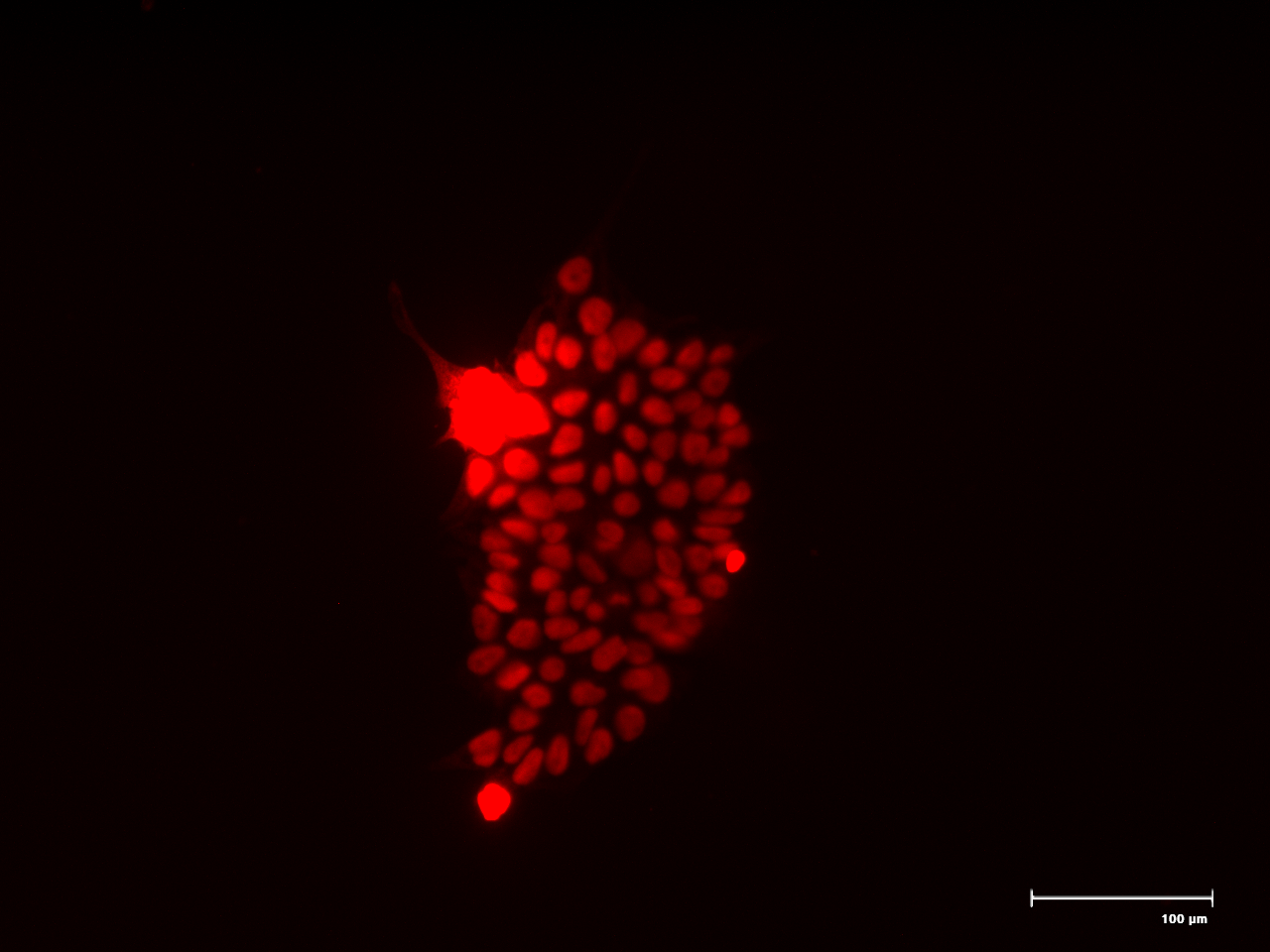

Supplement: Figure 10—source data 2. [file elife-104045-fig10-data2.zip › Figure 10B Source data iPSC IF/iPSC IF/unprocessed/oct4r.tif]

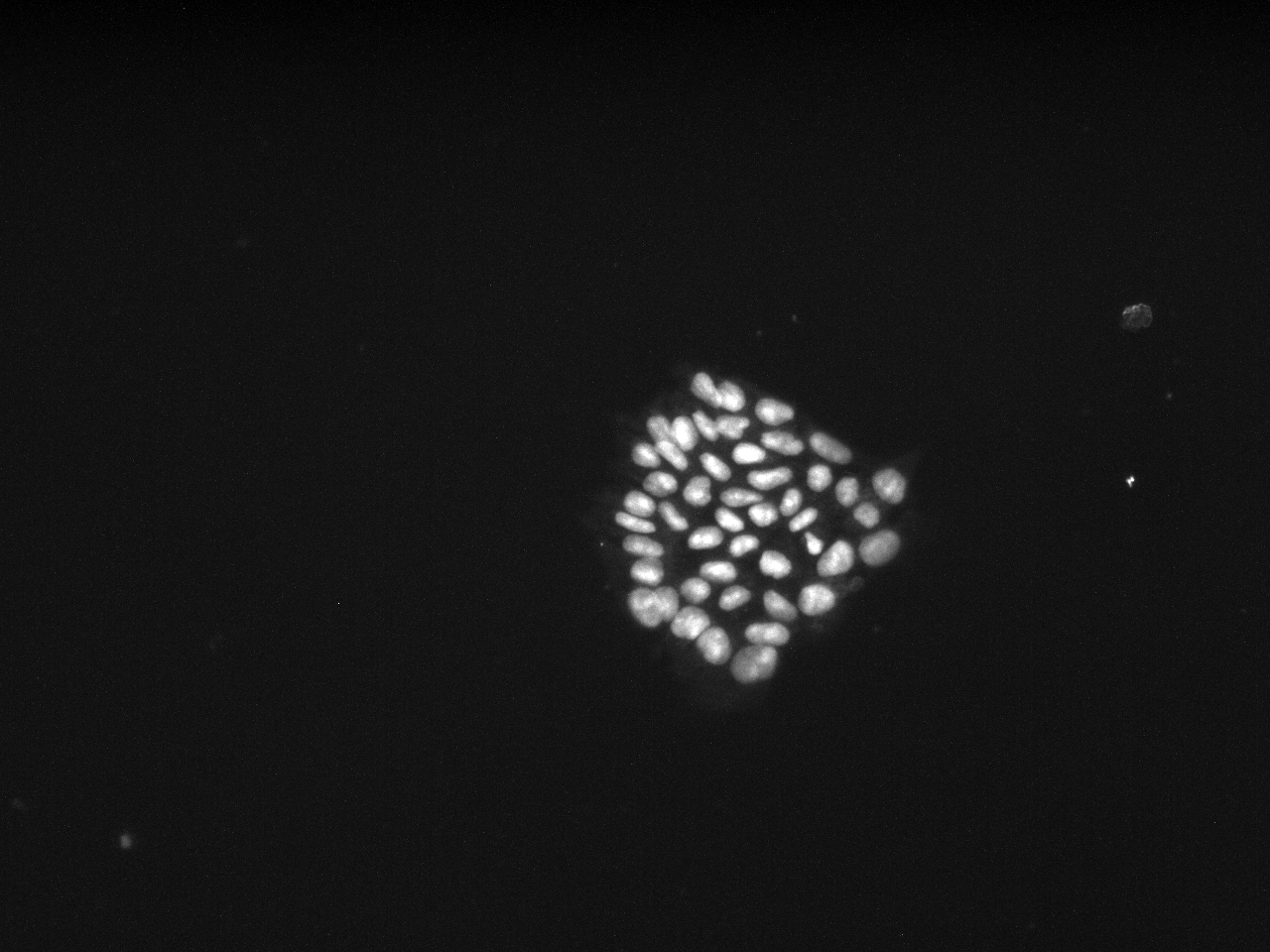

Supplement: Figure 10—source data 2. [file elife-104045-fig10-data2.zip › Figure 10B Source data iPSC IF/iPSC IF/unprocessed/sox2r - Red Light.tif]

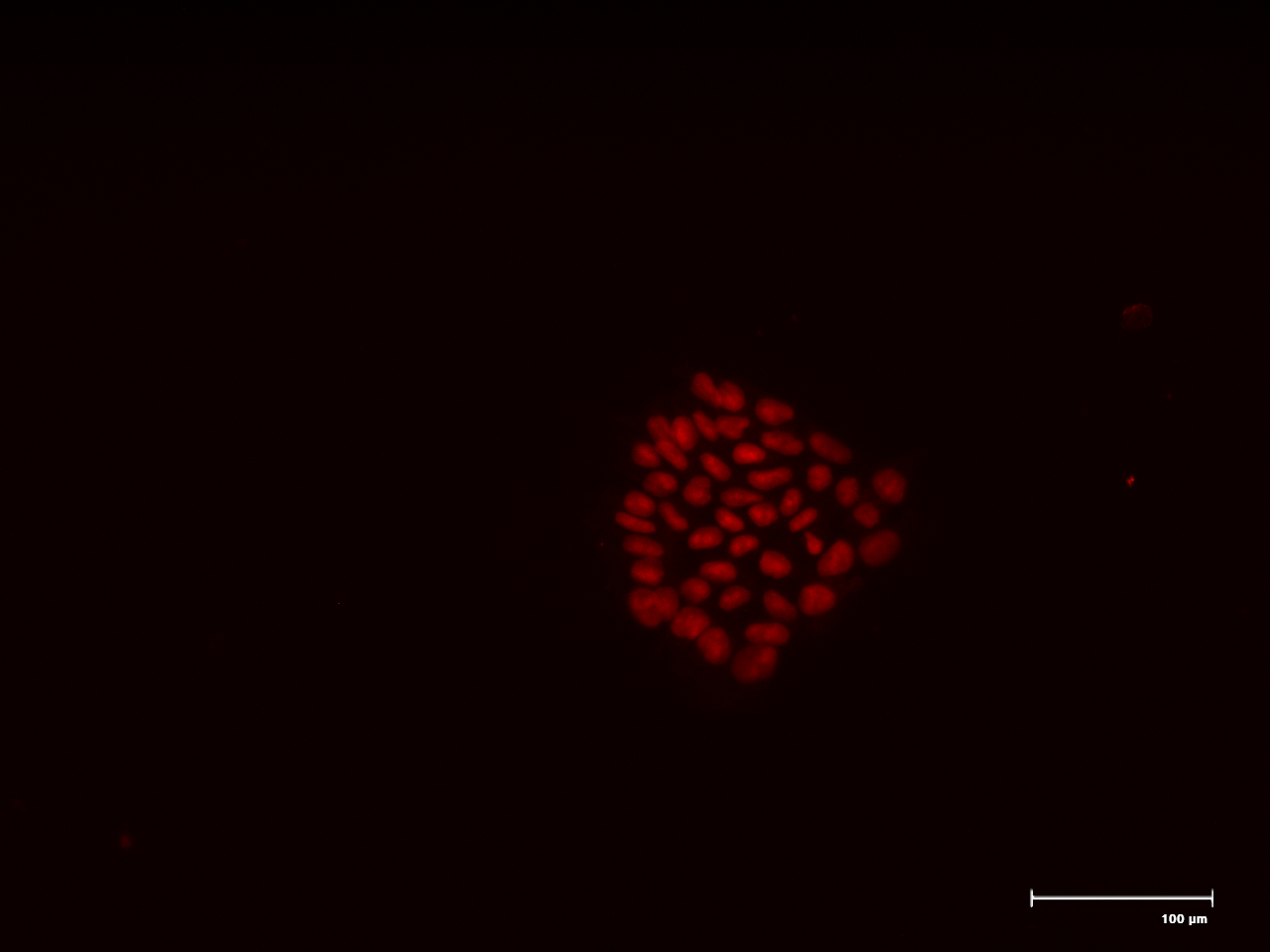

Supplement: Figure 10—source data 2. [file elife-104045-fig10-data2.zip › Figure 10B Source data iPSC IF/iPSC IF/unprocessed/sox2r.tif]

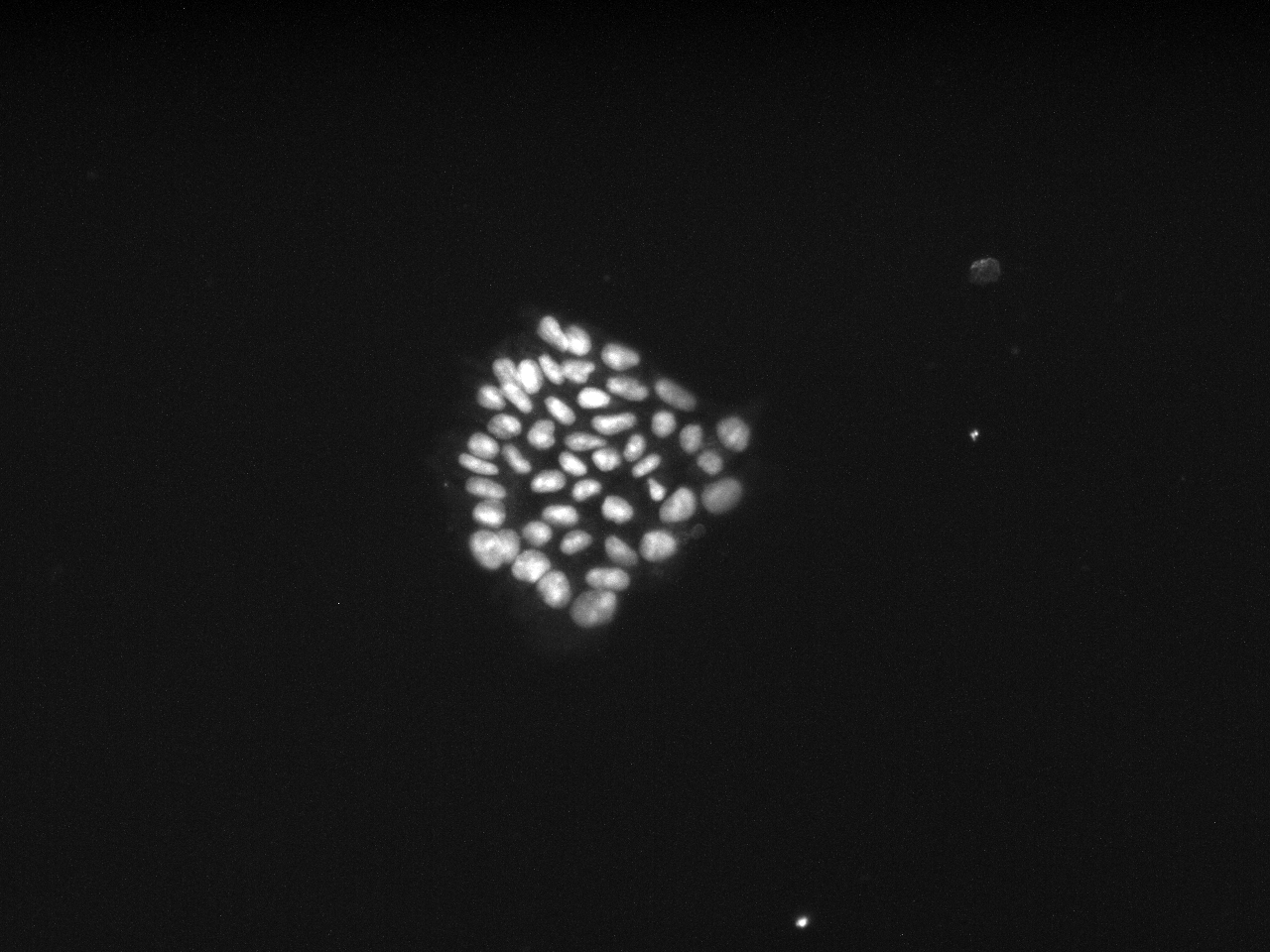

Supplement: Figure 10—source data 2. [file elife-104045-fig10-data2.zip › Figure 10B Source data iPSC IF/iPSC IF/unprocessed/sox3r - Red Light.tif]

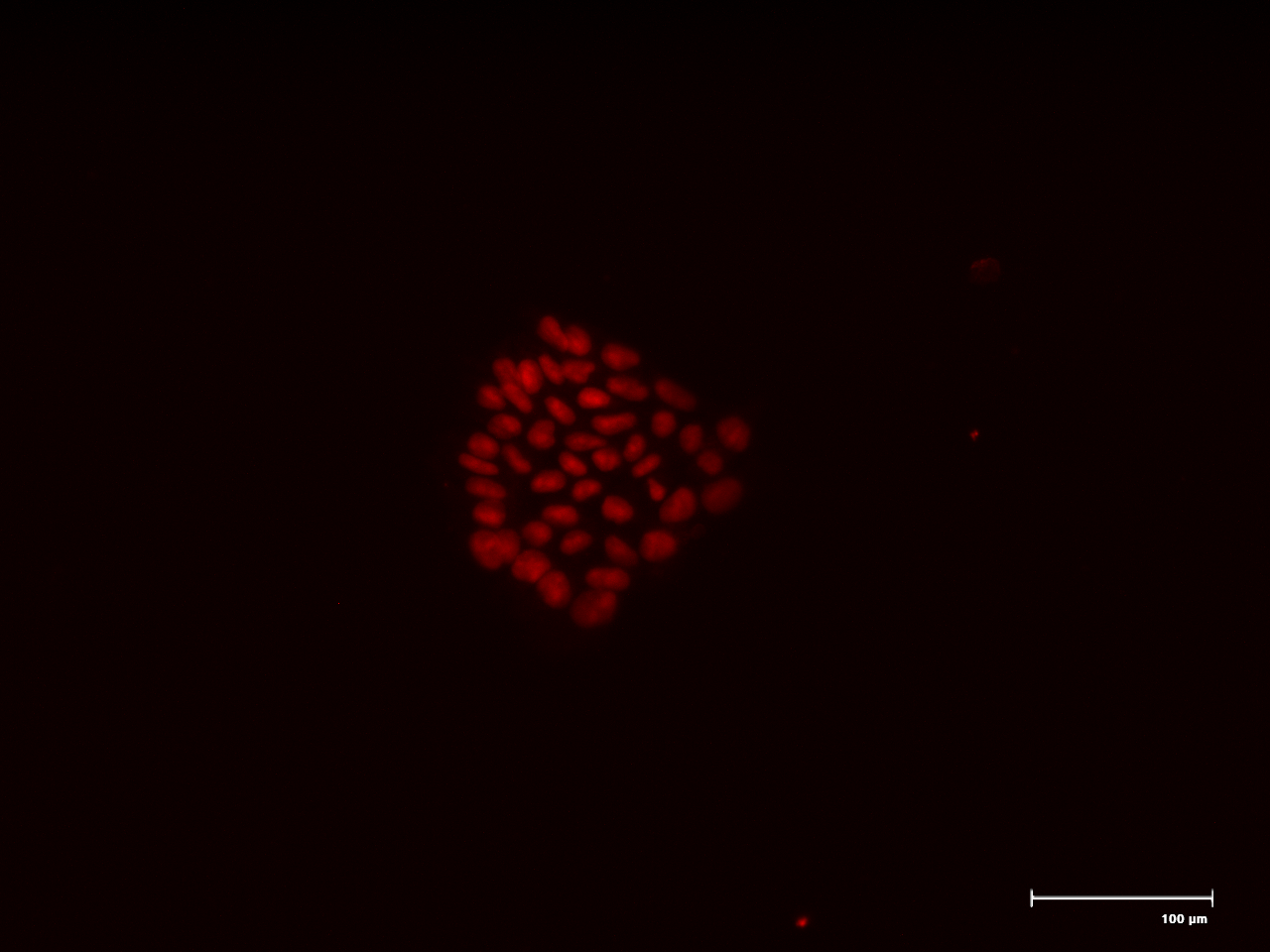

Supplement: Figure 10—source data 2. [file elife-104045-fig10-data2.zip › Figure 10B Source data iPSC IF/iPSC IF/unprocessed/sox3r.tif]

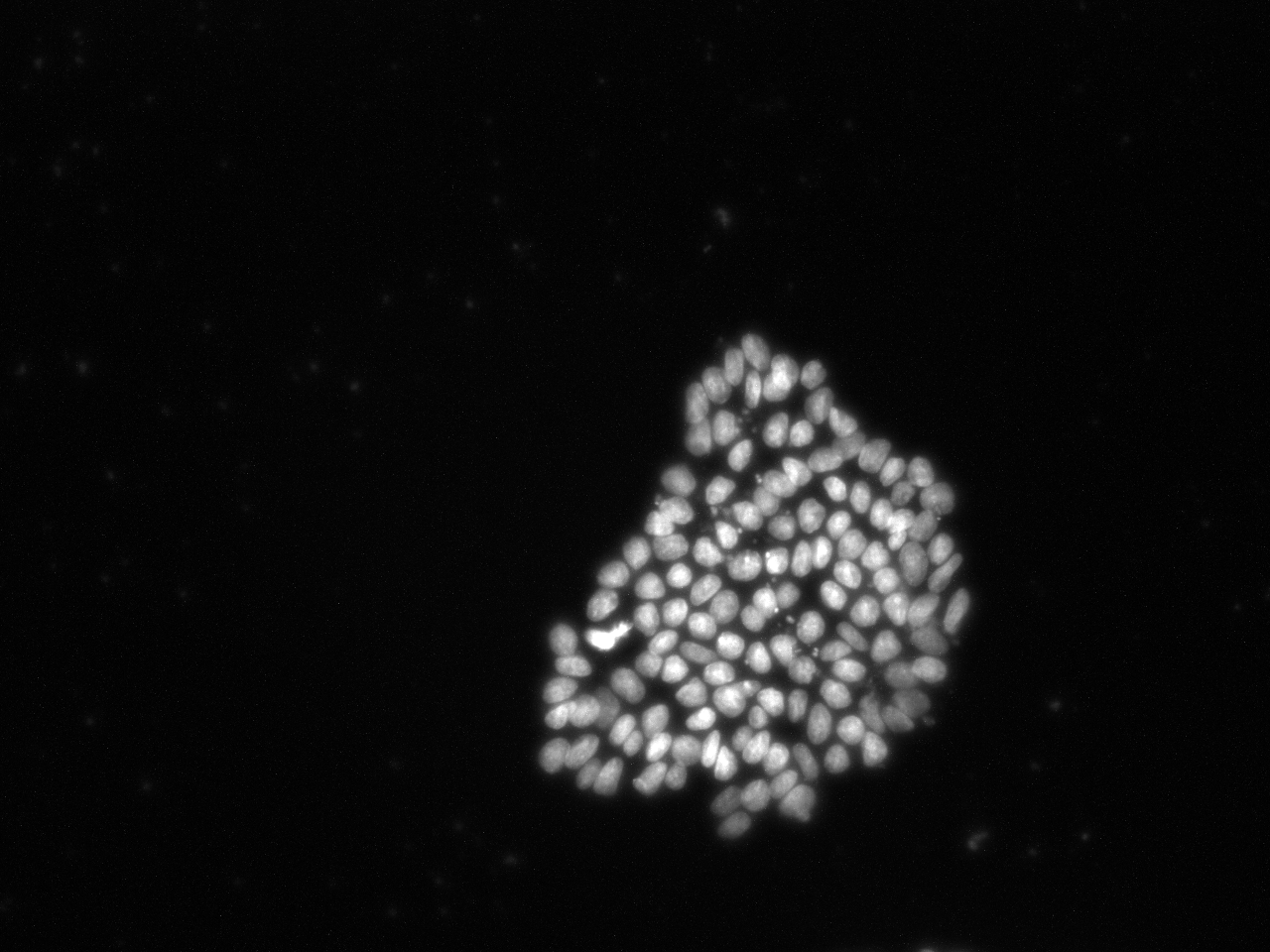

Supplement: Figure 10—source data 2. [file elife-104045-fig10-data2.zip › Figure 10B Source data iPSC IF/iPSC IF/unprocessed/soxb1 - Blue Light.tif]

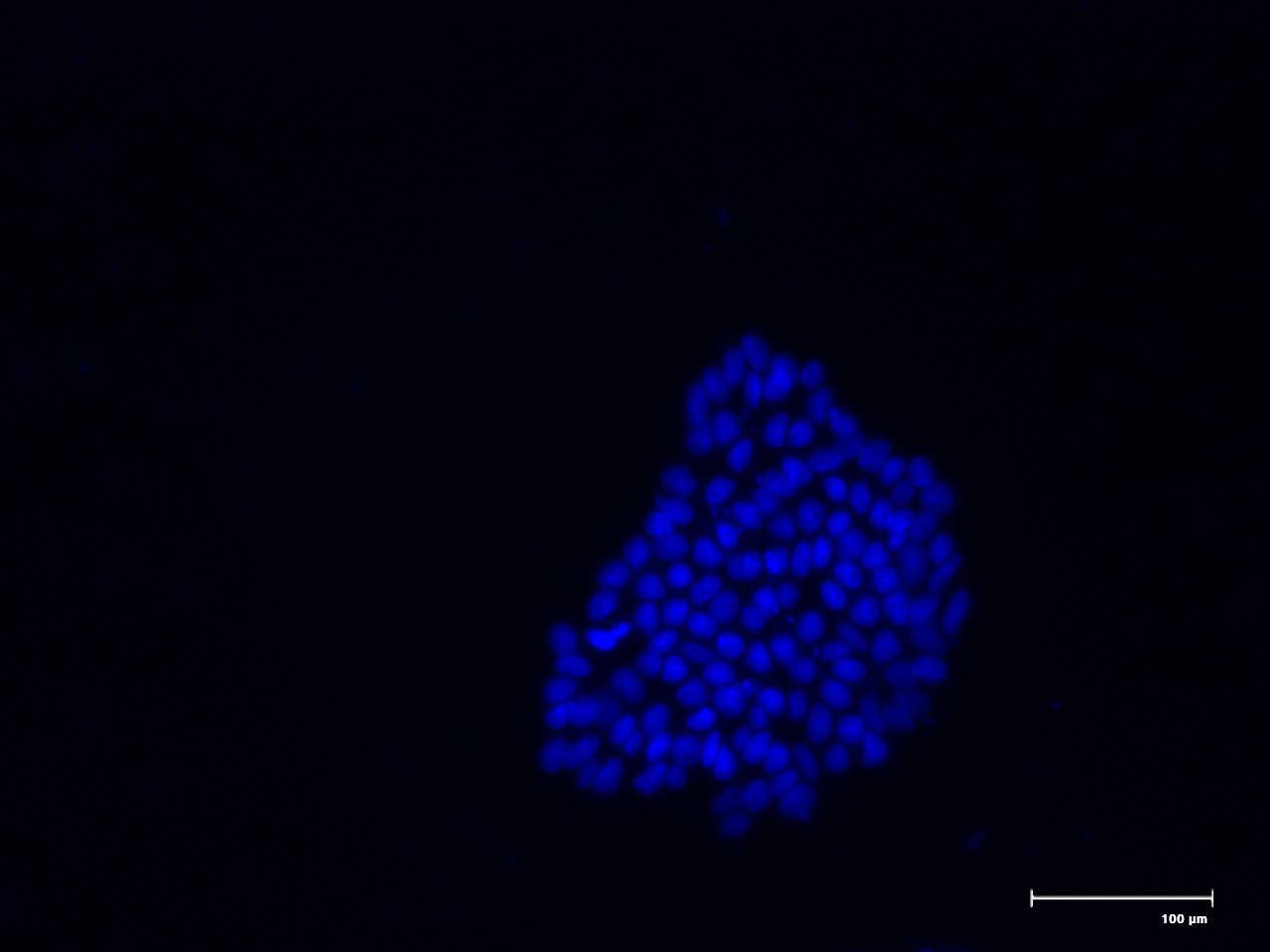

Supplement: Figure 10—source data 2. [file elife-104045-fig10-data2.zip › Figure 10B Source data iPSC IF/iPSC IF/unprocessed/soxb1.tif]

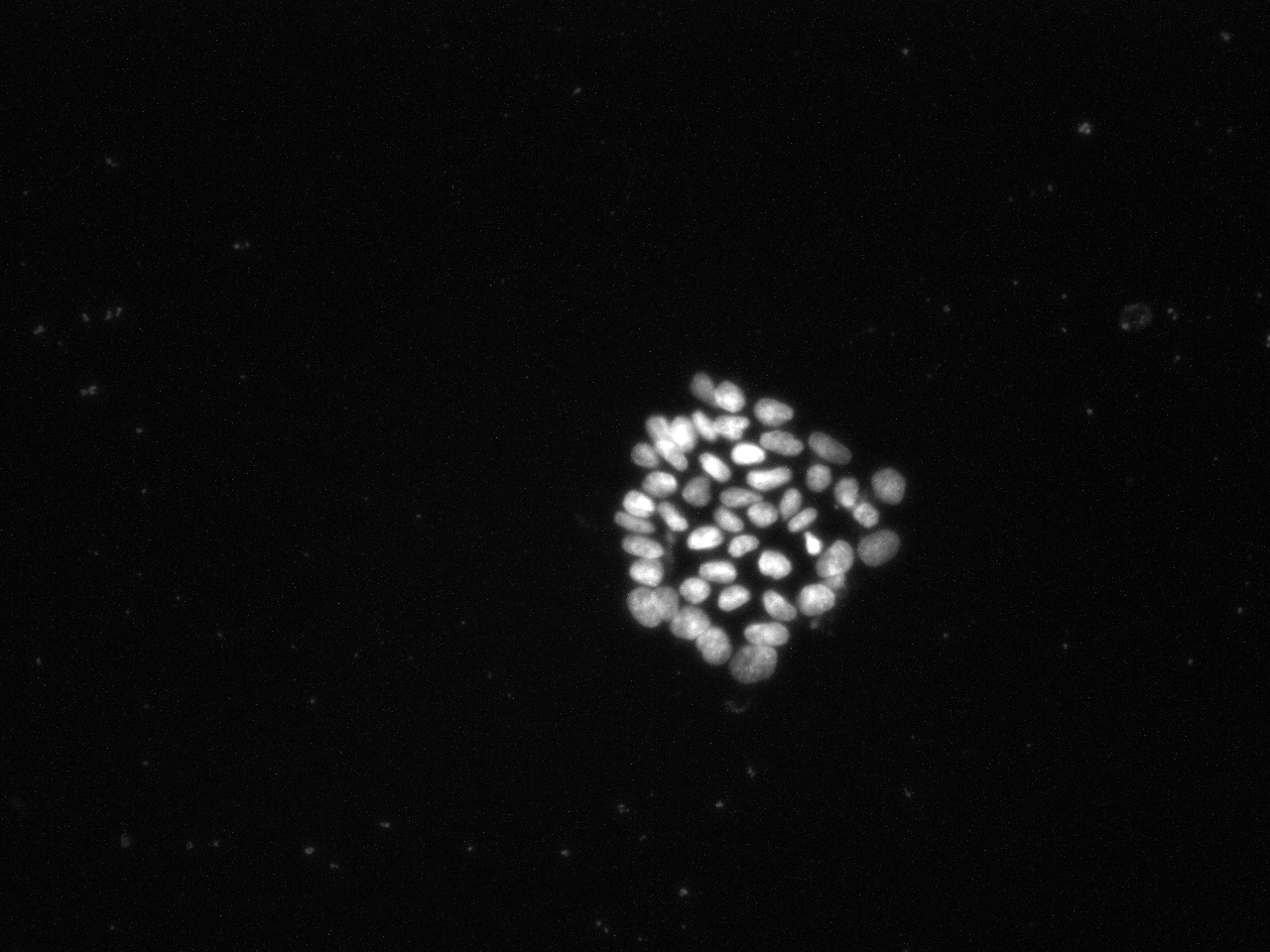

Supplement: Figure 10—source data 2. [file elife-104045-fig10-data2.zip › Figure 10B Source data iPSC IF/iPSC IF/unprocessed/soxb2 - Blue Light.tif]

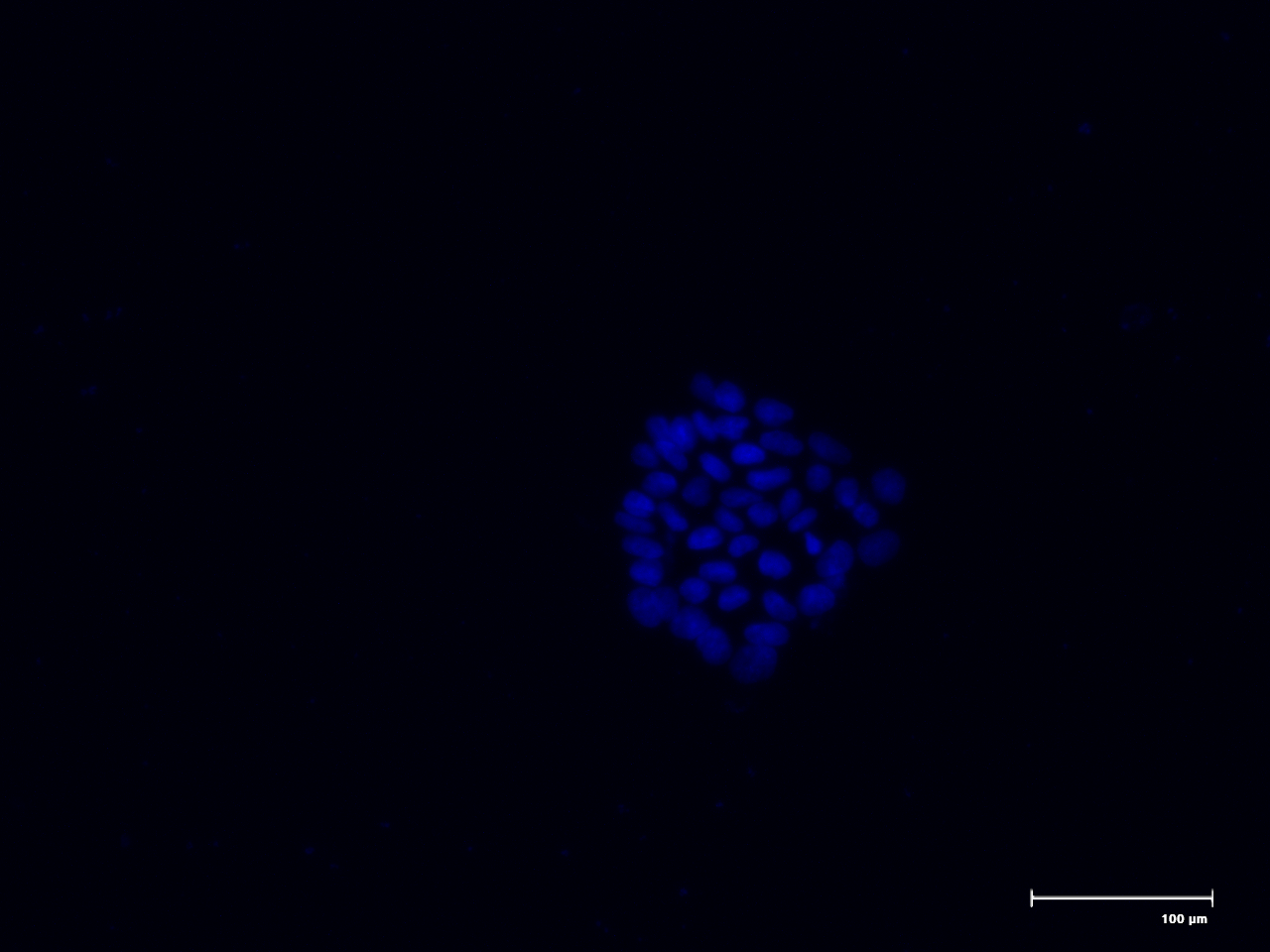

Supplement: Figure 10—source data 2. [file elife-104045-fig10-data2.zip › Figure 10B Source data iPSC IF/iPSC IF/unprocessed/soxb2.tif]

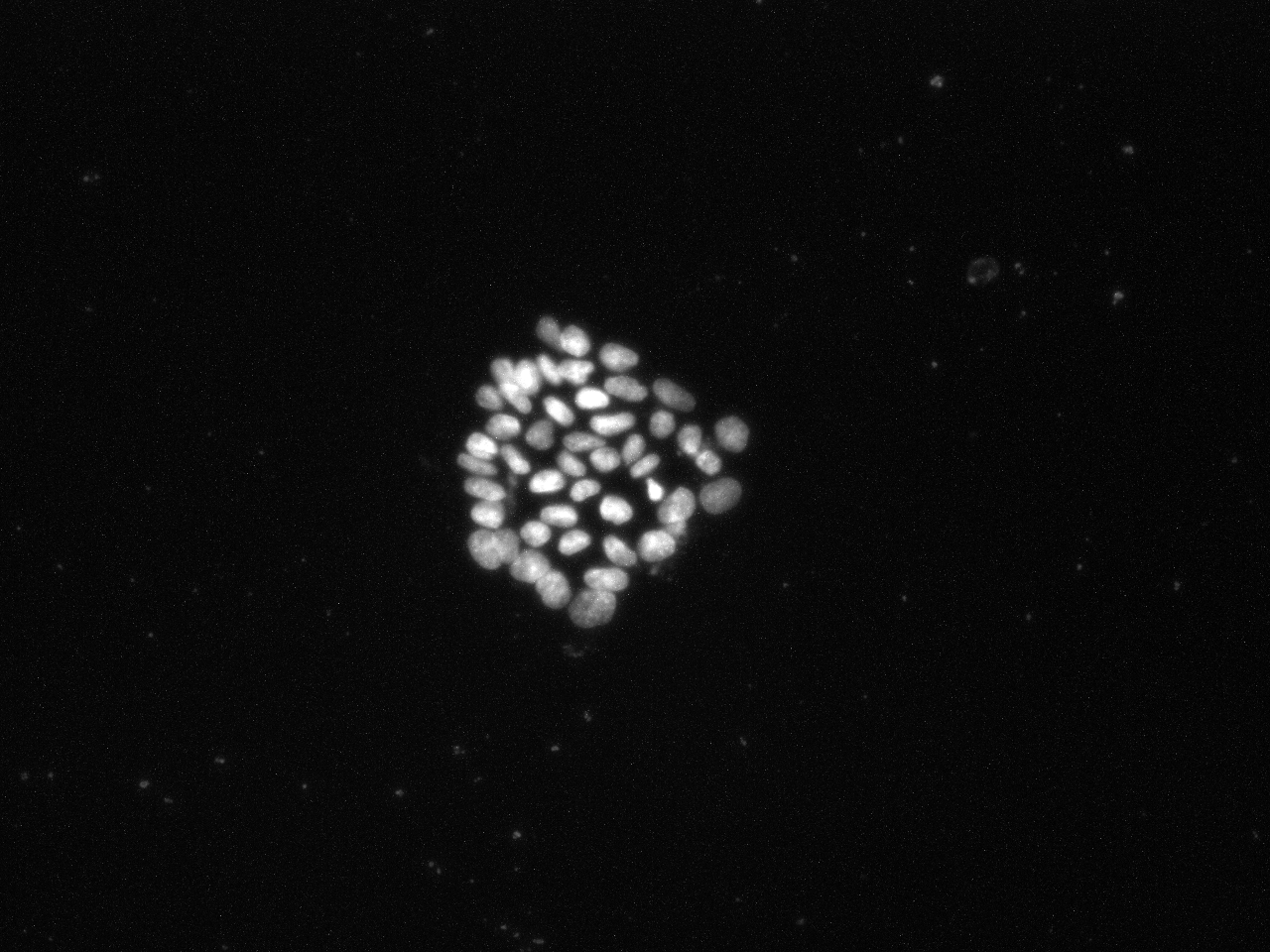

Supplement: Figure 10—source data 2. [file elife-104045-fig10-data2.zip › Figure 10B Source data iPSC IF/iPSC IF/unprocessed/soxb4 - Blue Light.tif]

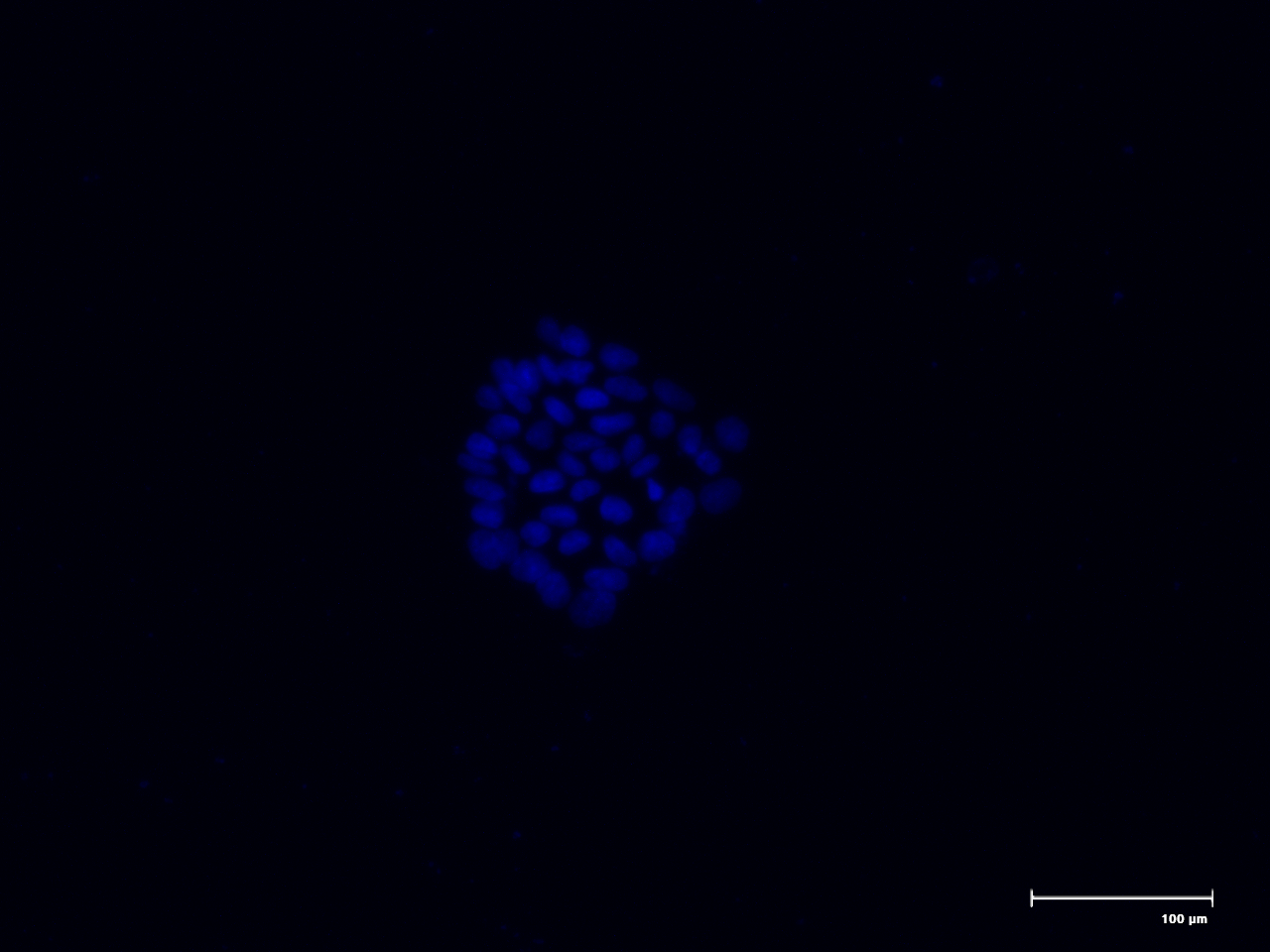

Supplement: Figure 10—source data 2. [file elife-104045-fig10-data2.zip › Figure 10B Source data iPSC IF/iPSC IF/unprocessed/soxb4.tif]

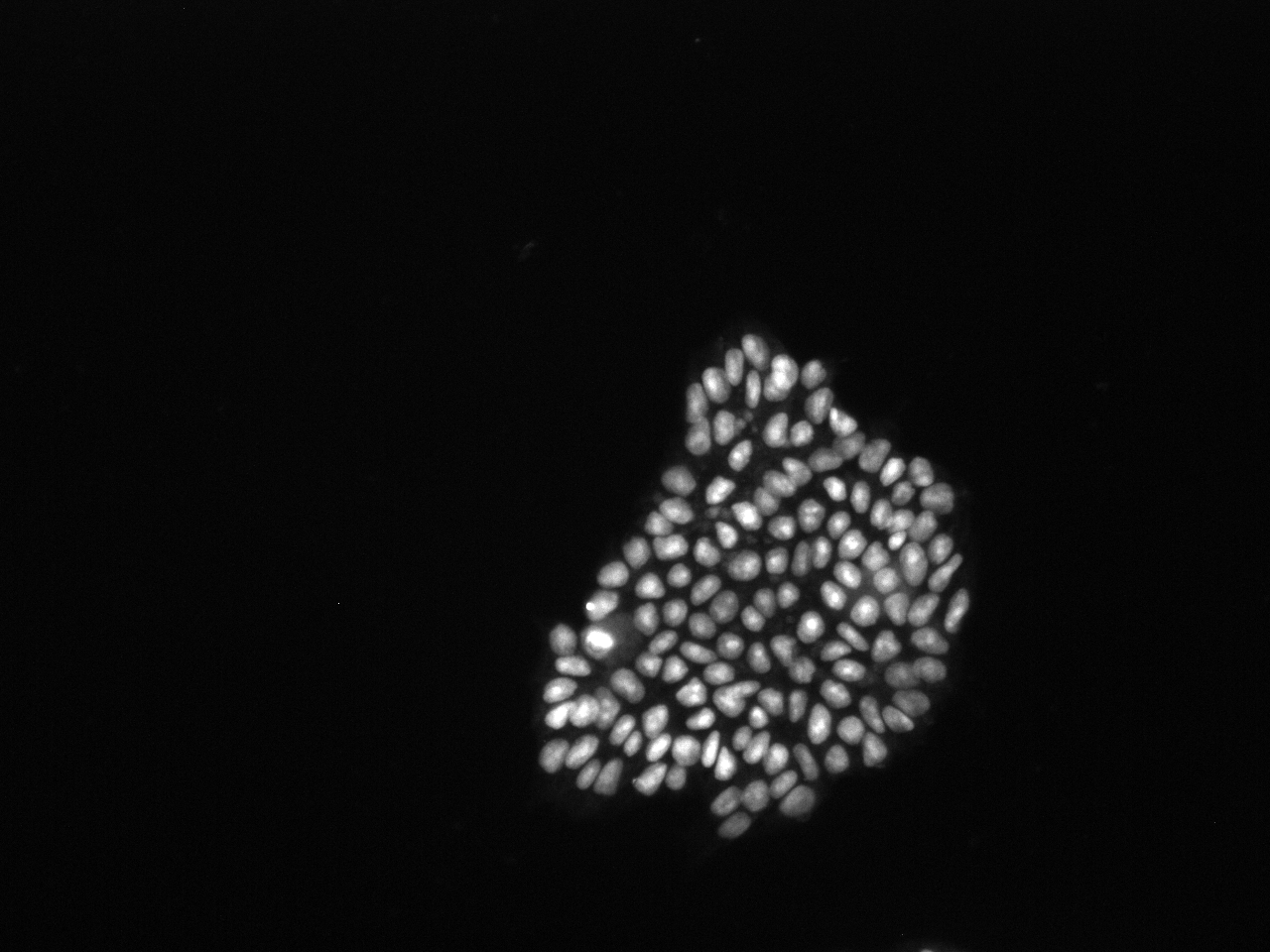

Supplement: Figure 10—source data 2. [file elife-104045-fig10-data2.zip › Figure 10B Source data iPSC IF/iPSC IF/unprocessed/soxr1 - Red Light.tif]

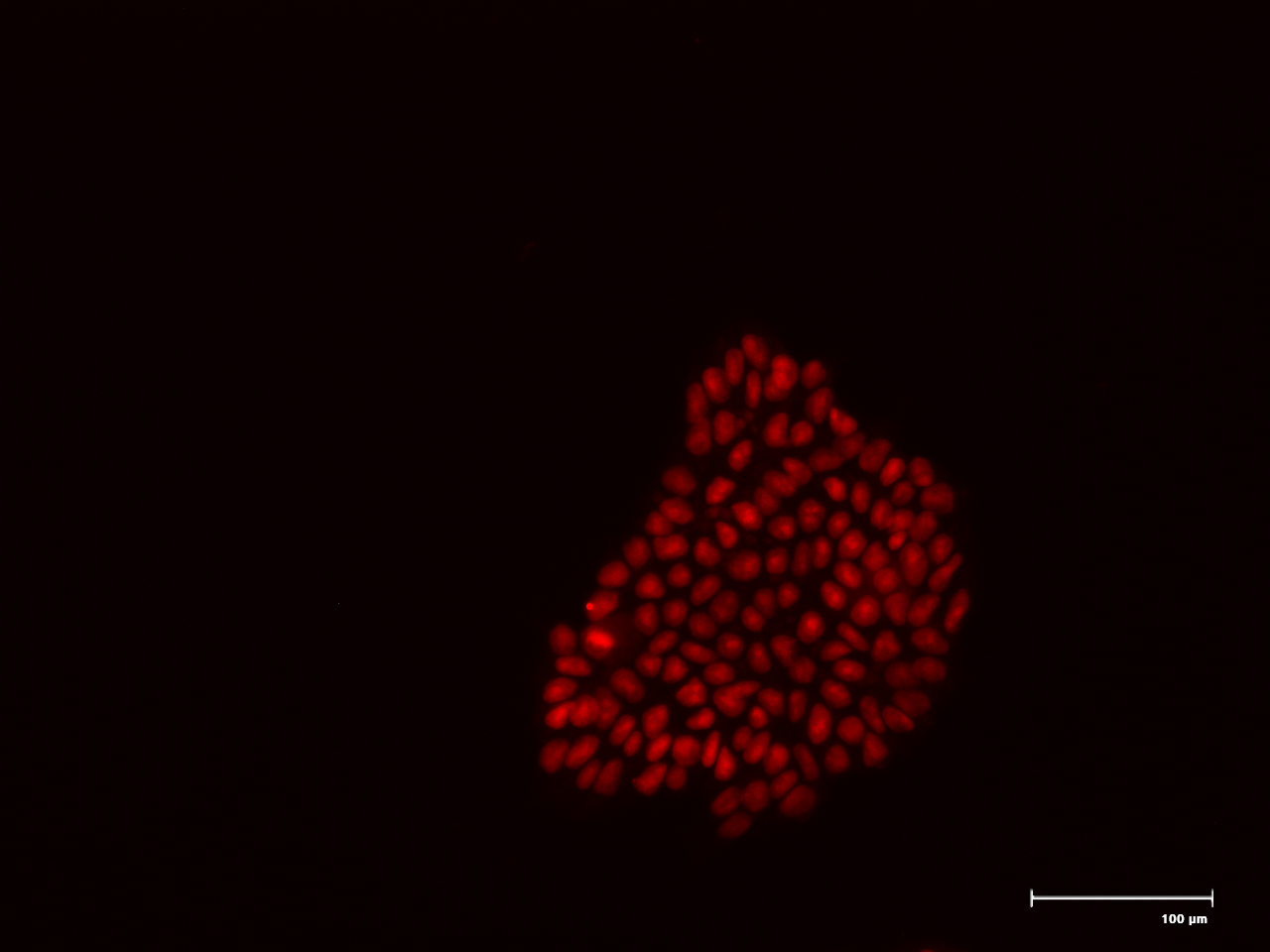

Supplement: Figure 10—source data 2. [file elife-104045-fig10-data2.zip › Figure 10B Source data iPSC IF/iPSC IF/unprocessed/soxr1.tif]

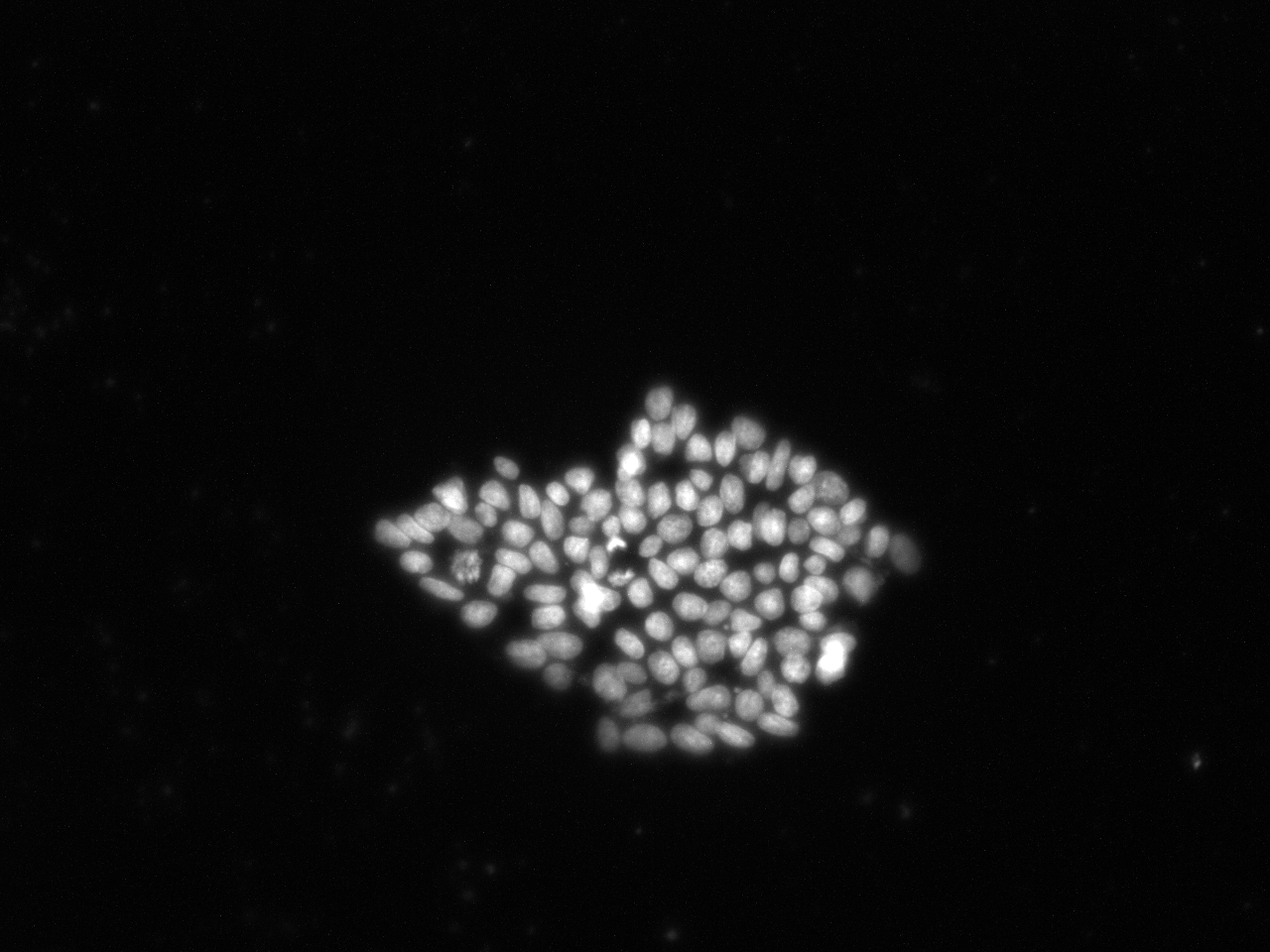

Supplement: Figure 10—source data 2. [file elife-104045-fig10-data2.zip › Figure 10B Source data iPSC IF/iPSC IF/unprocessed/sseab2 - Blue Light.tif]

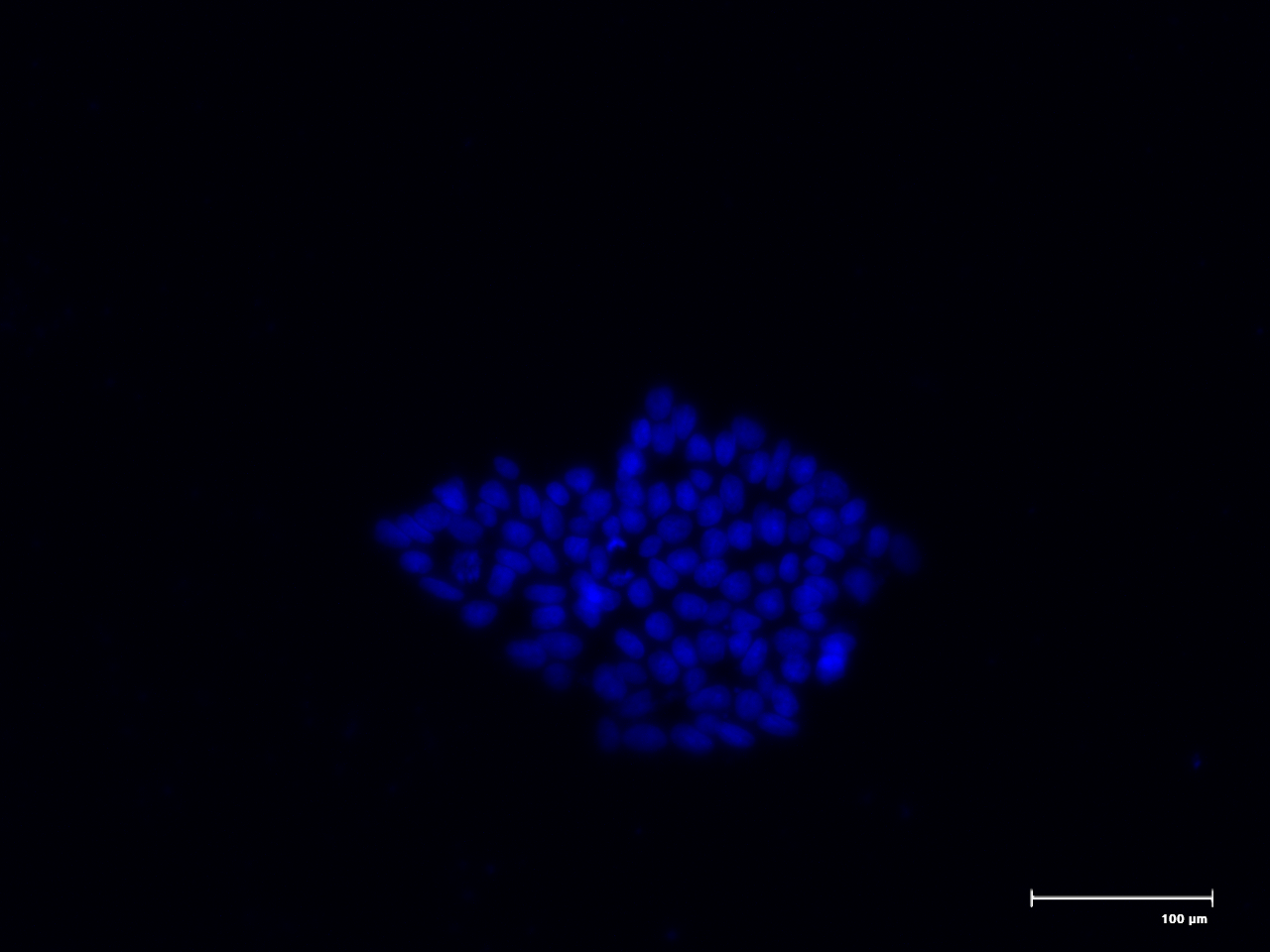

Supplement: Figure 10—source data 2. [file elife-104045-fig10-data2.zip › Figure 10B Source data iPSC IF/iPSC IF/unprocessed/sseab2.tif]

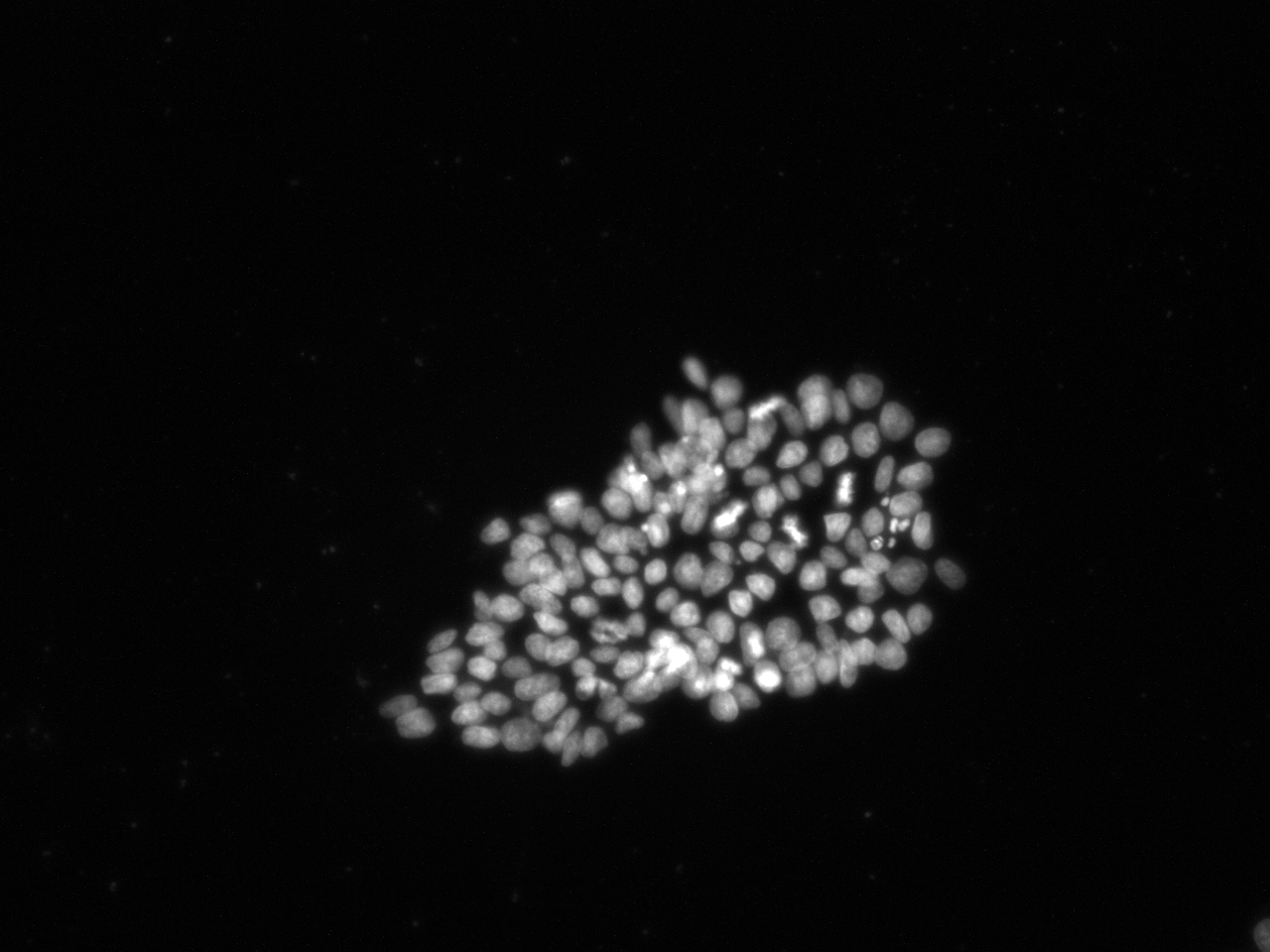

Supplement: Figure 10—source data 2. [file elife-104045-fig10-data2.zip › Figure 10B Source data iPSC IF/iPSC IF/unprocessed/sseag - Blue Light.tif]

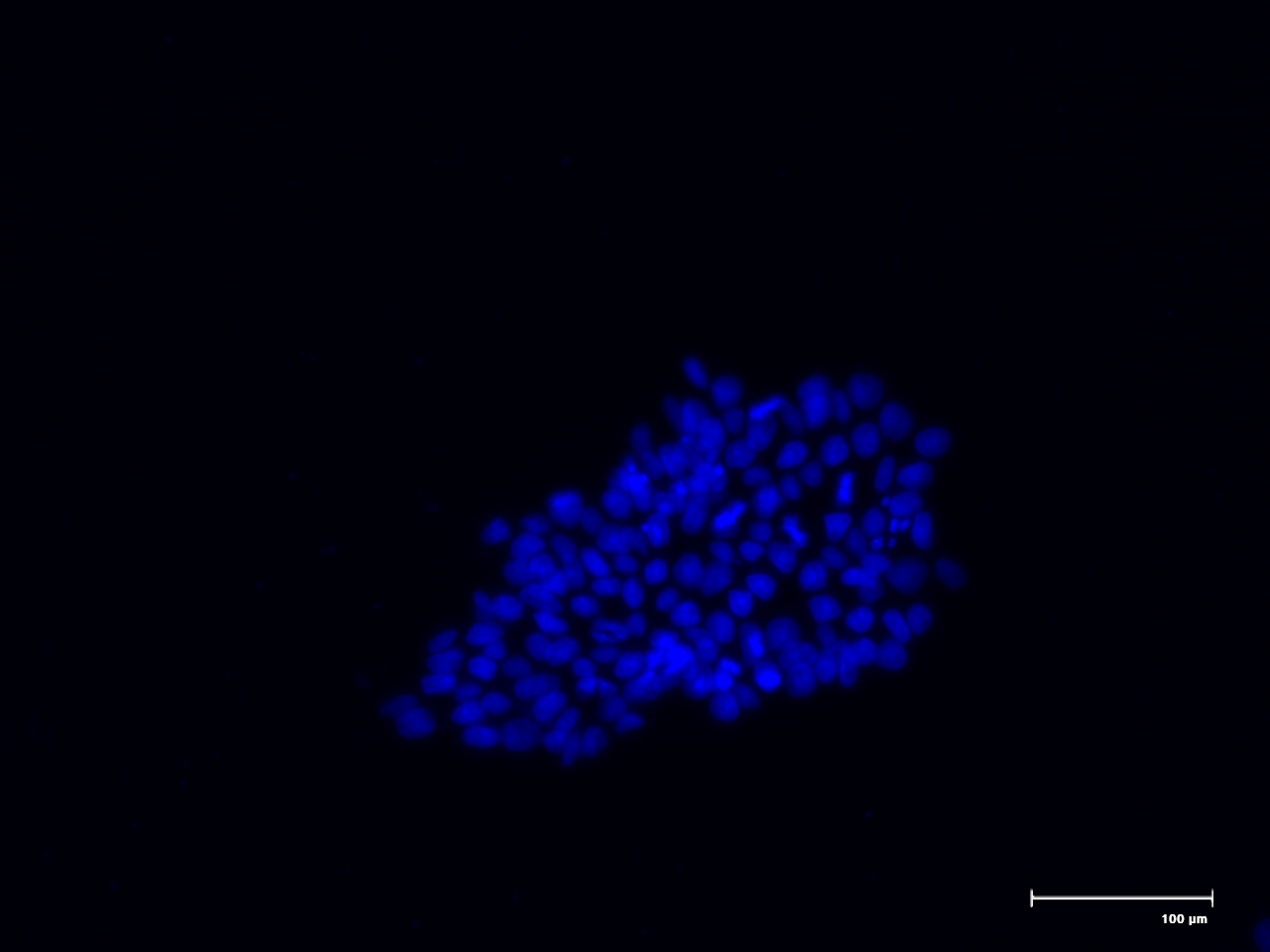

Supplement: Figure 10—source data 2. [file elife-104045-fig10-data2.zip › Figure 10B Source data iPSC IF/iPSC IF/unprocessed/sseag.tif]

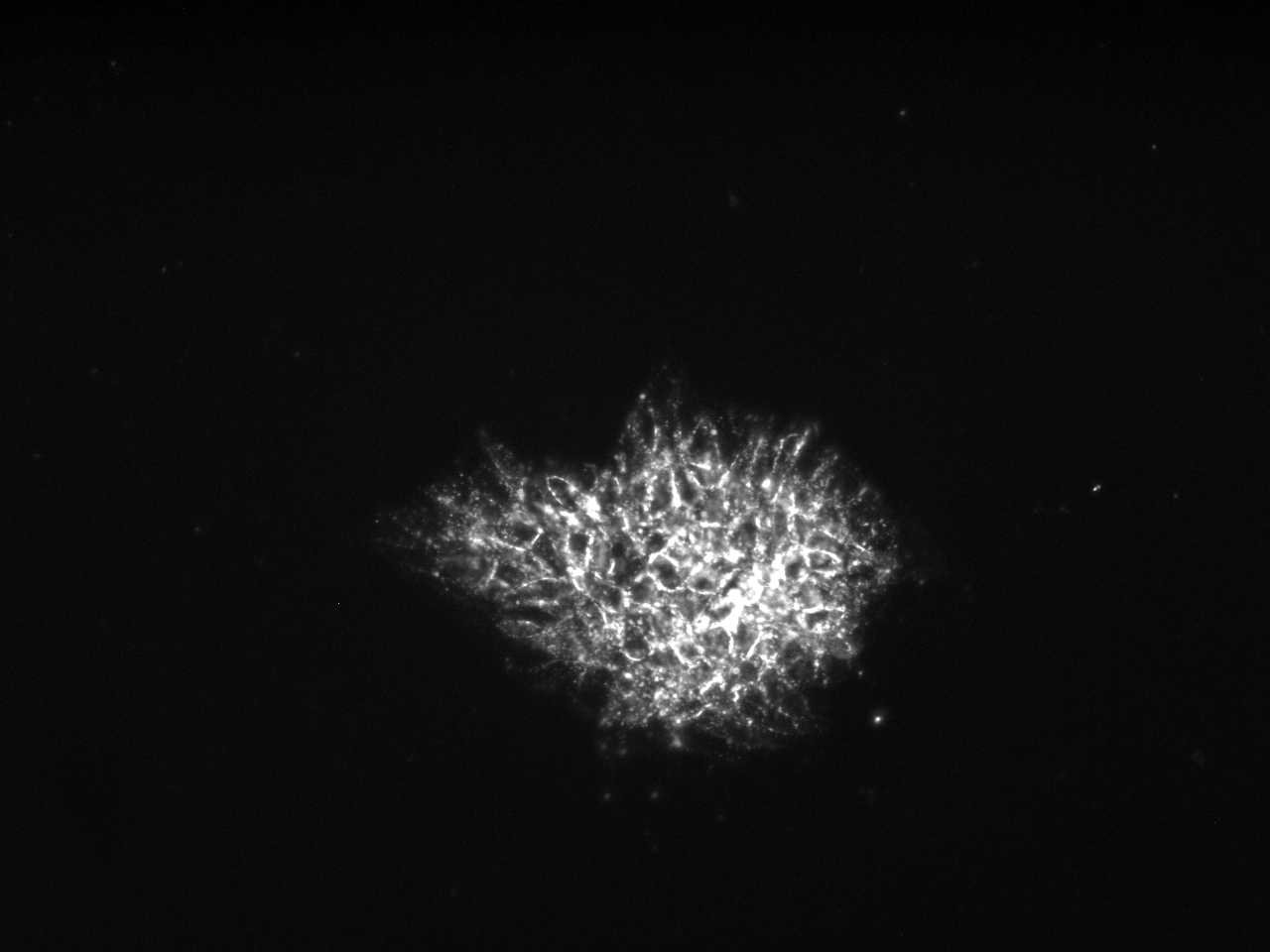

Supplement: Figure 10—source data 2. [file elife-104045-fig10-data2.zip › Figure 10B Source data iPSC IF/iPSC IF/unprocessed/sseag2 - Green Light.tif]

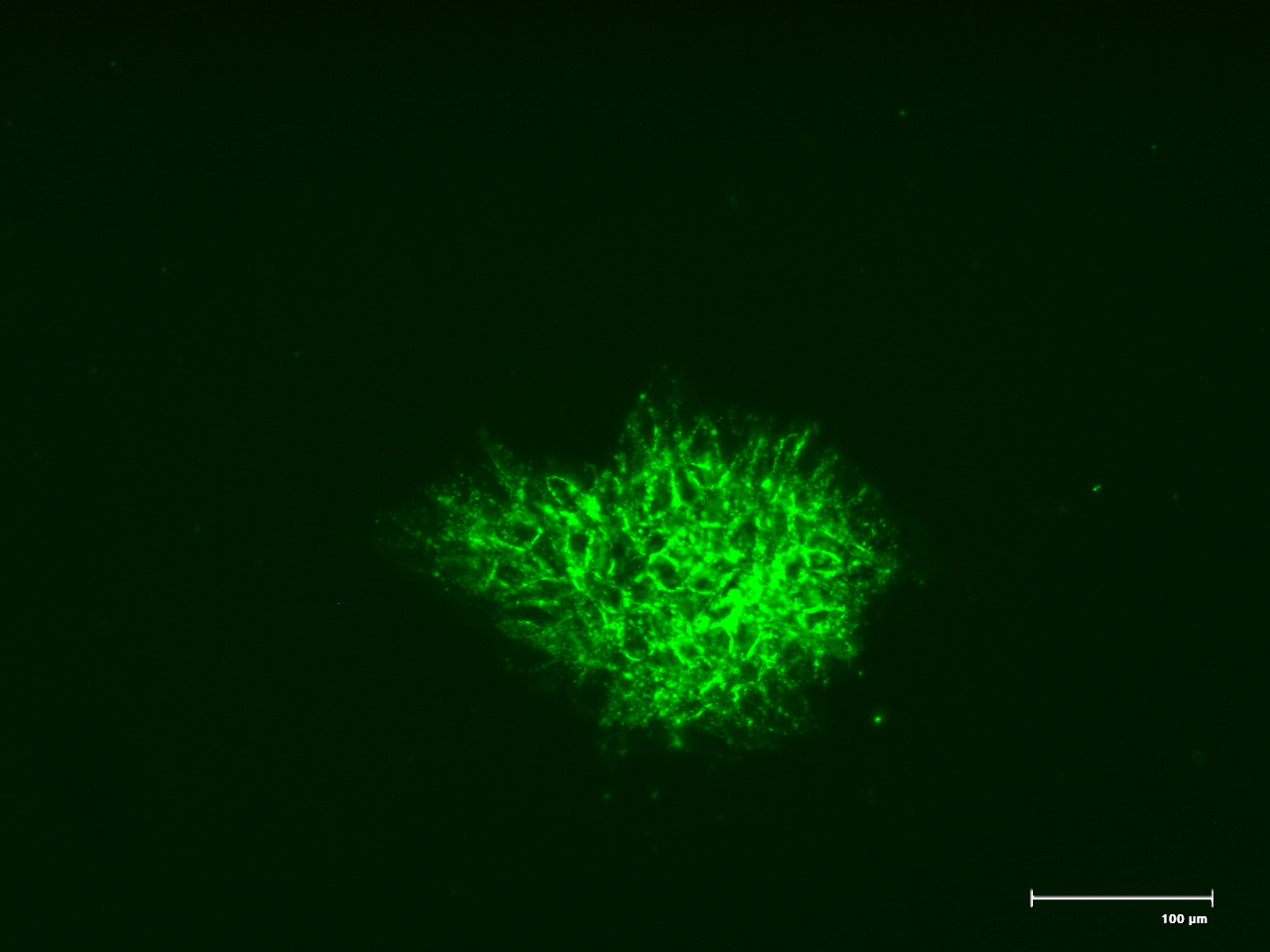

Supplement: Figure 10—source data 2. [file elife-104045-fig10-data2.zip › Figure 10B Source data iPSC IF/iPSC IF/unprocessed/sseag2.tif]

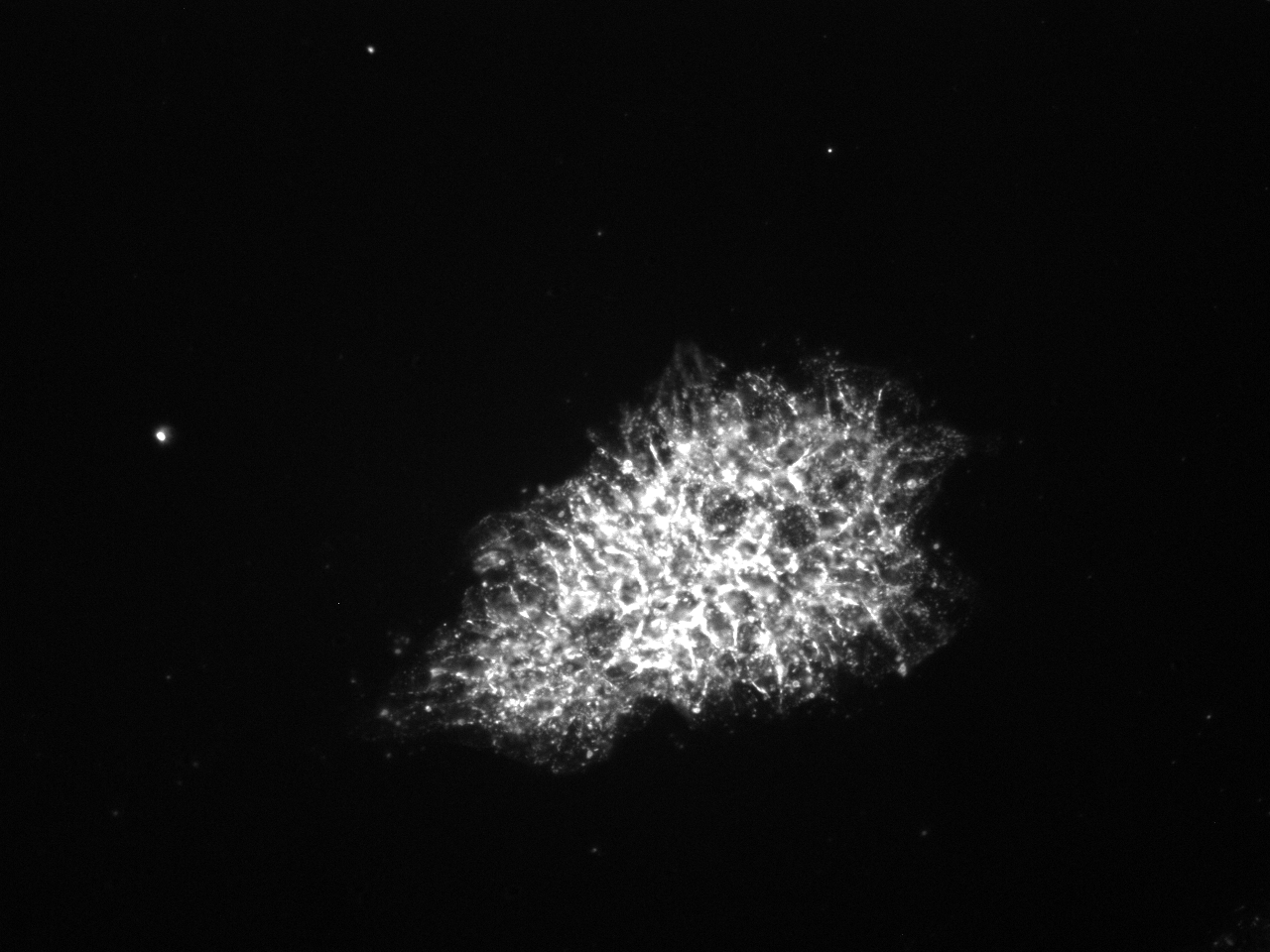

Supplement: Figure 10—source data 2. [file elife-104045-fig10-data2.zip › Figure 10B Source data iPSC IF/iPSC IF/unprocessed/sseagg - Green Light.tif]

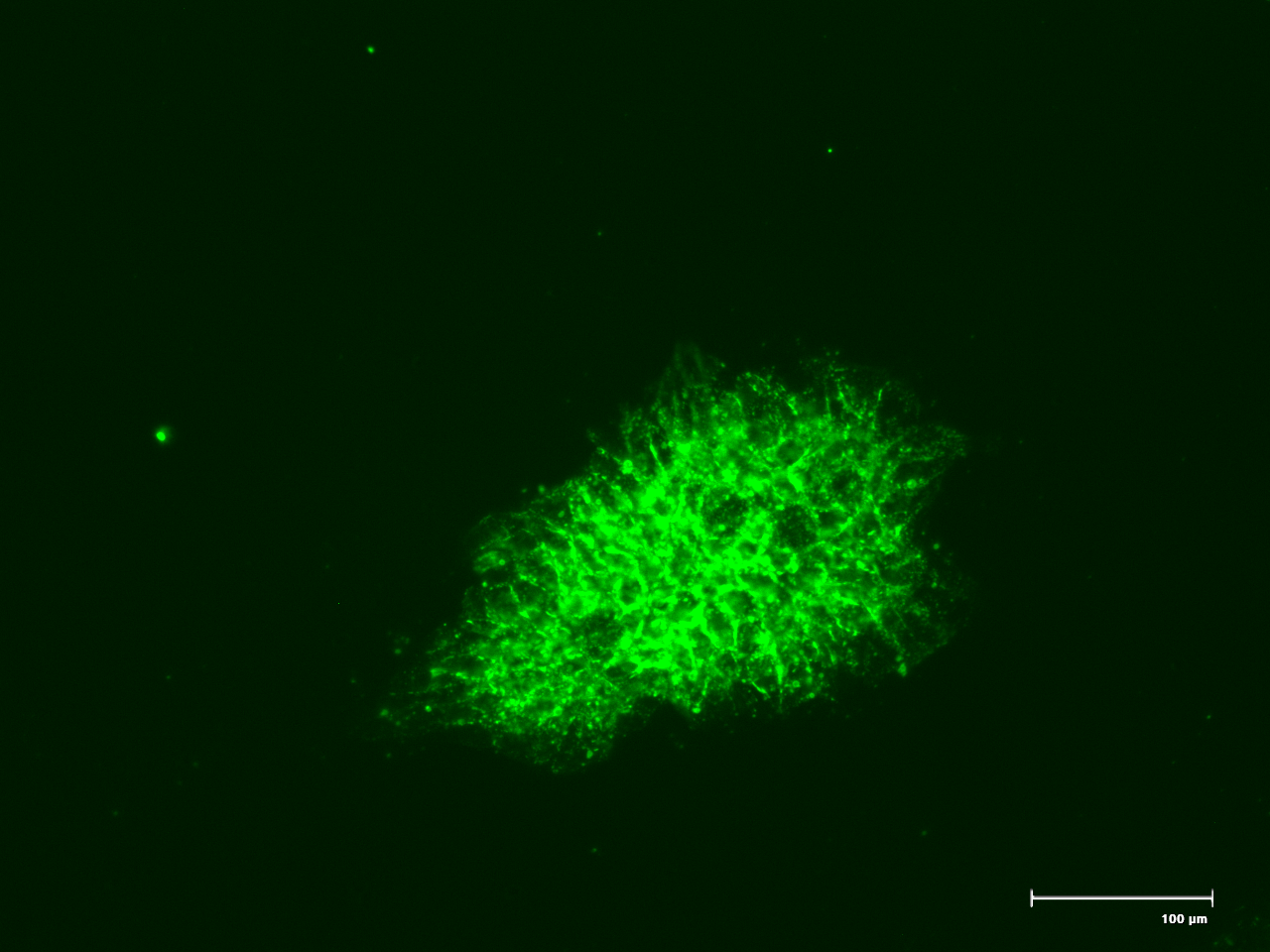

Supplement: Figure 10—source data 2. [file elife-104045-fig10-data2.zip › Figure 10B Source data iPSC IF/iPSC IF/unprocessed/sseagg.tif]

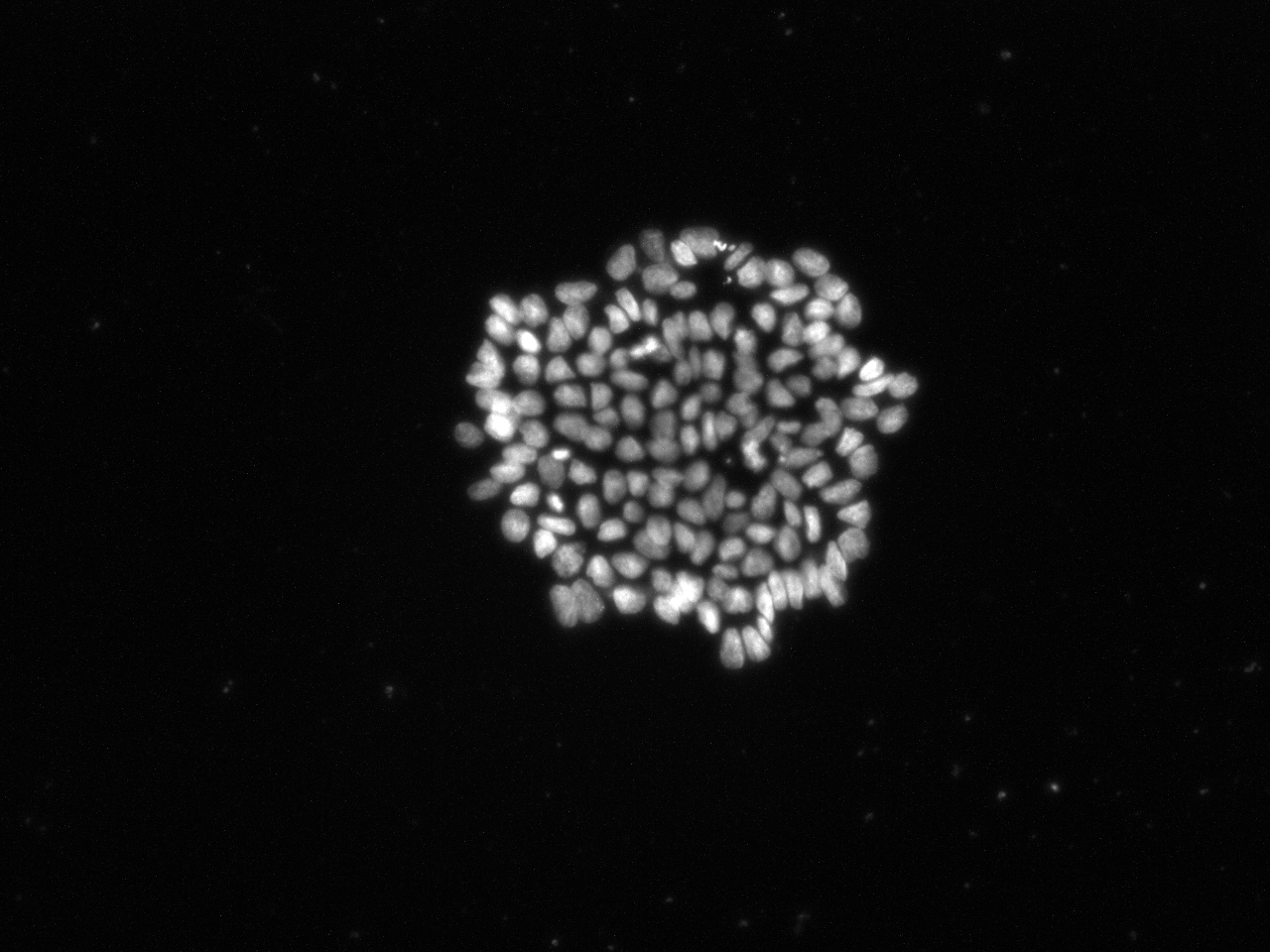

Supplement: Figure 10—source data 2. [file elife-104045-fig10-data2.zip › Figure 10B Source data iPSC IF/iPSC IF/unprocessed/tra2ab - Blue Light.tif]

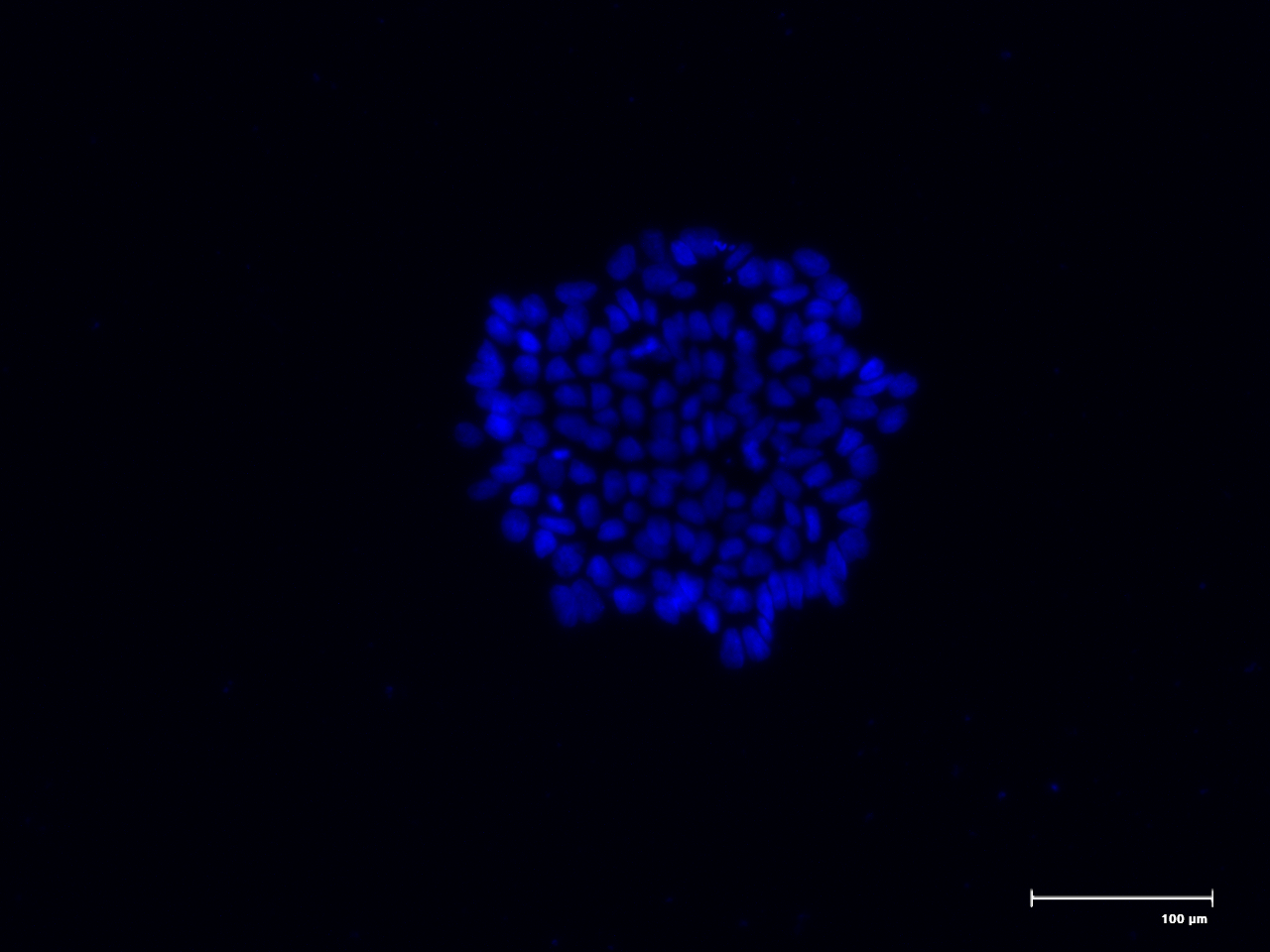

Supplement: Figure 10—source data 2. [file elife-104045-fig10-data2.zip › Figure 10B Source data iPSC IF/iPSC IF/unprocessed/tra2ab.tif]

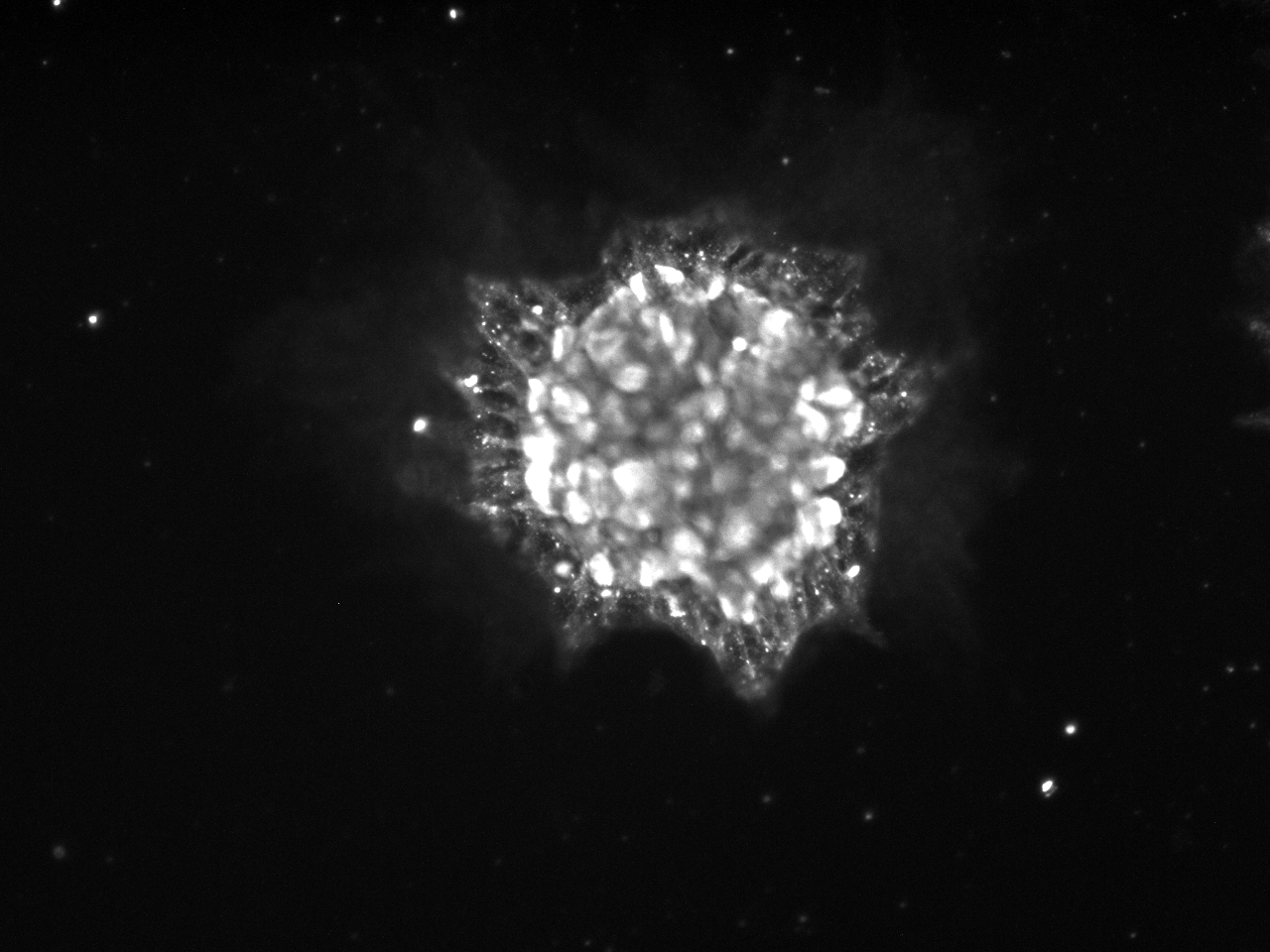

Supplement: Figure 10—source data 2. [file elife-104045-fig10-data2.zip › Figure 10B Source data iPSC IF/iPSC IF/unprocessed/tra2ag - Green Light.tif]

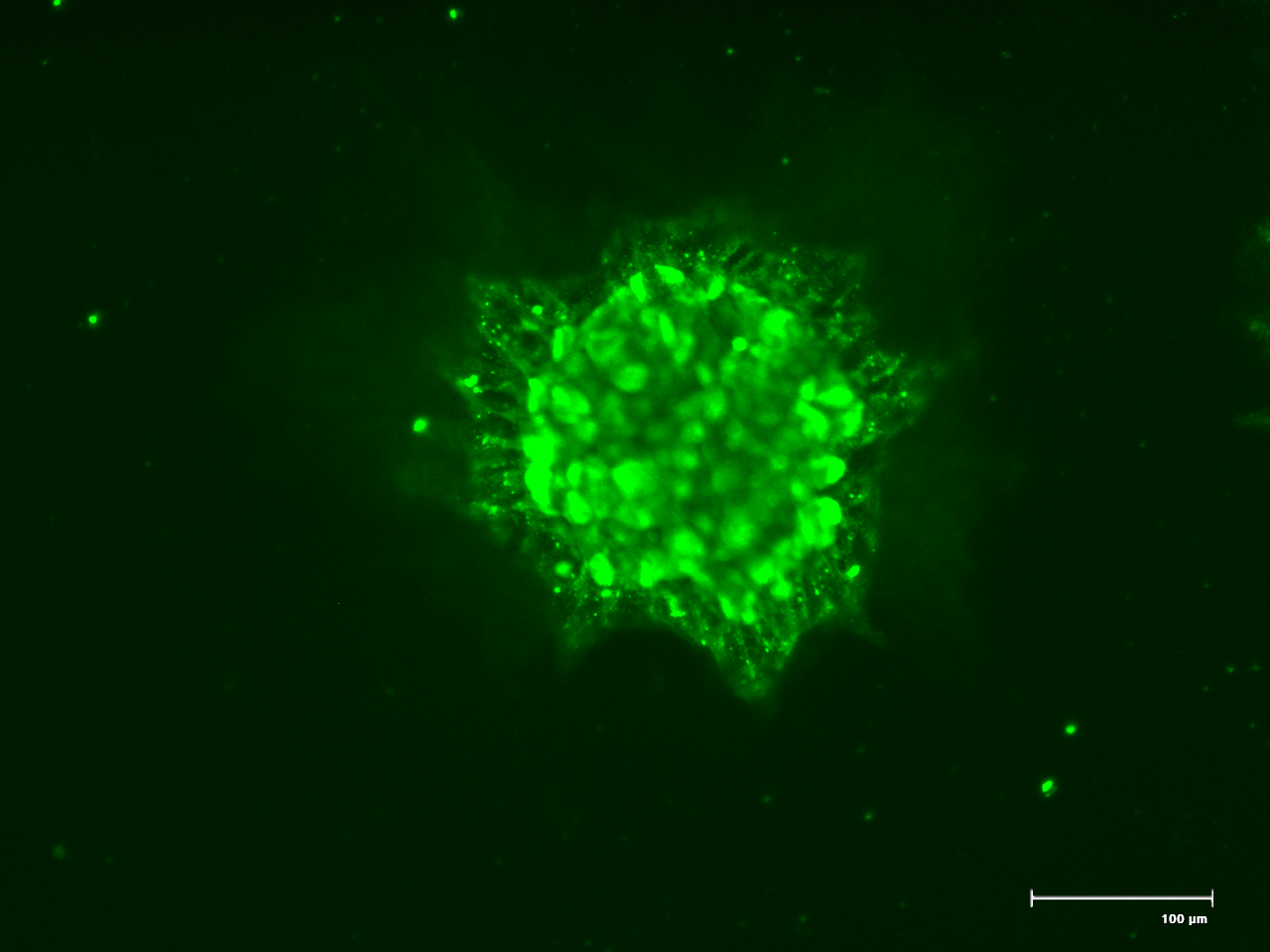

Supplement: Figure 10—source data 2. [file elife-104045-fig10-data2.zip › Figure 10B Source data iPSC IF/iPSC IF/unprocessed/tra2ag.tif]

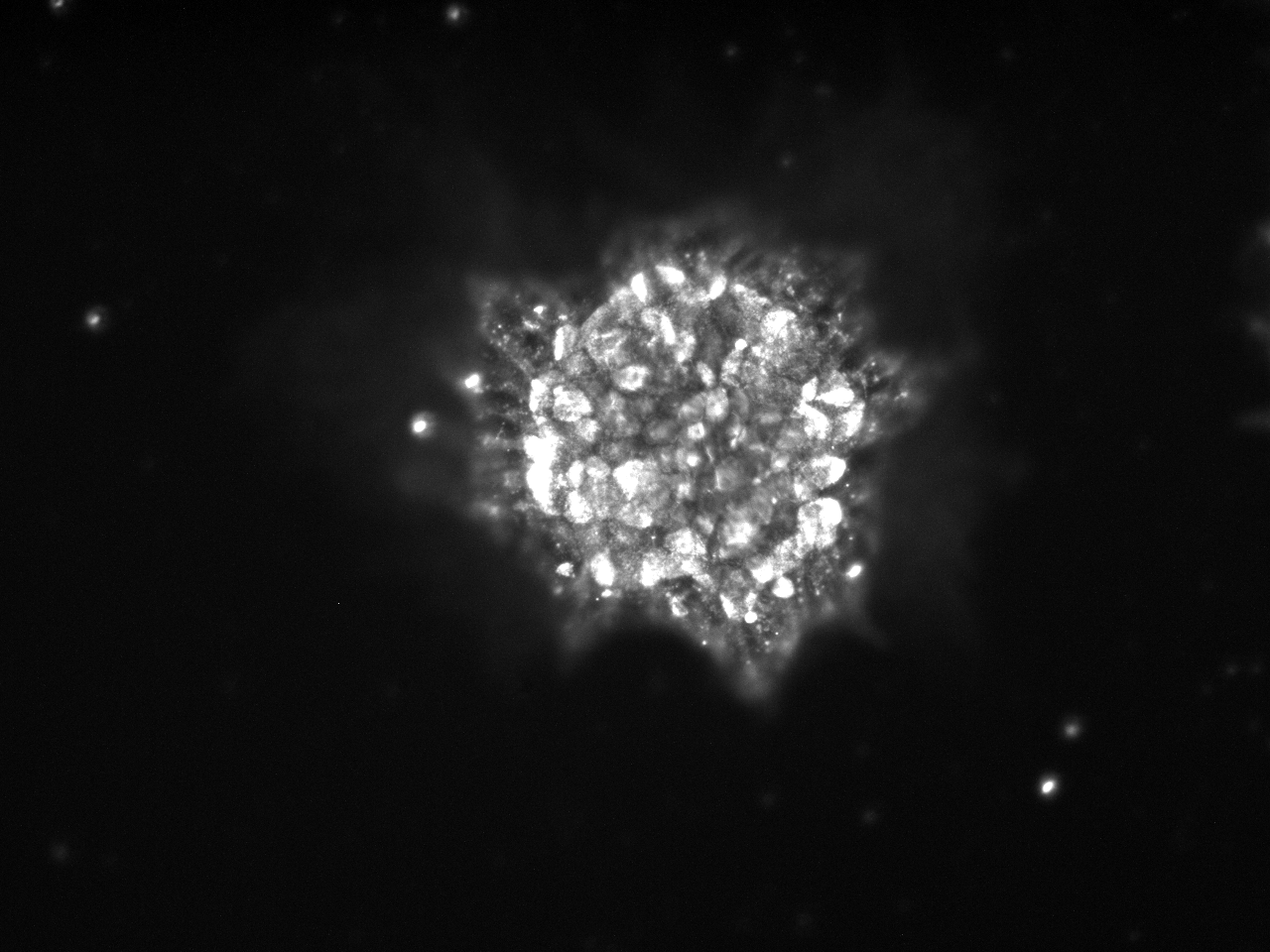

Supplement: Figure 10—source data 2. [file elife-104045-fig10-data2.zip › Figure 10B Source data iPSC IF/iPSC IF/unprocessed/tra2g - Green Light.tif]

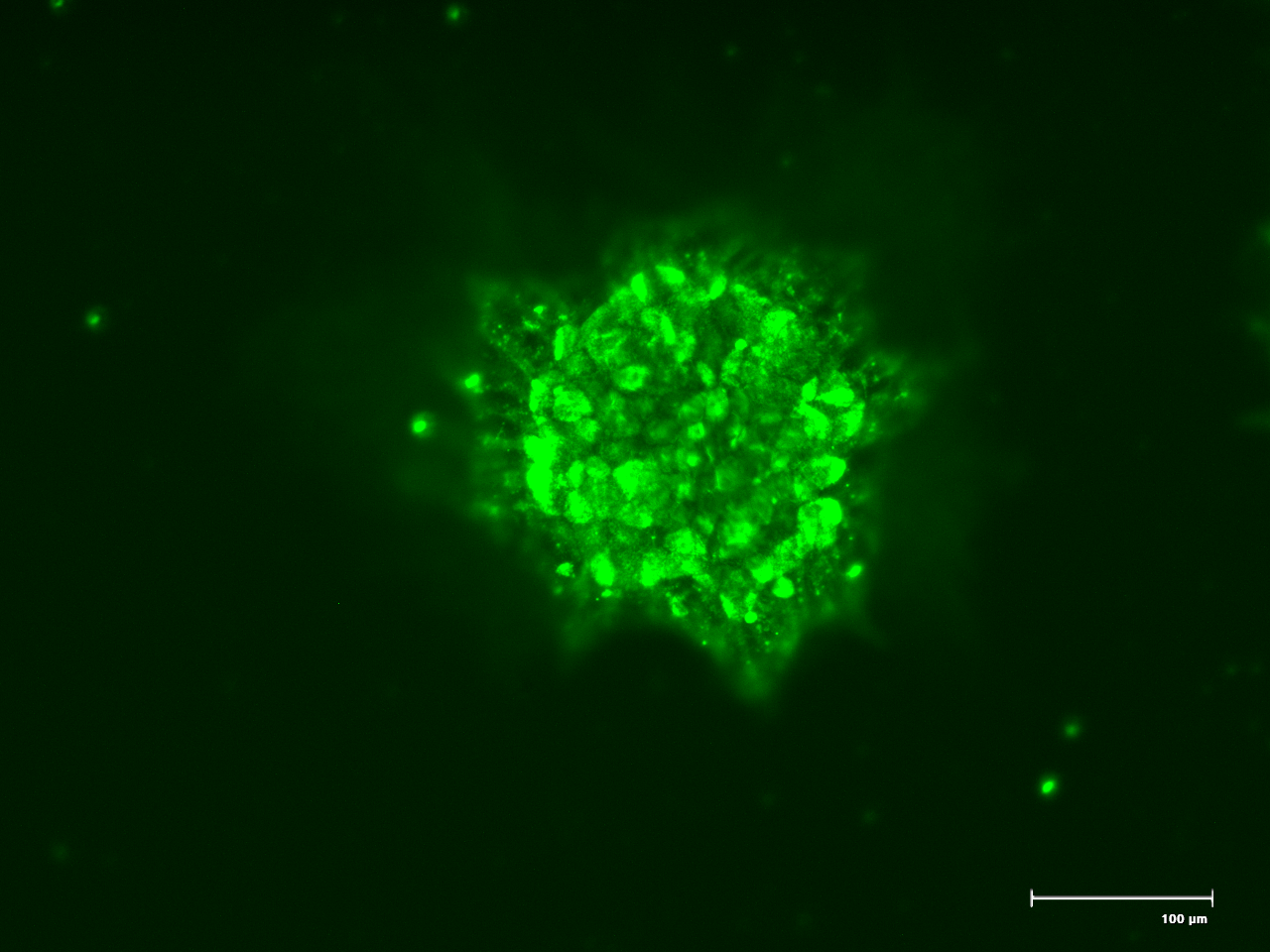

Supplement: Figure 10—source data 2. [file elife-104045-fig10-data2.zip › Figure 10B Source data iPSC IF/iPSC IF/unprocessed/tra2g.tif]

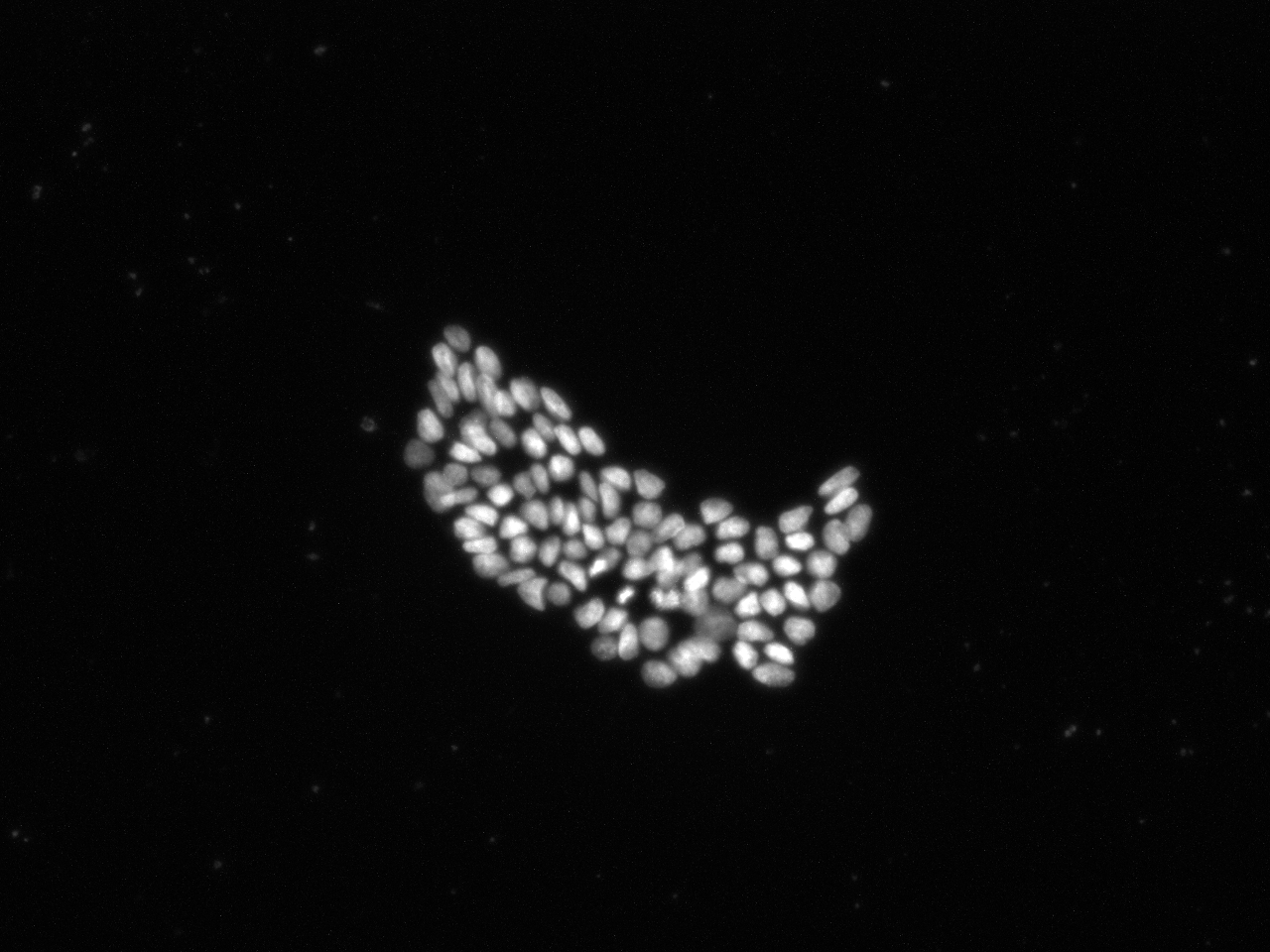

Supplement: Figure 10—source data 2. [file elife-104045-fig10-data2.zip › Figure 10B Source data iPSC IF/iPSC IF/unprocessed/tra3b - Blue Light.tif]

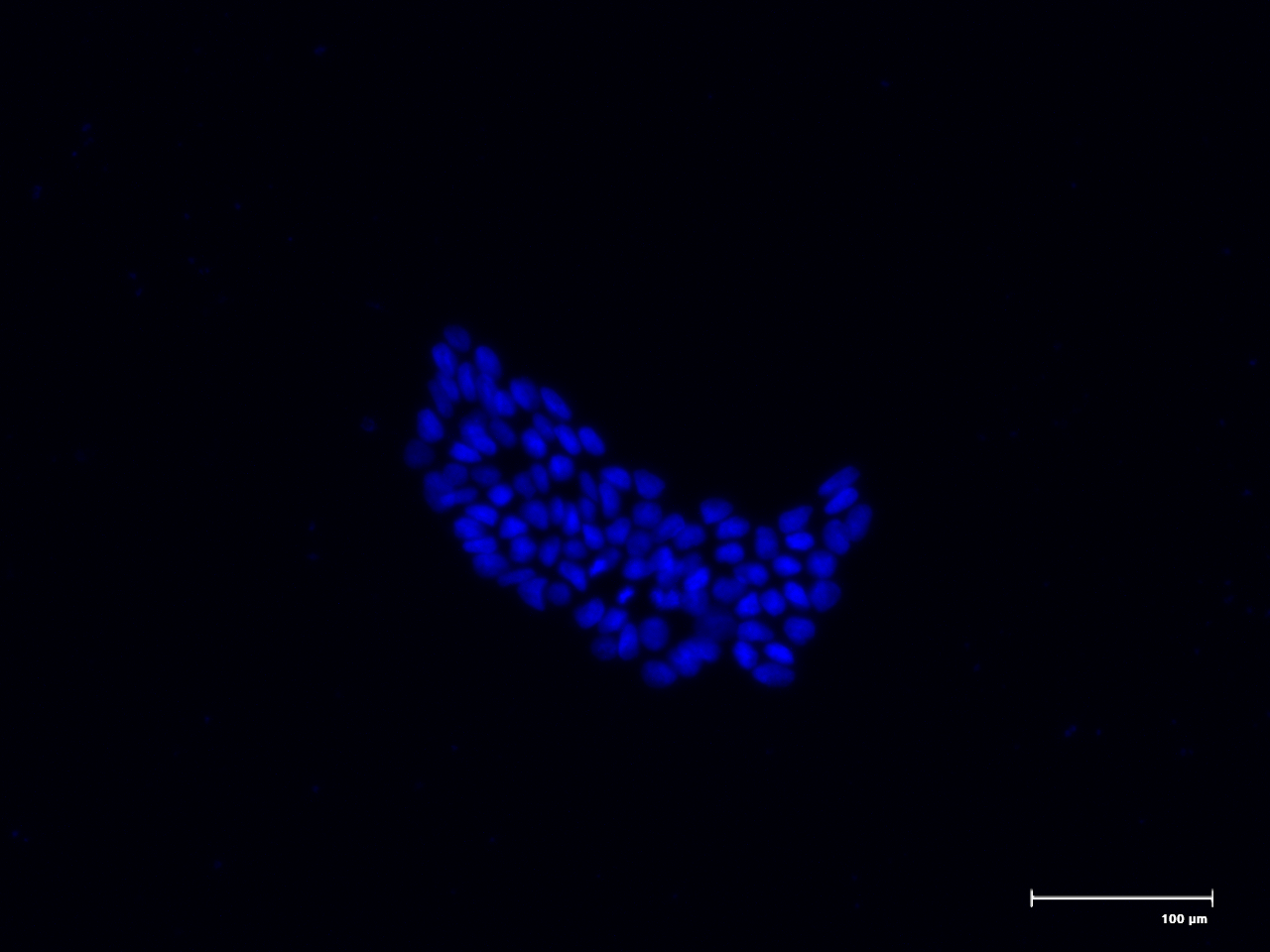

Supplement: Figure 10—source data 2. [file elife-104045-fig10-data2.zip › Figure 10B Source data iPSC IF/iPSC IF/unprocessed/tra3b.tif]

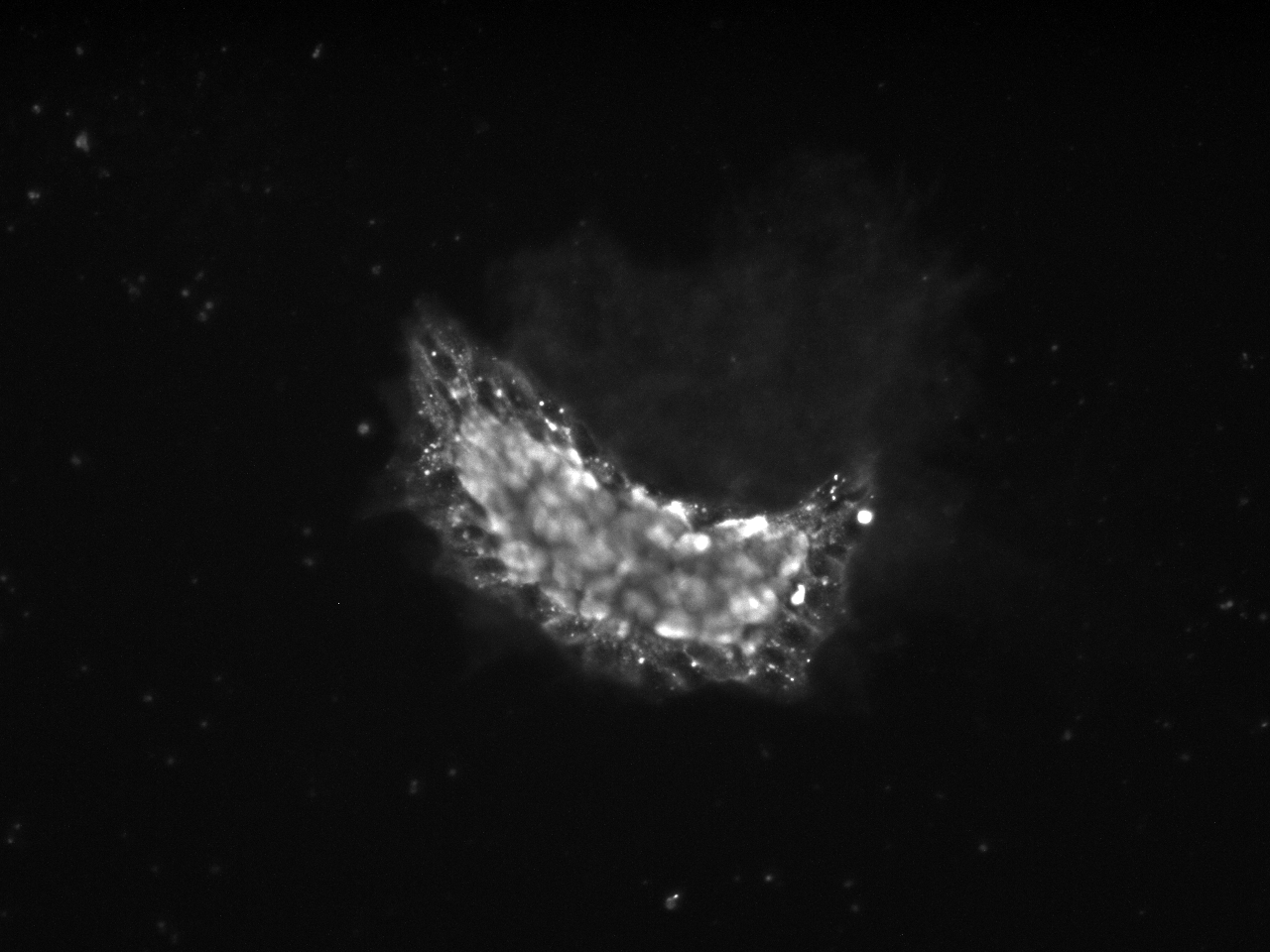

Supplement: Figure 10—source data 2. [file elife-104045-fig10-data2.zip › Figure 10B Source data iPSC IF/iPSC IF/unprocessed/tra3g - Green Light.tif]

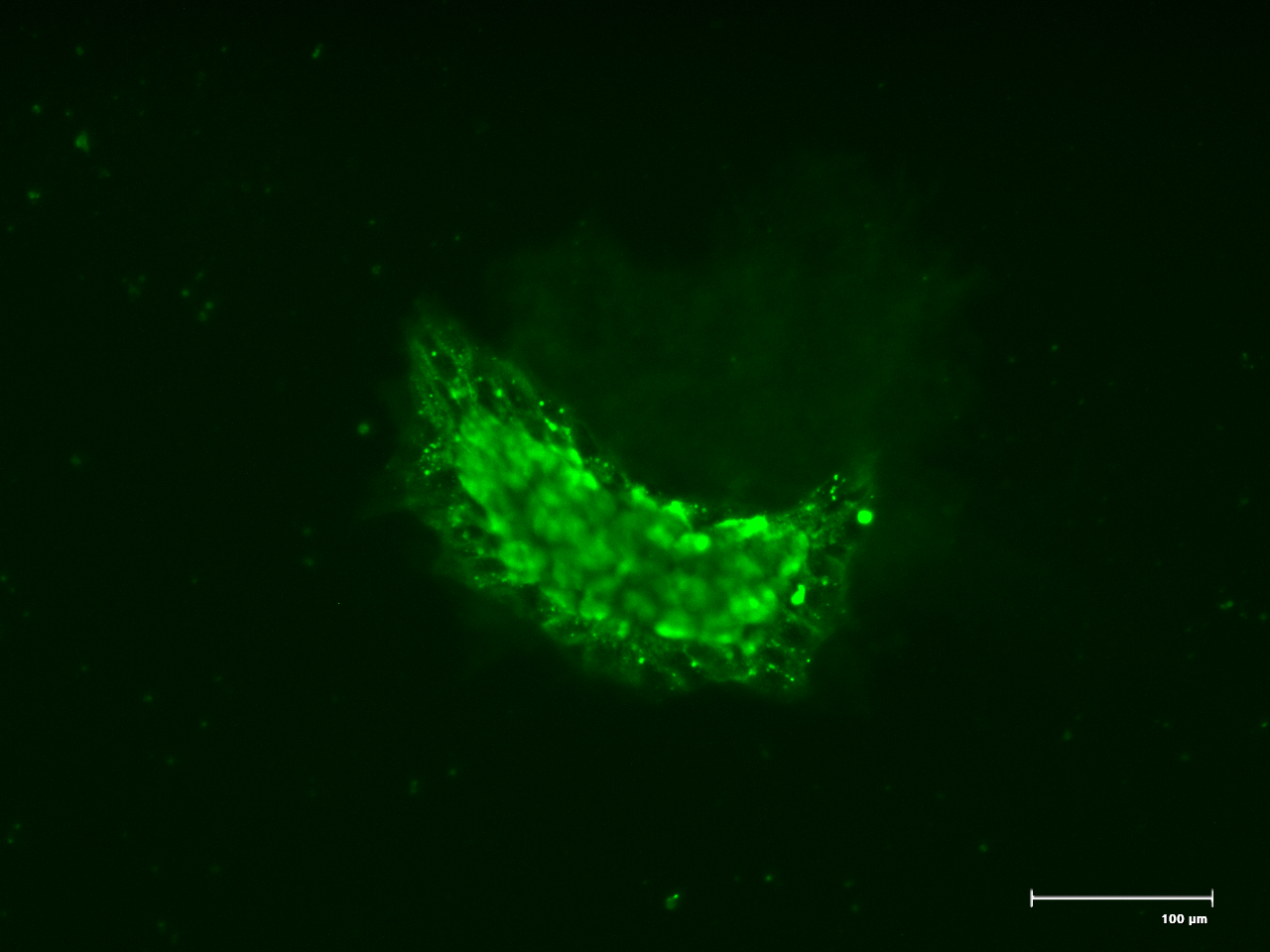

Supplement: Figure 10—source data 2. [file elife-104045-fig10-data2.zip › Figure 10B Source data iPSC IF/iPSC IF/unprocessed/tra3g.tif]

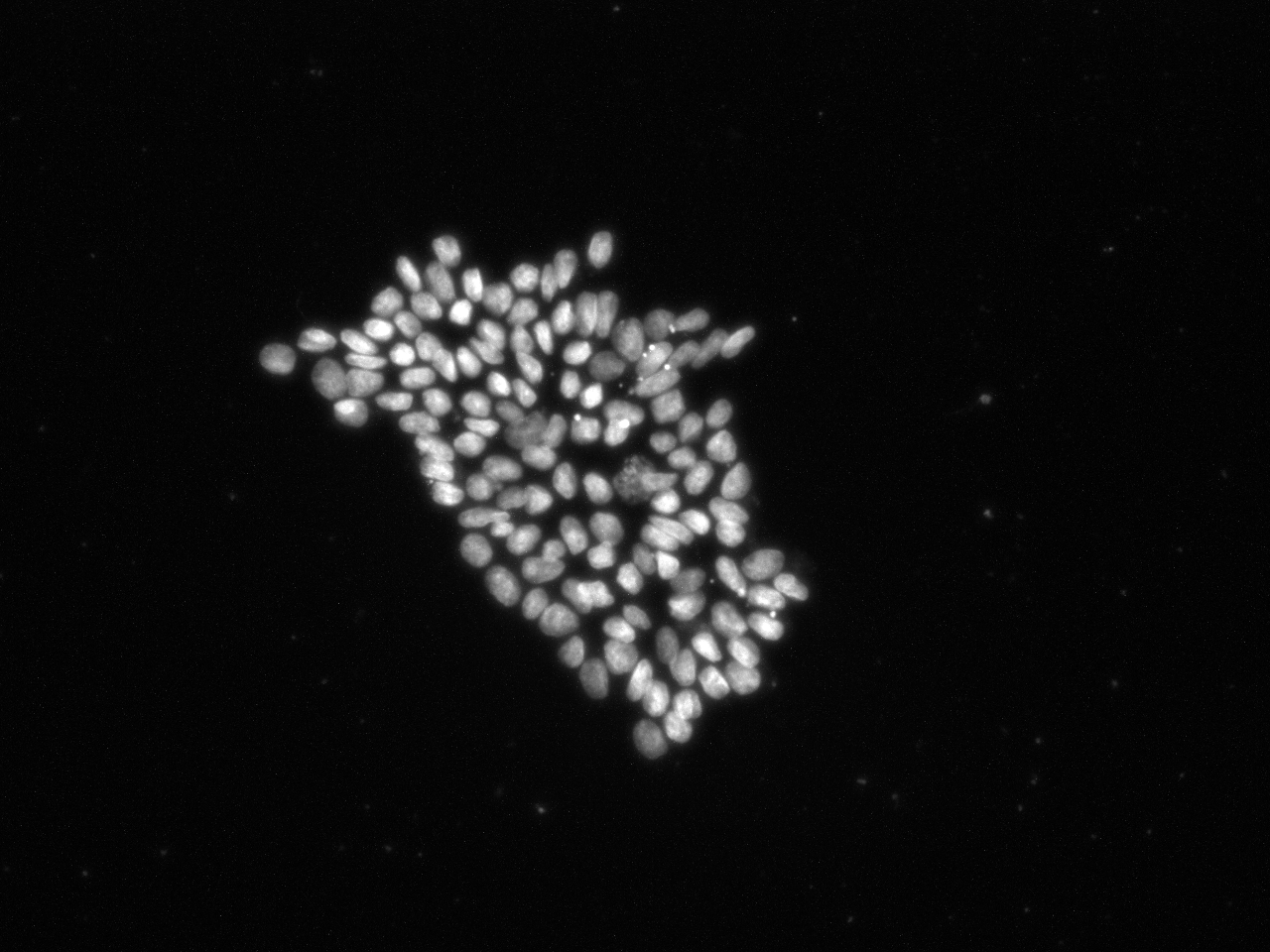

Supplement: Figure 10—source data 2. [file elife-104045-fig10-data2.zip › Figure 10B Source data iPSC IF/iPSC IF/unprocessed/tra5b - Blue Light.tif]

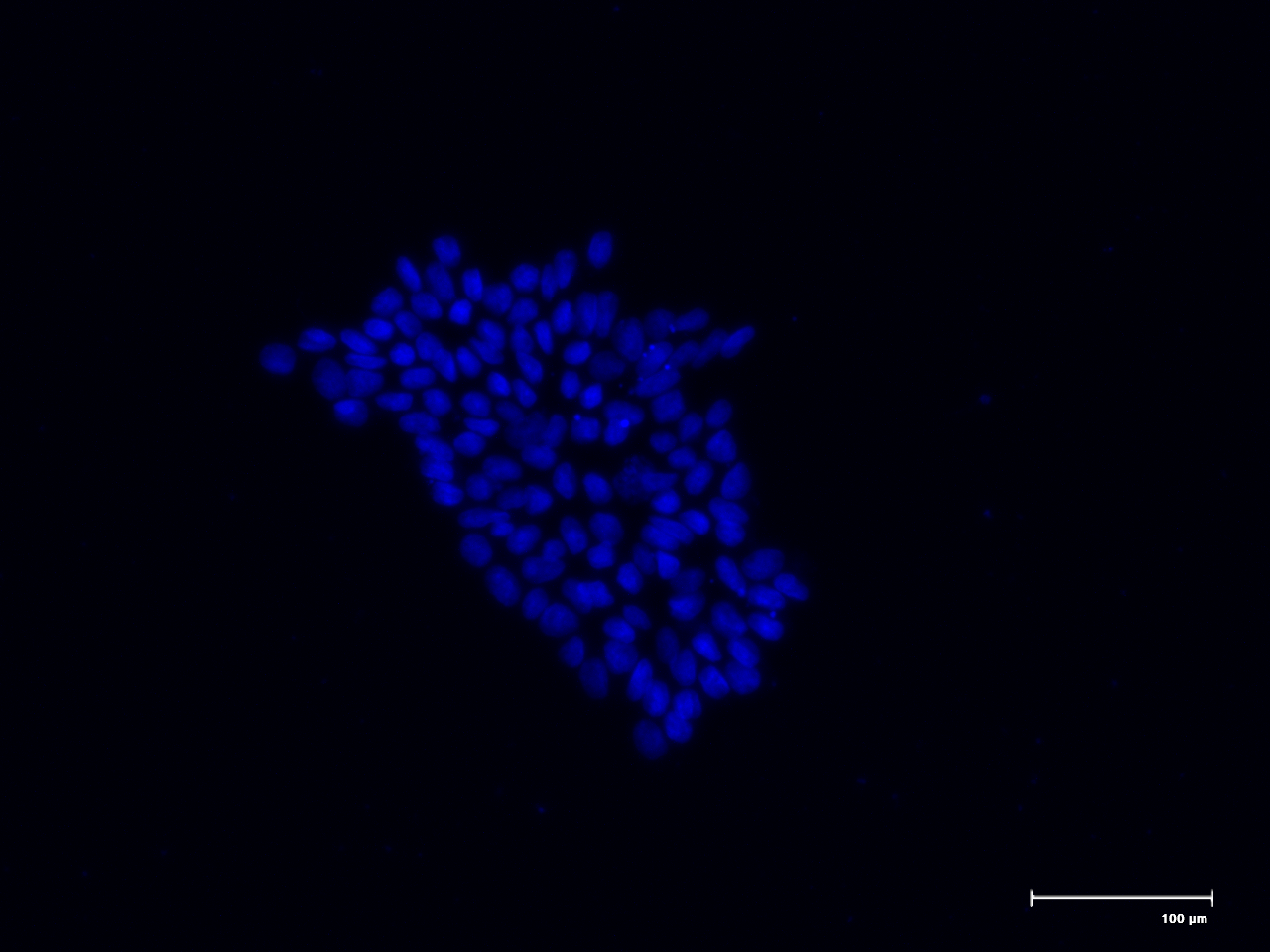

Supplement: Figure 10—source data 2. [file elife-104045-fig10-data2.zip › Figure 10B Source data iPSC IF/iPSC IF/unprocessed/tra5b.tif]

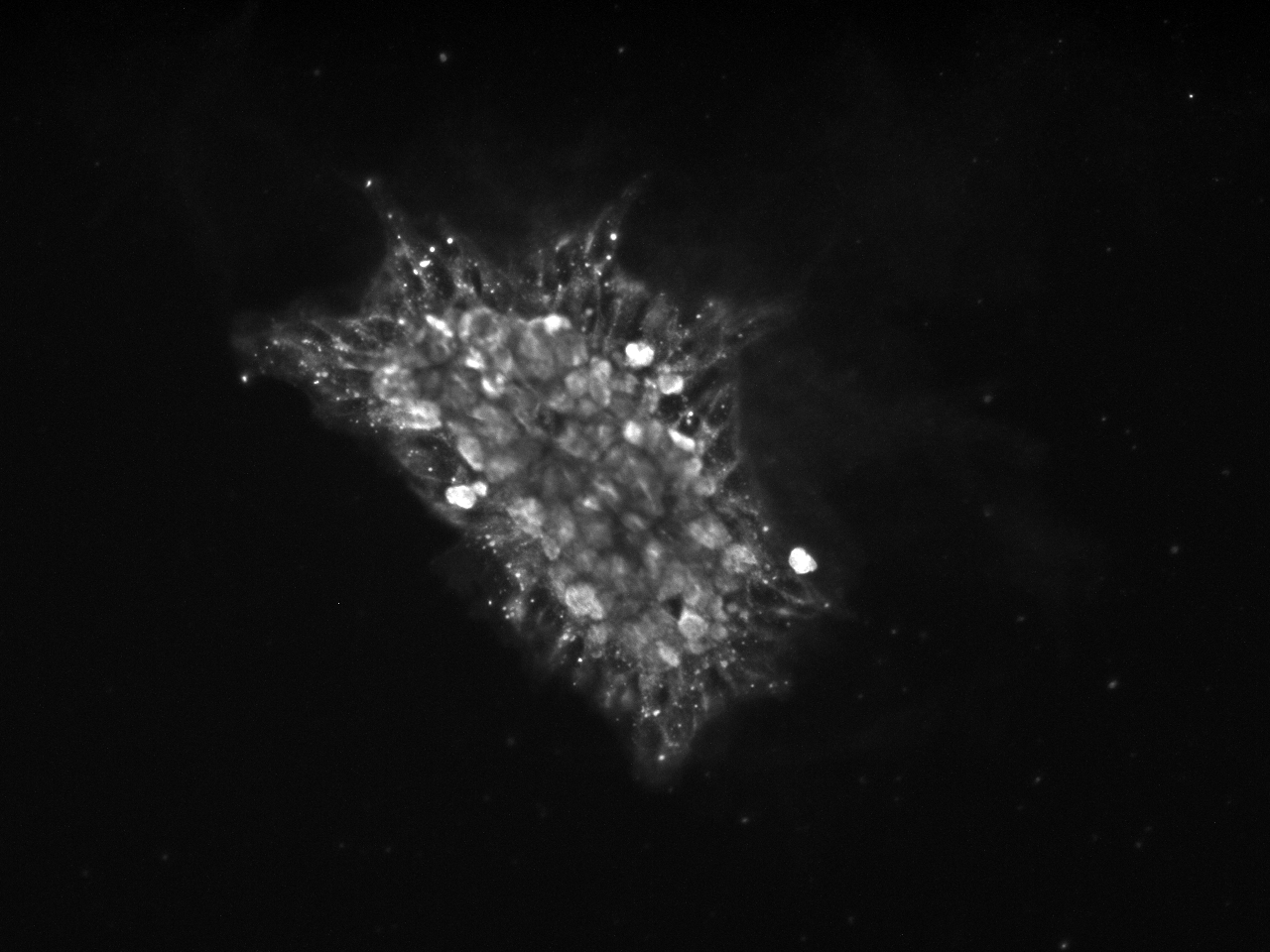

Supplement: Figure 10—source data 2. [file elife-104045-fig10-data2.zip › Figure 10B Source data iPSC IF/iPSC IF/unprocessed/tra5g - Green Light.tif]

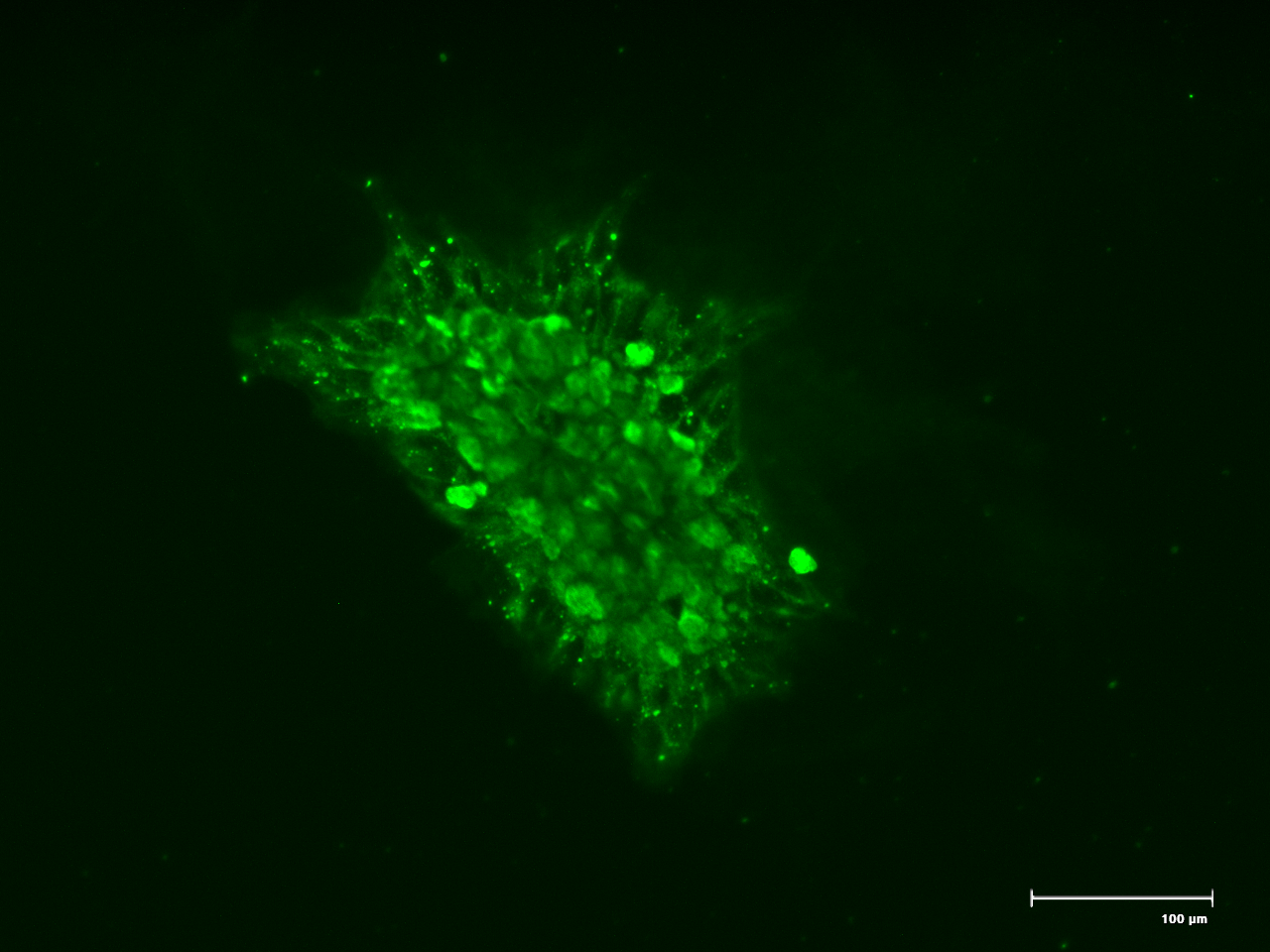

Supplement: Figure 10—source data 2. [file elife-104045-fig10-data2.zip › Figure 10B Source data iPSC IF/iPSC IF/unprocessed/tra5g.tif]

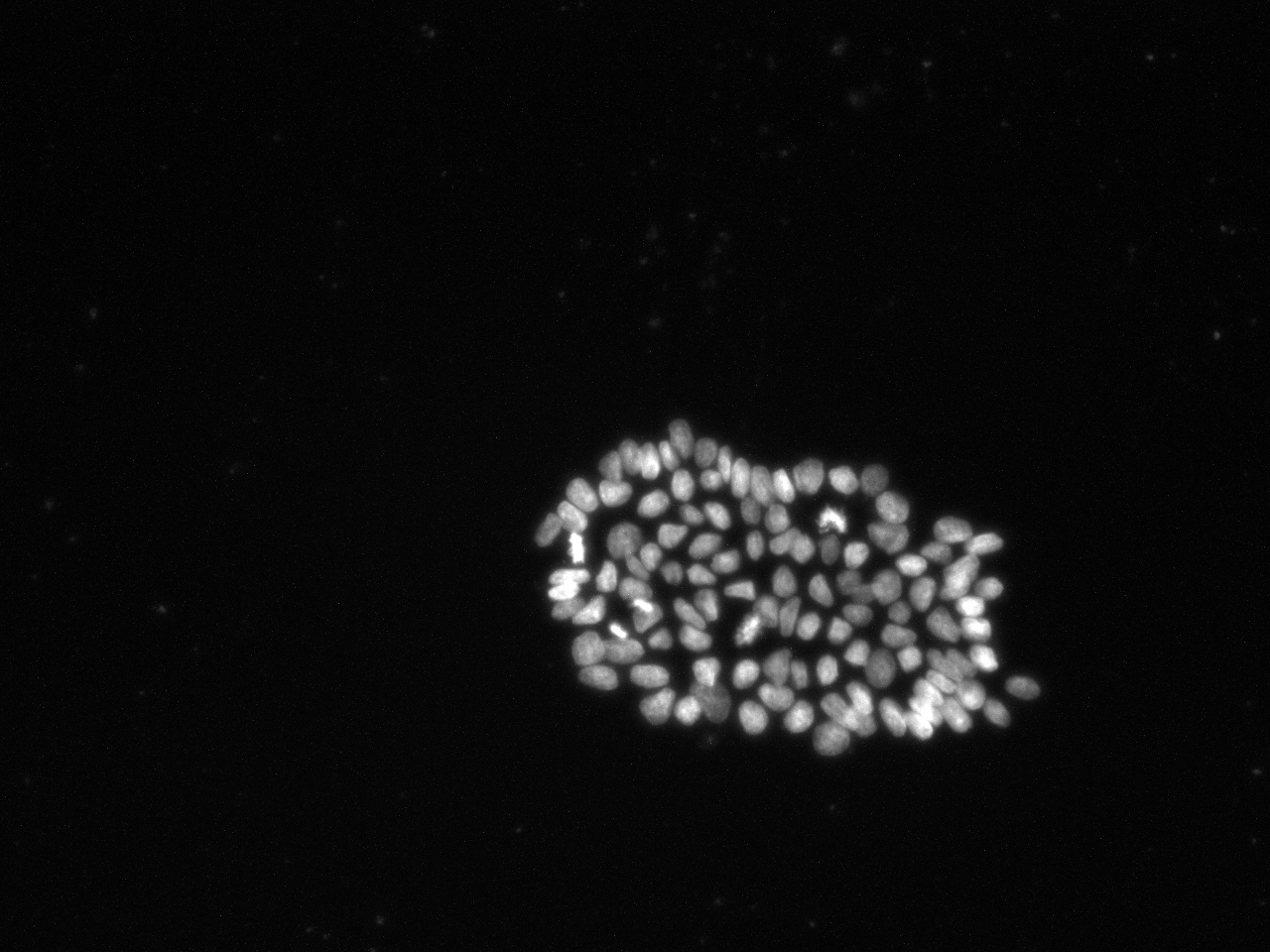

Supplement: Figure 10—source data 2. [file elife-104045-fig10-data2.zip › Figure 10B Source data iPSC IF/iPSC IF/unprocessed/trag - Blue Light.tif]

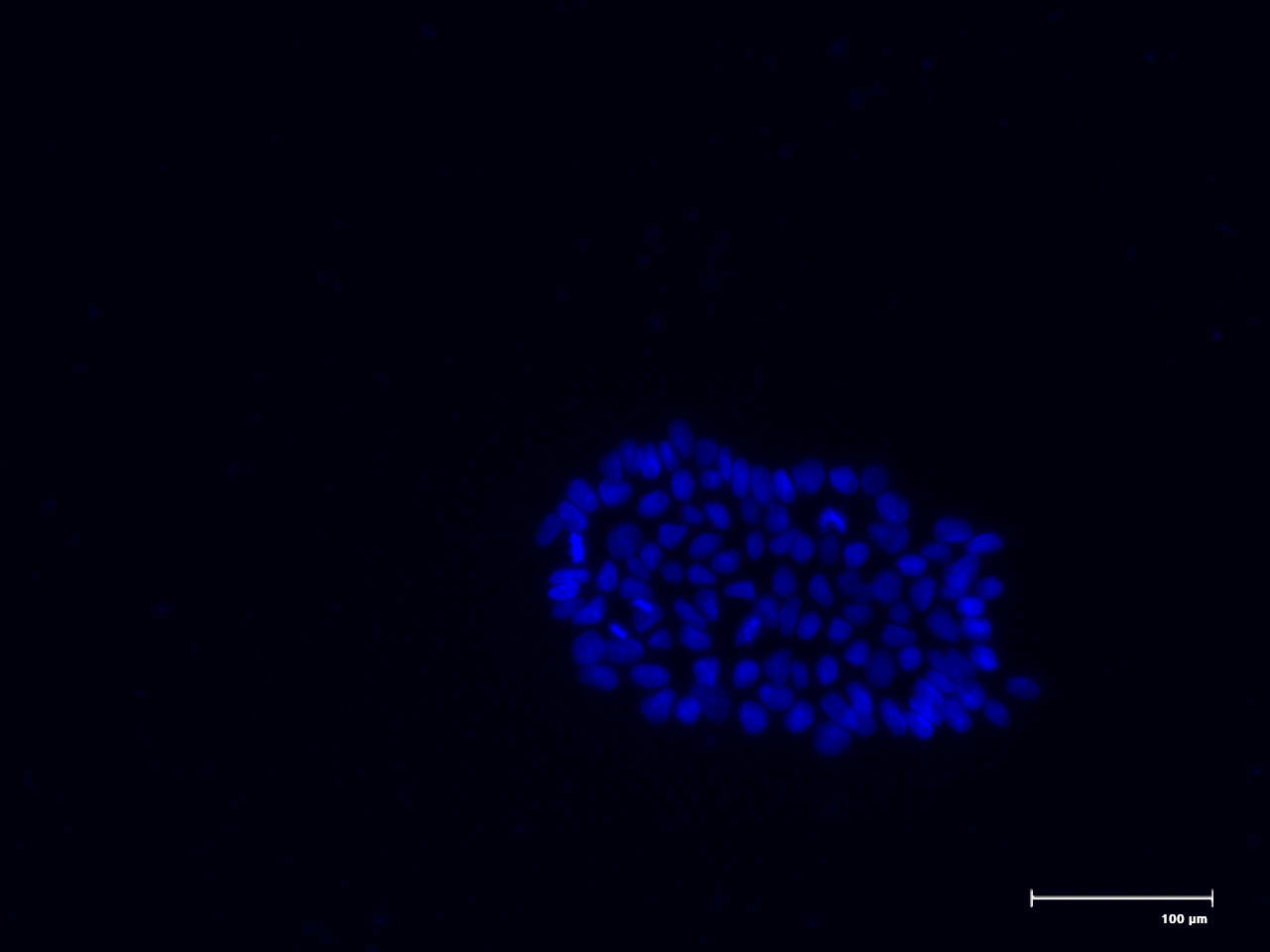

Supplement: Figure 10—source data 2. [file elife-104045-fig10-data2.zip › Figure 10B Source data iPSC IF/iPSC IF/unprocessed/trag.tif]

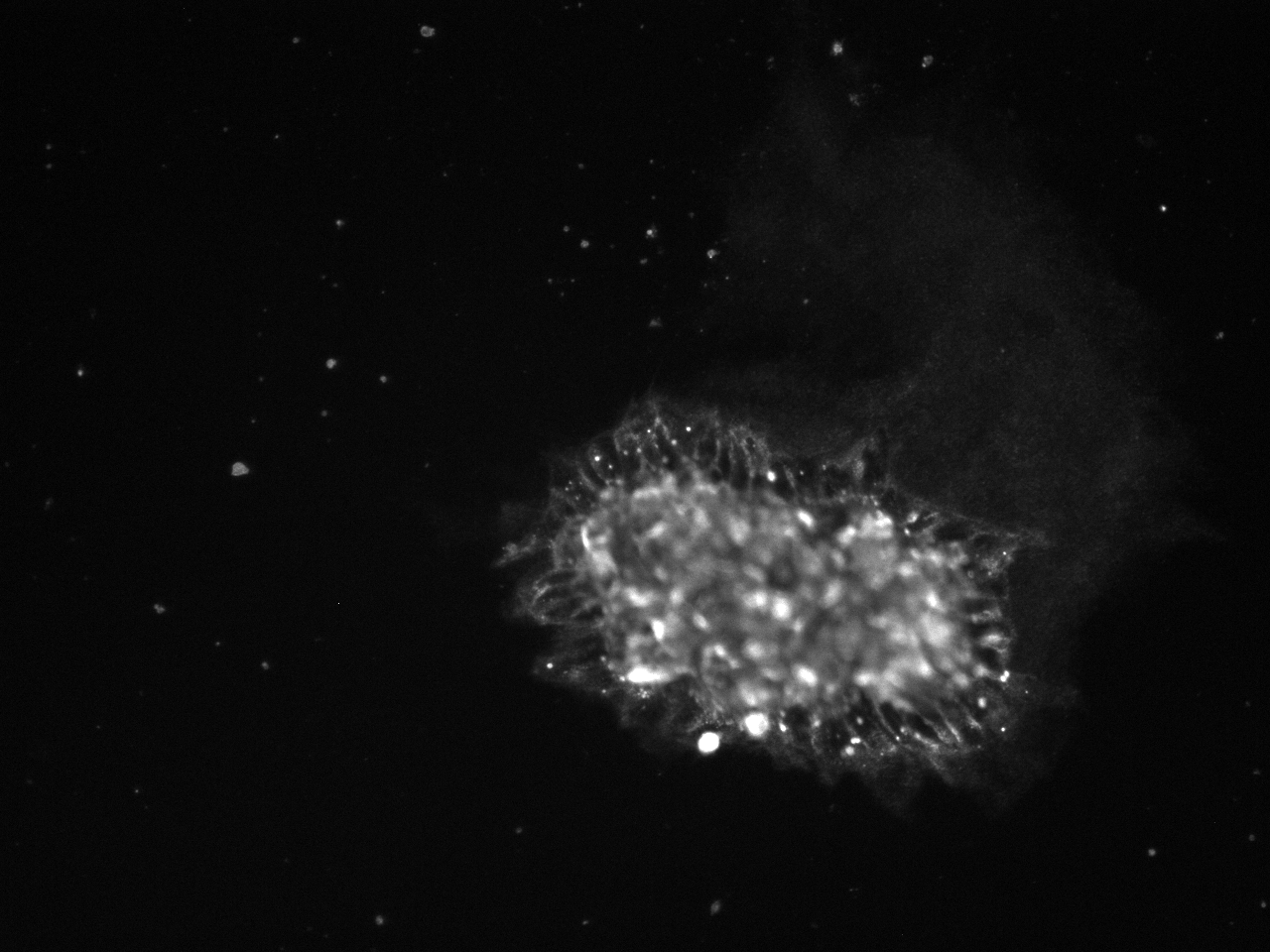

Supplement: Figure 10—source data 2. [file elife-104045-fig10-data2.zip › Figure 10B Source data iPSC IF/iPSC IF/unprocessed/tragg - Green Light.tif]

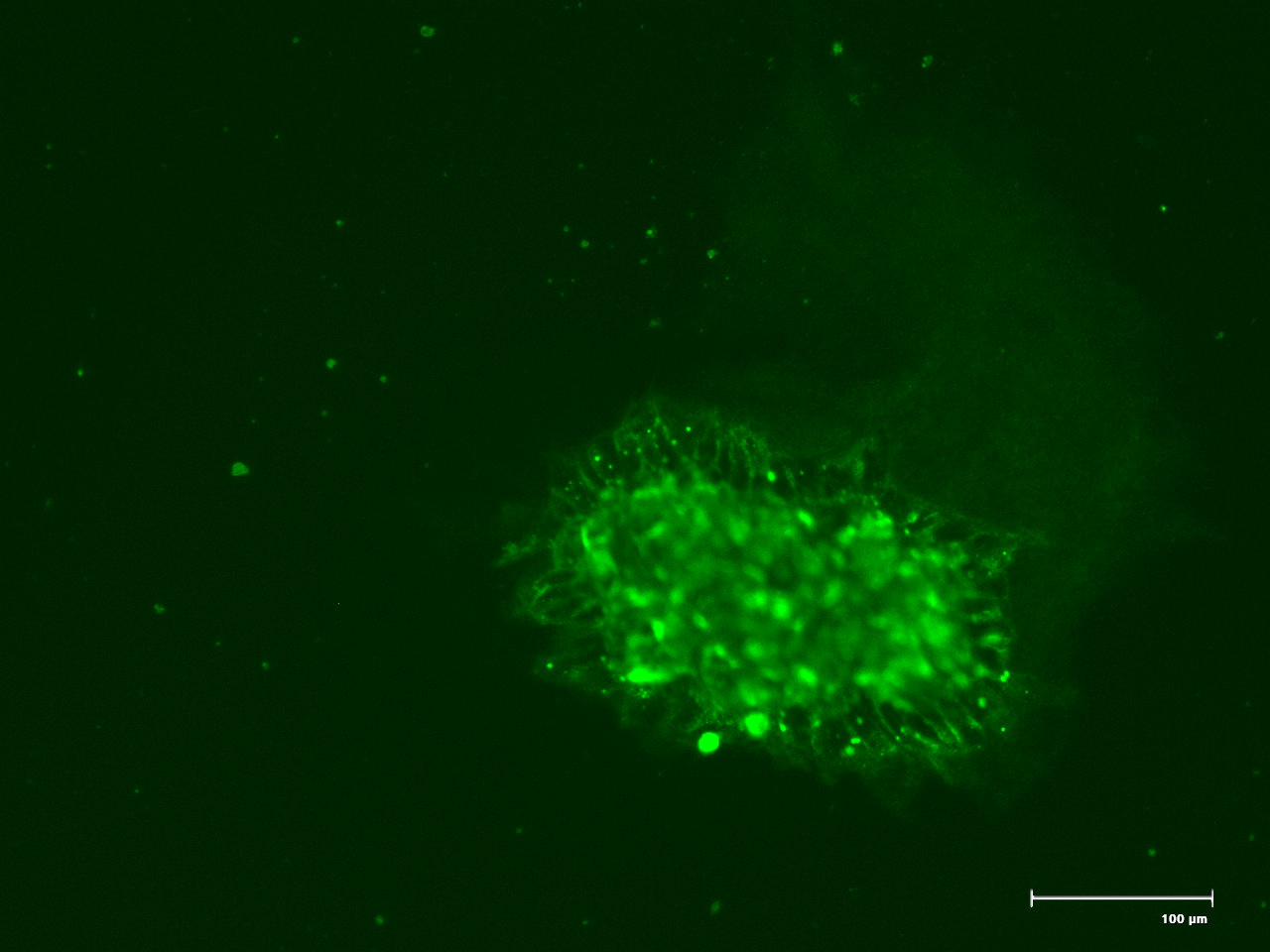

Supplement: Figure 10—source data 2. [file elife-104045-fig10-data2.zip › Figure 10B Source data iPSC IF/iPSC IF/unprocessed/tragg.tif]
